# Supplementary material for: Sitetack: a deep learning model that improves PTM prediction by using known PTMs
Source: Bioinformatics. 2024 Oct 10;40(11):btae602. doi: 10.1093/bioinformatics/btae602 (PMC11552626; doi:10.1093/bioinformatics/btae602)
Supplement: btae602_Supplementary_Data [file btae602_supplementary_data.pdf]

Sitetack: A Deep Learning Model that Improves PTM Prediction by Using Known PTMs

Clair S. Gutierrez[1,2,†], Alia A. Kassim[1,†], Benjamin D. Gutierrez, and Ronald T. Raines[1,2,3,*]

[1]Department of Chemistry, Massachusetts Institute of Technology, Cambridge, MA 02139, United States

[2]Broad Institute of MIT and Harvard, Cambridge, MA 02142, United States

[3]Koch Institute for Integrated Cancer Research at MIT, Cambridge, MA 02139, United States

*Corresponding author. E-mail: rtraines@mit.edu

[†]equal contribution

Content **Page**

S1. General Information
S1.1. Abbreviations S3
S1.2. Posttranslational Modifications S4
S1.3. Datasets S4
S1.4. Python Libraries and Code S5
S1.5. Website S5
S1.6. Statistics S5
S1.7. Amino Acid Alphabets and Encoding S6
S2. Data Preparation
S2.1. MusiteDeep Datasets S6
S2.2. OGP Datasets S6
S2.3. O-GlcNAc Site Atlas Datasets S6
S2.4. LMNglyPred Datasets S7
S2.5. Sugiyama Kinase Datasets S7
S2.6. Note on Dataset Biases S7
S2.7. Performance Assessment S8
S3. Frequency of Nearby PTM Sites
S3.1. Generation of PTM Frequency Graphs S9
S3.2. Frequency of Nearby PTM Sites in Human-Only Datasets from MusiteDeep S10
S3.3. Frequency of Nearby PTM Sites in All-Organism Datasets from MusiteDeep S14
S3.4. Frequency of Other O-Glycosylation Sites from OGP and O-GlcNAc Site Atlas S18
S3.5. Frequency of Other N-Glycosylation Sites from LMNglyPred Datasets S21
S3.6. Frequency of Other Phosphorylation (S,T) Sites from Sugiyama Kinase Datasets S22
S4. Tuning of Model Parameters
S4.1. Selection of Optimal "k" for k-mers S31
S4.2. Testing of Other Model Architectures S31
S4.3. Hyperparameter Tuning S33
S4.4. Additional Model Architecture Results S34

S5. Additional Model Results
S5.1. Model Results in Human-Only Datasets from MusiteDeep S36
S5.2. Model Results in All-Organism Datasets from MusiteDeep....................................S39
S6. Additional ROC curves
S6.1. AUC for Models Trained on MusiteDeep Datasets..S42
S6.2. AUPRC for Models Trained on MusiteDeep Datasets..S47
S7. Additional O-Glycosylation Models
S7.1. Model Results...S52
S7.2. AUC ROC Curves...S54
S7.3. AUPRC ROC Curves...S56
S8. N-Glycosylation Sequon-Specific Models
S8.1. Model Results...S58
S8.2. AUC ROC Curves...S58
S8.3. AUPRC ROC Curves...S59
S9. Additional Kinase-Specific Model Results
S9.1. List of Kinases Used..S59
S9.2. Kinase-Specific Model Results..S60
S10. Additional Cross-Model Figures
S10.1. O-GlcNAc Prediction with Phosphorylation (S,T) Sites Model Results S72
S10.2. Human O-GlcNAc Prediction with Phosphorylation (S,T) Sites Frequency and ROC graphs S73
S11. Effect of Homology on Model Performance
S11.1. Evaluation of Sequence Identity..S74
S11.2. Using CD-Hit to Lower Test-Set Identity...S75
S11.3. Using CD-Hit to Lower Test-Set Identity in OGP Datasets.....................................S85
S11.4. Using CD-Hit to Lower Test-Set Identity in N-Glycosylation Sequon-Specific Datasets..S88
S11.5. Using CD-Hit to Lower Test-Set Identity in O-GlcNAc Datasets S89
S12. Method Application to New Models
S12.1. TYOM: Train your own model instructions S90
S12.2. Example: Training models to predict phosphorylation in different contexts S91
S13. References S92

S1. General Information

S1.1. Abbreviations

Acc	accuracy
AUC	area under (ROC) curve
AUPRC	area under precision recall curve
CNN	convolutional neural network
DNN	deep neural network
F1	F1 score
FN	false negatives
FP	false positives
GalNAc	*N*-acetylgalactosamine
GlcNAc	*N*-acetylglucosamine
HexNAc	*N*-acetylhexosamine
LSTM	long short-term memory
MCC	Matthew's correlation coefficient
MLP	multilayer perceptron
Pre	precision
ReLu	rectified linear unit
RF	random forest
PCA	principal component analysis
PSIG	position-summed integrated gradients
PTM	posttranslational modification
RNN	recurrent neural network
ROC	receiver operating characteristic
Sn	sensitivity or recall
Sp	specificity
SVM	support vector machine
TN	true negatives
TP	true positives
t-SNE	t-distributed stochastic neighbor embedding
TYOM	train your own model

S1.2. Posttranslational Modifications

The chemical structures of the thirteen posttranslational modifications considered in this work are indicated in red below. Note that only monoglycosylation is shown for N- and O-glycosylation.

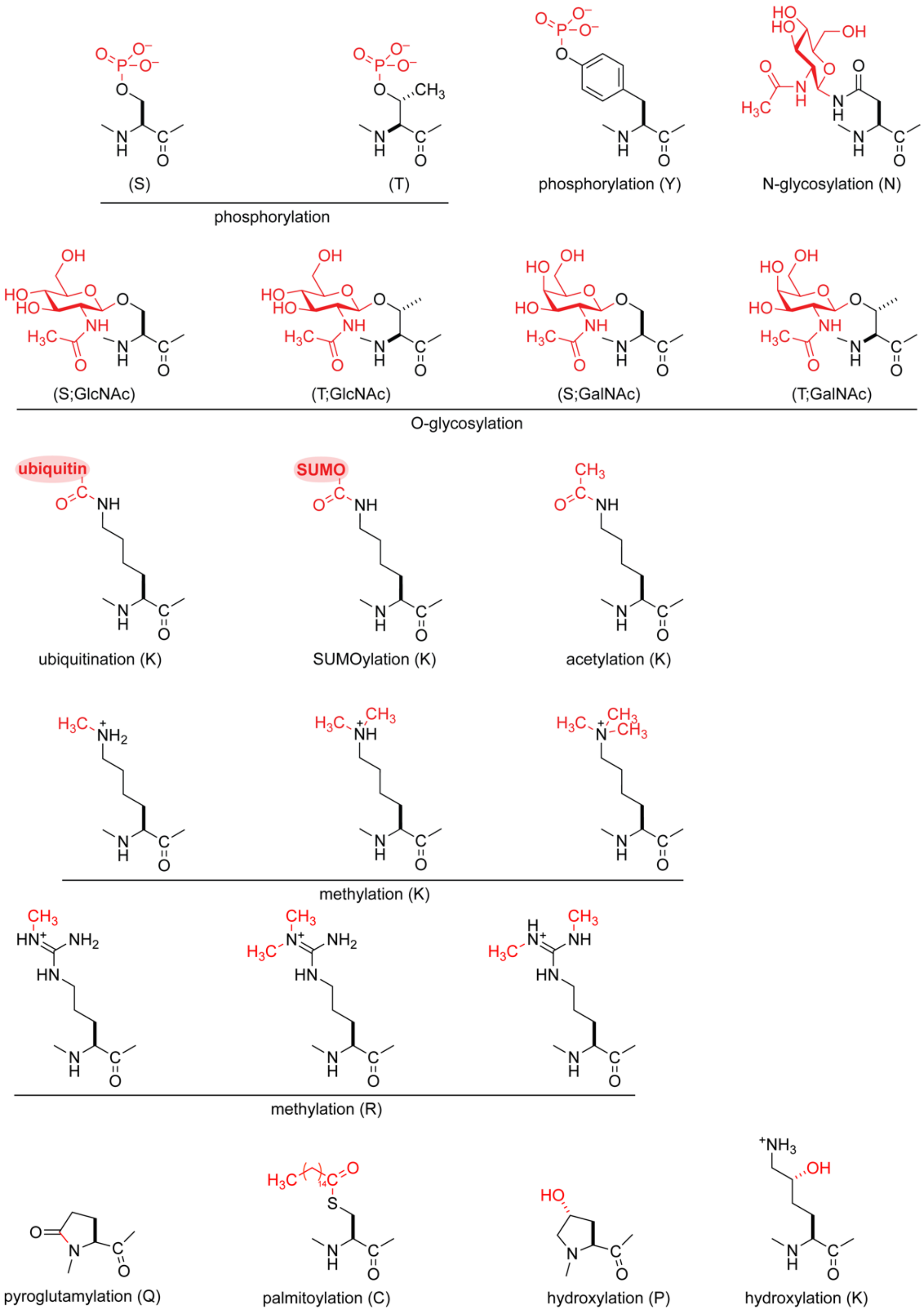

S1.3. Datasets

For information specific to the datasets used herein, see Section S2. Unless specified otherwise, the datasets were processed as follows. For each dataset, a list of Uniprot IDs of the modified proteins with a list of sites for each protein was generated. Additionally, a FASTA file of reference sequences was obtained. For

human protein models, this file was the UP000005640 reference proteome. For all-organism models, this file was generated by using the ID-mapping function on Uniprot (https://www.uniprot.org/id-mapping; accessed 7 February 2024) and inputting a list of all of the current Uniprot IDs of the modified proteins. Generally, isoforms and archaic proteins were removed automatically.

Using these files, a list of positive k-mers was generated from the reference sequences and the list of protein IDs and sites. Negative k-mers were generated by taking a list of all the possible PTM sites (based on what amino acid is modified) and removing sites contained in the list of known PTM sites. From these sites, the same number of negative k-mers as positive k-mers were chosen randomly. In the case of PTMs that could occur at two residues (S and T), then the ratio of sites between the two residues in the positive k-mers was maintained in the negative k-mers. Adding the locations of known PTMs to the k-mers was done by modifying a dictionary of Uniprot IDs and the cognate sequences from the sequence file to change PTM locations to a separate amino acid. Then, positive and negative k-mers with known PTM locations could be generated from this modified dictionary. For these the center amino acid, for which prediction occurs, was converted back to the unmodified version in the positive k-mers (otherwise, we would already be giving the model the correct answer).

For 10-fold Monte Carlo cross validation, a new set of negative k-mers was randomly selected for each repeat and combined with the positive k-mers (with labels as to whether they were negative or positive). These were then shuffled and split into training (80%), validation (10%), and test (10%) datasets. To ensure the test datasets were distinct, k-mers that were identical to any k-mer in the cognate train and validation sets were removed before evaluation.

S1.4. Python Libraries and Code

All module versions for python, libraries versions, and other programming information can be found in the Github repository for this work at https://github.com/clair-gutierrez/sitetack. In short, Jupyter Notebook was used for many of the model training and testing scripts. The TensorFlow library, often with the Keras API, was used for model creation and training. Numpy and pandas libraries were used for dataset manipulation. The Sci-kit learn library was used to analyze model results, generate PCA and t-SNE plots, and perform clustering. The matplotlib library was used to generate figures in Python, which were then further refined with Adobe Illustrator. Microsoft Excel was used to visualize data. Models were trained either locally or on the MIT Engaging cluster.

S1.5. Website

The Sitetack webserver consists of a three−layer architecture: logic layers, backend, and the front end. The prediction is done using the models written in python. The backend was written in the FastAPI python library for integration of the deep-learning models with the webserver. The front end and user interface were built using JavaScript and the JavaScript library Bootstrap. The server is hosted using Google Cloud Platform (GCP).

S1.6. Statistics

Reported values were the average of 10 independent validation replicates, each with its own train, test, and validation set. Error is reported as ± standard deviation (SD) of these replicates. P Values were calculated by a two-tailed Student's t-test using a paired test in Microsoft Excel or the Scipy library in python. In the case of Cross models, since the data was no longer paired, an unpaired t-test with unequal variance was used. A cutoff of $P < 0.05$ was used for significance. For every model trained the results along with the SD and P value can be found in the Supplementary Sections S4.4, S5.1, S5.2, S7.1, S8.1, S9.2, and S10.1.

S1.7. Amino Acid Alphabets and Encoding

In sequence datasets and k-mers amino acids were represented using their one-letter code as is standard. For each model we used an "alphabet" that was a list of all amino acids that might be included in a dataset. These include the 20 canonical amino acids, selenocysteine (U), and a padding or empty amino acid encoded as "-", which was used to pad sites that were close to the beginning or end of a sequence. PTMs in the models where their locations were used were encoded as "@" (and if another amino acid could be modified "&"). Additionally, some datasets included degenerative amino acids: "X" for any amino acid, "B" for aspartic acid or asparagine, and "Z" for glutamic acid or glutamine. These were included in the alphabets for those datasets to be encoded as their own amino acid (allowing the word embedding to learn their relationships). The alphabets for each dataset and model can be found at https://github.com/clair-gutierrez/sitetack /Amino_acid_alphabets.xlsx. Each letter code in an alphabet was then used to transform a k-mer into a numerical vector. This vector was inputted into a word embedding layer that had a max vocabulary (number of residue types allowed) size that was much greater (256) than the length of any alphabet used to allow for an arbitrary amount of additional amino acids to be added (in the case of additional PTMs), with an output dimension of 21 for all models. For example, the sequence "ACD" would become <1, 2, 3>, then after word embedding a vector of dimension [3, 21]. The specific transformation is learned for each model (as opposed to one-hot encoding, which is hardcoded and invariant) and would ideally place related residues (words) closer in vector space.

S2. Dataset Preparation

S2.1. MusiteDeep Datasets

The datasets used by MusiteDeep (Wang *et al.* 2020) were downloaded from musite.net. These datasets consisted of a FASTA file with amino acids that had the PTM followed by a "#". We then generated a file of the Uniprot ID of the sequence in one column with all the modified site locations in the next column, along with a FASTA file without the "#", which was used as the reference sequence file for the all-organism models.

S2.2. OGP Datasets

The dataset from the OGP (Huang *et al.* 2021) was downloaded from http://www.oglyp.org/download.php (accessed 7 February 2024). To access the entire database (specifically for information on HexNAc versus GalNAc glycans), we contacted the authors directly. For the whole glycosylation datasets, the datasets downloaded were already a file with the UniProt ID with the site location. For the HexNAc and GalNAc datasets, we split the whole database (which includes that information and less validated sites) into subsets of sites with HexNAc or GalNAc. These were then cross-referenced with the main set to only include validated sites.

S2.3. O-GlcNAc Site Atlas Datasets

The dataset from O-GlcNAcAtlas (Ma *et al.* 2021) was downloaded from https://oglcnac.org/atlas/download/ (accessed 12 February 2024). Only the "Unambiguous sites" dataset was used, and the dataset downloaded was already a file with the UniProt ID with the site location. This dataset had duplicates (same Uniprot ID and site location), so k-mers were only added to the set of positive k-mers if they did not already exist in the set of positive k-mers.

S2.4. LMGglyPred Datasets

The datasets from LMNglyPred (Pakhrin *et al.* 2023) were downloaded from their Github at https://github.com/KCLabMTU/LMNglyPred (accessed 2 February 2024). These datasets included both the NGlycosite Atlas (Sun *et al.* 2019) and NGlyDE (Pakhrin *et al.* 2021) sets. The datasets downloaded were already a file with the UniProt ID having the site location. Given that the NGlyDE dataset was already for human proteins and the NGlycosite Atlas dataset was for all organisms, we made a human protein model only from the NGlyDE dataset and an all-organism model from the NGlycosite dataset. For the NGlycosite dataset, there were many isoforms, so all of the isoform sequences on Uniprot were downloaded, and isoforms in the dataset were added to the reference sequences.

Because this set already had negatives in a similar ratio to positives, no negatives were generated as in the other datasets, and no additional balancing of the ratio of negatives to positives was performed. These datasets were already split into test and train sets. For the first replicate, this split was preserved, but for all subsequent validation replicates, the sets were combined, shuffled, and split again.

S2.5. Sugiyama Kinase Datasets

The experimental dataset from Sugiyama (Sugiyama *et al.* 2019) was chosen due to the large number of kinases that were assayed (385), the number of phosphosites obtained (175, 574), and that it was done in whole cell lysates not peptides. This was not a curated dataset, but phosphosites were obtained from a standard and stringent method in this study. Additionally, this dataset has been used by several other studies and prediction models.

The data were downloaded from https://www.nature.com/articles/s41598-019-46385-4#Sec21 (accessed 29 November 2023). These data included the kinase and its potential substrates and the modified sites and only sites that were unambiguous using the site-determining ion combination method or had a PTM score of >0.75 were used. These data were then imported into Python, and the number of sites per kinase were tabulated. A set of kinases with over 500 sites was generated, as a large number of sites is necessary for successful training and there were no S,T kinases with over 1000 sites. From this set of kinases with over 500 sites, the kinases, which mainly phosphorylated tyrosine residues (using a cutoff of 50% of the sites), were then removed from the set, resulting in a set of 68 kinases that phosphorylated primarily at S,T. In these remaining kinase datasets, all of the tyrosine sites were then removed.

Given that all of the kinases were human, the reference proteome UP000005640 was used to get the sequences and generate negatives. Once the 68 kinase datasets were prepared, the training, test, and 10-fold Monte Carlo cross validation sets were prepared for each kinase, as in Section S1.

S2.6. Note on Dataset Biases

For any machine learning model, prediction quality is highly dependent on dataset quality. Though all of the main datasets used in this study have been used in various prediction algorithms and are highly curated, they still have biases that result in biases in the trained models and hence decreased applicability to researchers. Here, we attempt to be transparent on these limitations.

In terms of organism scope, all the datasets used are highly skewed towards human and model mammal (*e.g.*, *M. musculus*) proteins and do not contain non-eukaryotic proteins. The all-organism models that we trained generally increased the training set size, often allowing for better training, but likely suffer from decreased accuracy in non-mammalian systems. Human proteins were often the most frequent, so we trained separate models for these to allow for organism specificity in one organism. We hope that with

future research in PTM sites in other organisms, better organism-specific datasets can be made for a large diversity of life, allowing better prediction in those organisms.

In terms of which proteins are represented, in general the datasets used suffer from detection biases. It is likely that more abundant proteins are over-represented due to mass spectrometry detection limits in the large-scale proteomic studies that are often used to discover PTM sites. To address this bias, we do filter k-mers by sequence similarity between the training, validation, and test sets to assess that bias. On the flip side, this experimental insensitivity is a benefit of PTM prediction, which can be used to predict PTMs on less abundant proteins. In some datasets, there are over-representations of certain protein families (*e.g.*, collagens for Hydroxylation (P)), but we did not reduce that over-representation because of its reflecting intrinsic preferences of the enzymes that install or remove these PTMs in specific sequences. In short, some protein biases in the dataset are due to PTM enzyme(s) specificity and others are experimental biases, and the difference can be difficult to know *a priori*.

S2.7. Performance Assessment

To assess the prediction performance of Sitetack, we used several common metrics, including area under the ROC curve (AUC), area under the precision recall curve (AUPRC), accuracy (Acc), sensitivity or recall (Sn), specificity (Sp), precision (Pre), Matthew's correlation coefficient (MCC), and F1 score (F1). Whereas AUC and AUPRC were calculated based on taking the area under the curve generated by plotting the false positive rate against the true positive rate or the precision against the recall respectively of a dataset, the remaining metrics were defined by the equations:

$$\mathrm{Acc} = \frac{\mathrm{TP} + \mathrm{TN}}{\mathrm{TP} + \mathrm{TN} + \mathrm{FP} + \mathrm{FN}}$$

$$\mathrm{Sn} = \frac{\mathrm{TP}}{\mathrm{TP} + \mathrm{FN}}$$

$$\mathrm{Sp} = \frac{\mathrm{TN}}{\mathrm{TN} + \mathrm{FP}}$$

$$\mathrm{Pre} = \frac{\mathrm{TP}}{\mathrm{TP} + \mathrm{FP}}$$

$$\mathrm{MCC} = \frac{\mathrm{TP} \times \mathrm{TN} - \mathrm{FP} \times \mathrm{FN}}{\sqrt{(\mathrm{TP} + \mathrm{FN}) \times (\mathrm{TP} + \mathrm{FP}) \times (\mathrm{TN} + \mathrm{FN}) \times (\mathrm{TN} + \mathrm{FP})}}$$

$$\mathrm{F1} = \frac{2 \times \mathrm{Pre} \times \mathrm{Sn}}{\mathrm{Pre} + \mathrm{Sn}}$$

where TP, TN, FP, and FN refer to true positives, true negatives, false positives, and false negatives, respectively. For every model trained, the results along with the SD and *P* value can be found in Supplementary Sections S4.4, S5.1, S5.2, S7.1, S8.1, S9.2, and S10.1. Performance was evaluated using an independent test set that had no overlap (<100% sequence similarity) with the training and validation sets to match the majority of the comparator models. Additional performances using test sets at different sequence similarity reductions can be seen in Supplementary Section S11 with results explicitly reported for an 80% similarity reduction.

S3. Frequency of Nearby PTM Sites

S3.1. Generation of PTM frequency graphs

To determine how often PTMs appeared next to other PTMs, we pooled all of the positive k-mers for a given PTM and tabulated the pairwise distances. These distances are represented as frequency graphs from the prediction amino acid (k-mer center).

To capture every pairwise distance between PTMs only once, we considered only PTMs that were C-terminal to the k-mer center, thereby avoiding double-counting of residues N-terminal to the k-mer center.

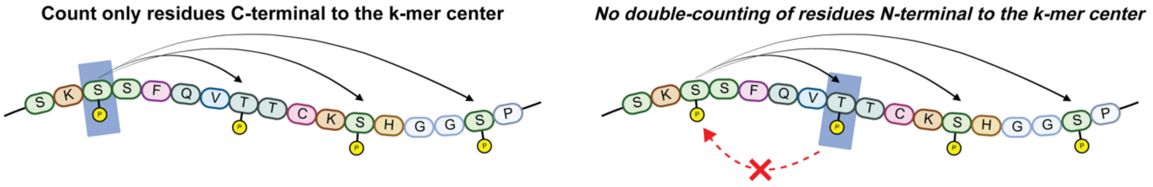

S3.2. Frequency of nearby PTM sites in human-only datasets taken from MusiteDeep

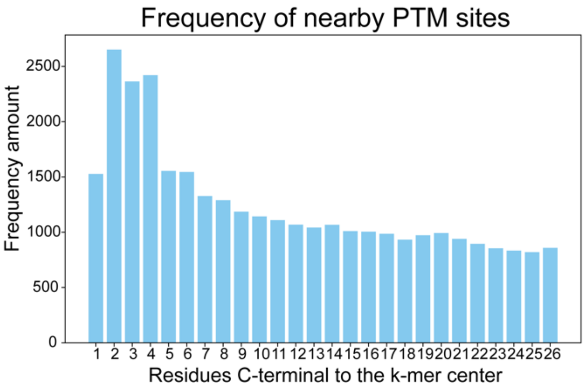

Phosphorylation (S,T)

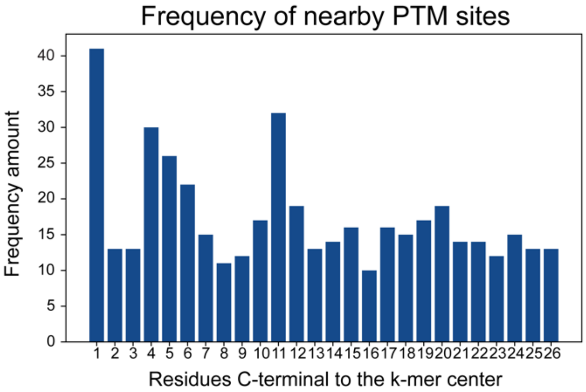

Phosphorylation (Y)

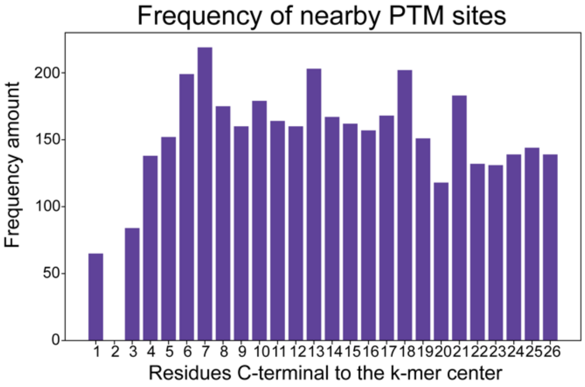

N-Glycosylation (N)

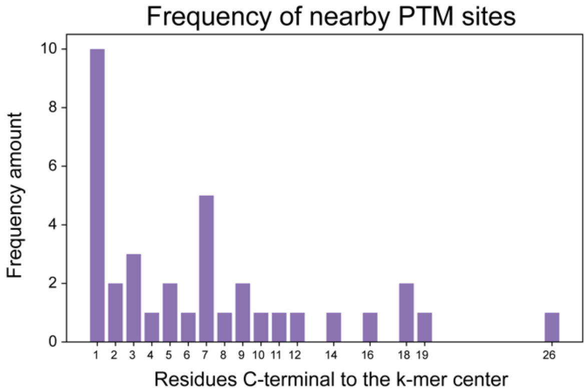

O-Glycosylation (S,T)

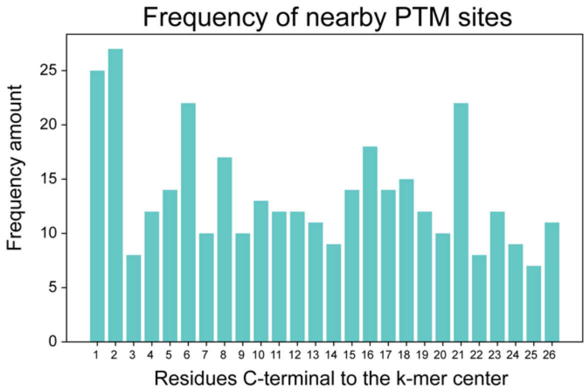

Ubiquitination (K)

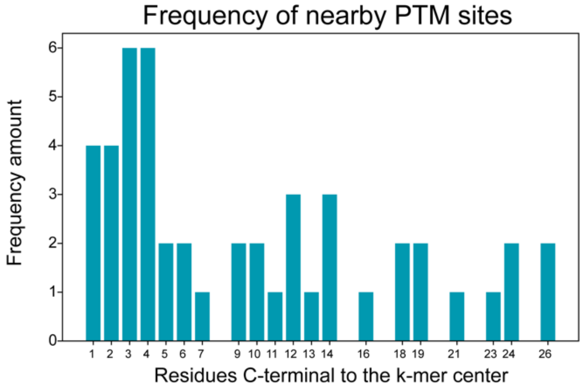

SUMOylation (K)

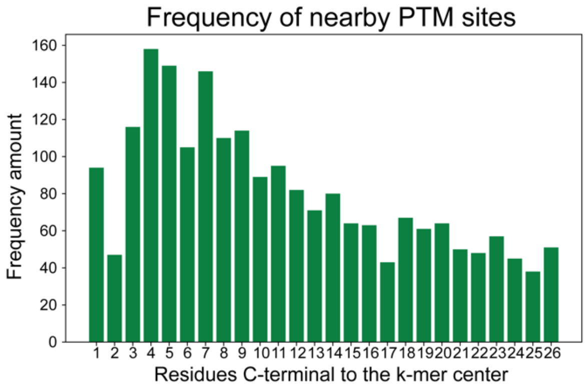

Acetylation (K)

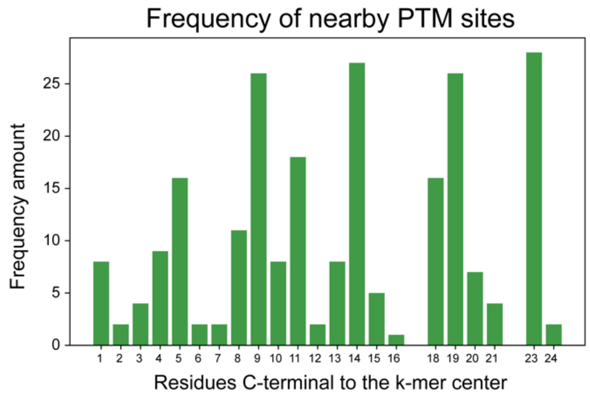

Methylation (K)

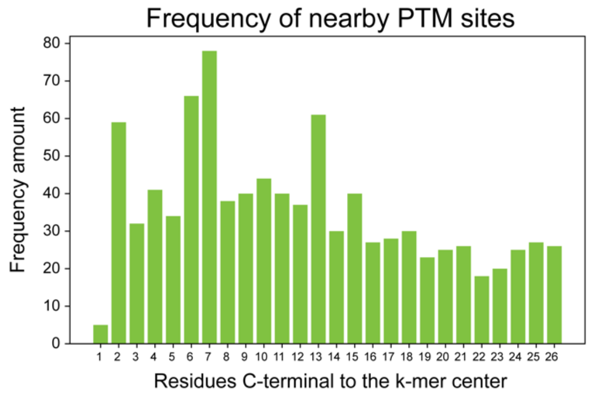

Methylation (R)

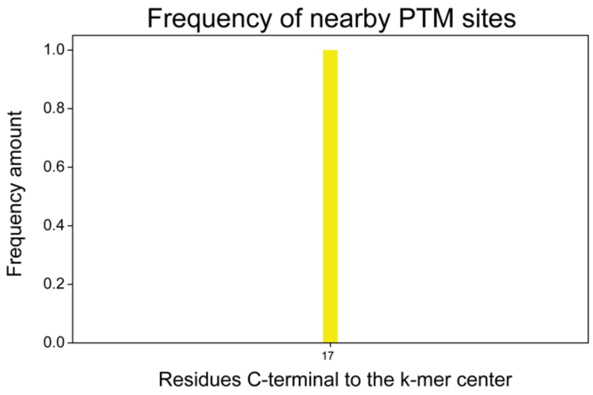

Pyroglutamylation (Q)

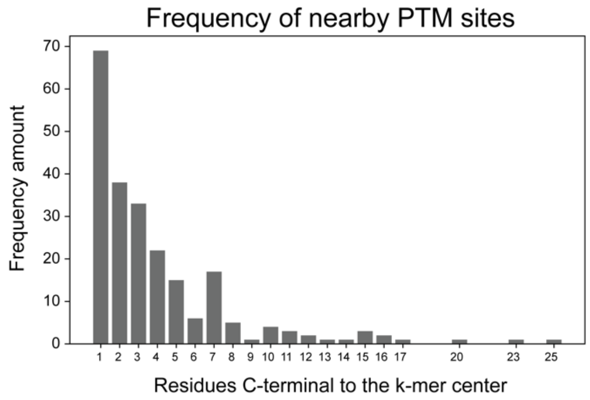

Palmitoylation (C)

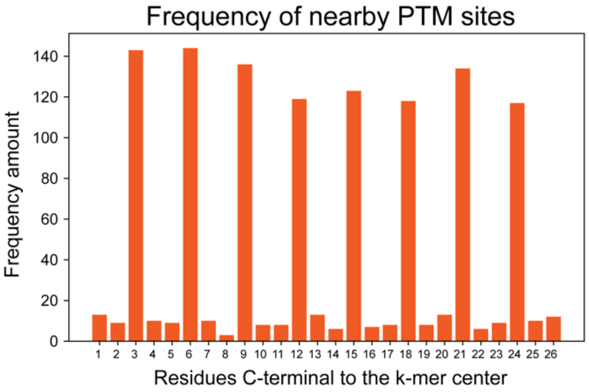

Hydroxylation (P)

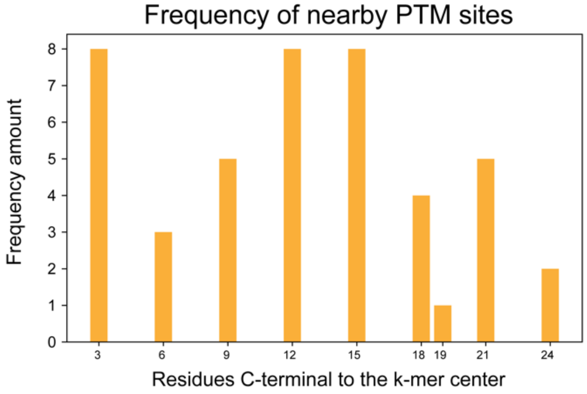

Hydroxylation (K)

S3.3. Frequency of Other Sites in All-Organisms from MusiteDeep

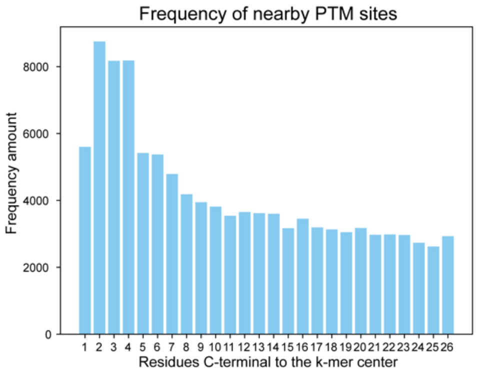

Phosphorylation (S,T)

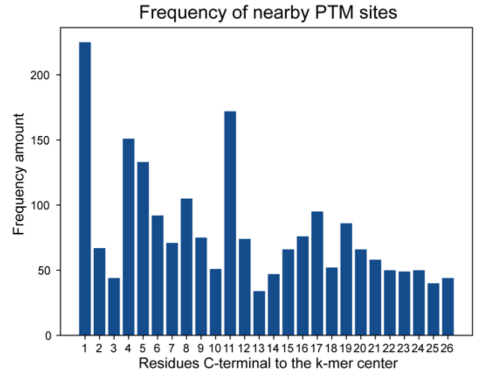

Phosphorylation (Y)

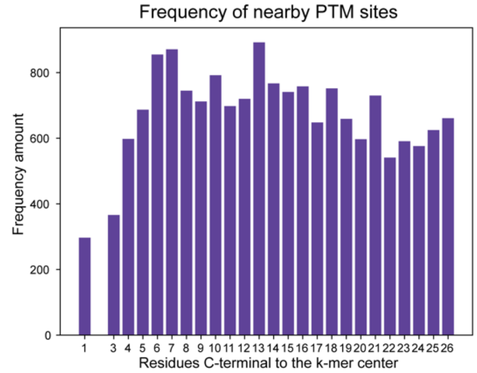

N-Glycosylation (N)

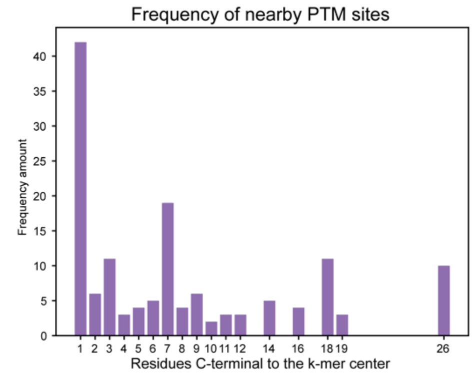

Glycosylation (O)

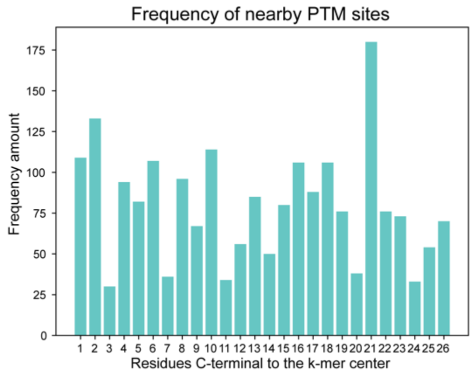

Ubiquitination (K)

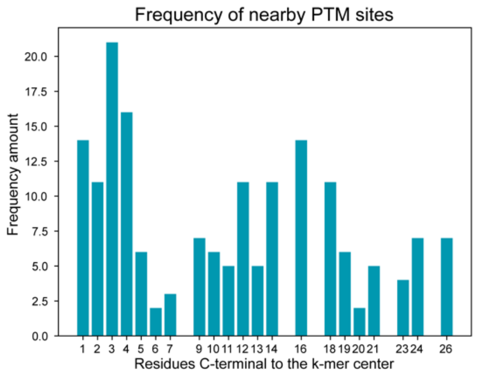

SUMOylation (K)

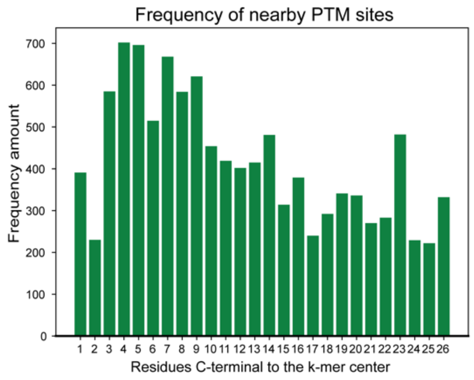

Acetylation (K)

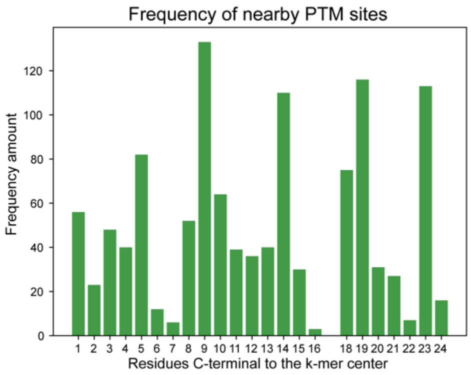

Methylation (K)

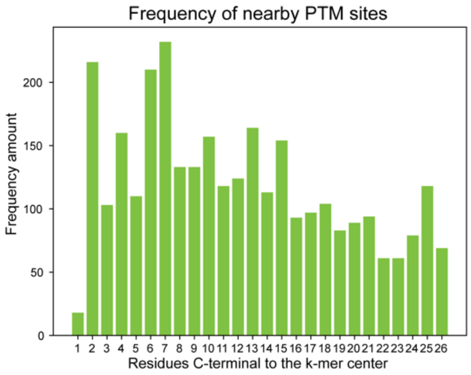

Methylation (R)

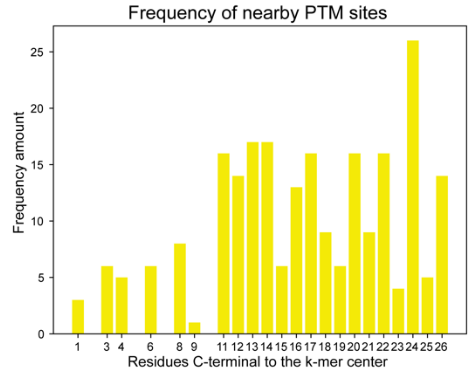

Pyroglutamylation (Q)

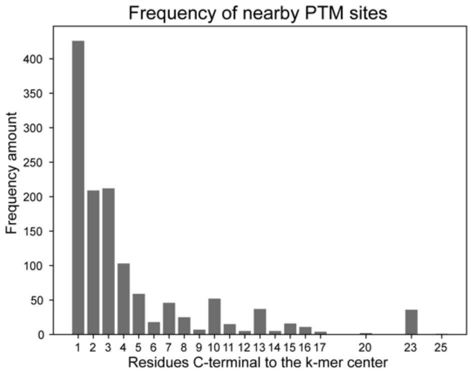

Palmitoylation (C)

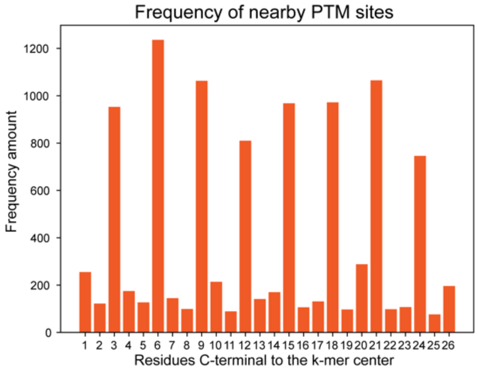

Hydroxylation (P)

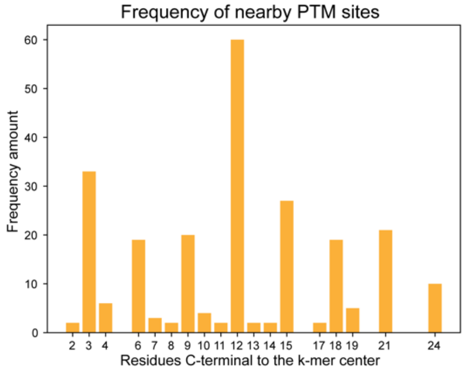

Hydroxylation (K)

S3.4. Frequency of Other O-Glycosylation Sites Taken from OGP and O-GlcNAc Site Atlas

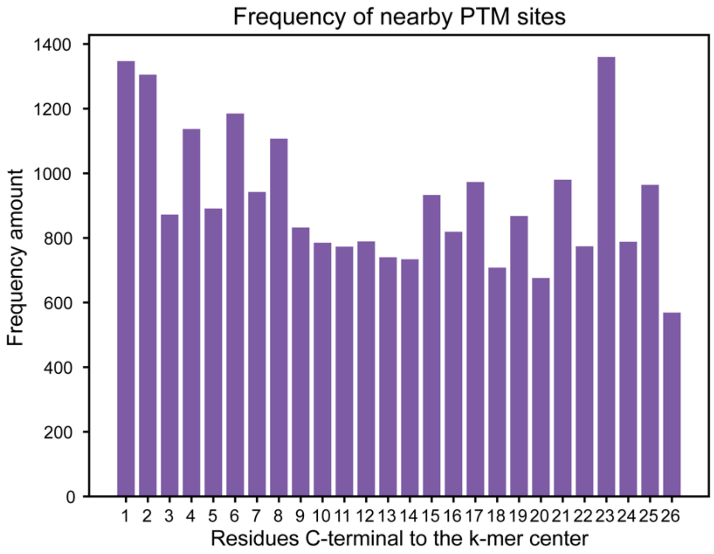

OGP O-glycosylation
Human-only proteins

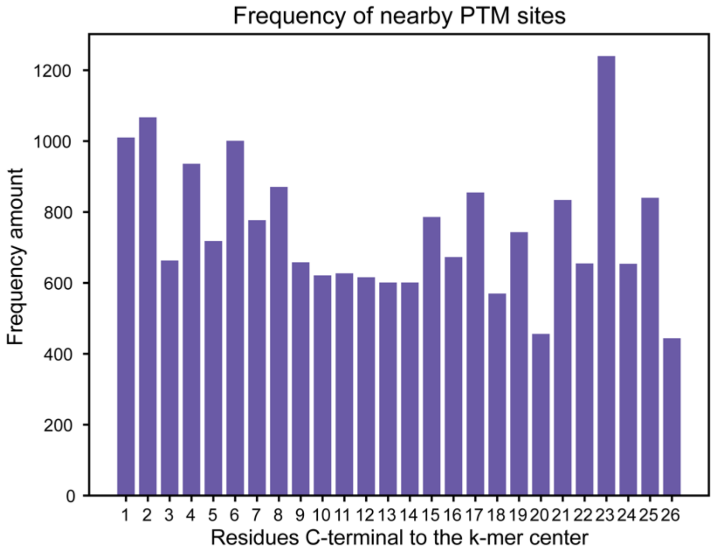

OGP GalNAc
Human-only proteins

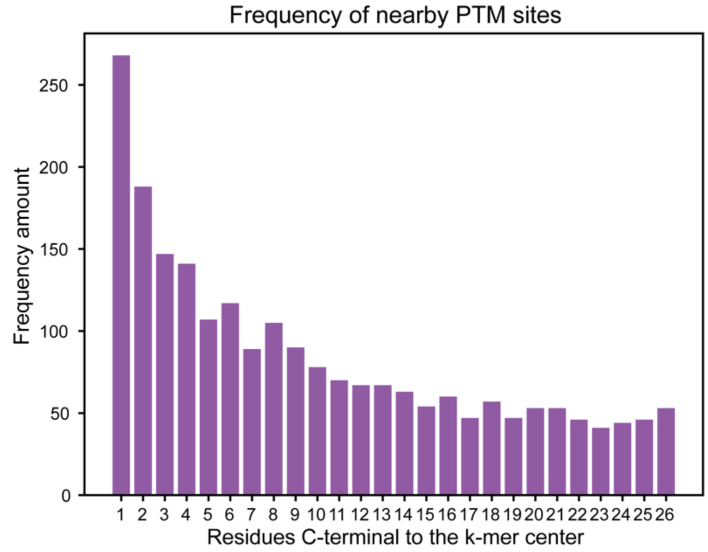

OGP HexNAc
Human-only proteins

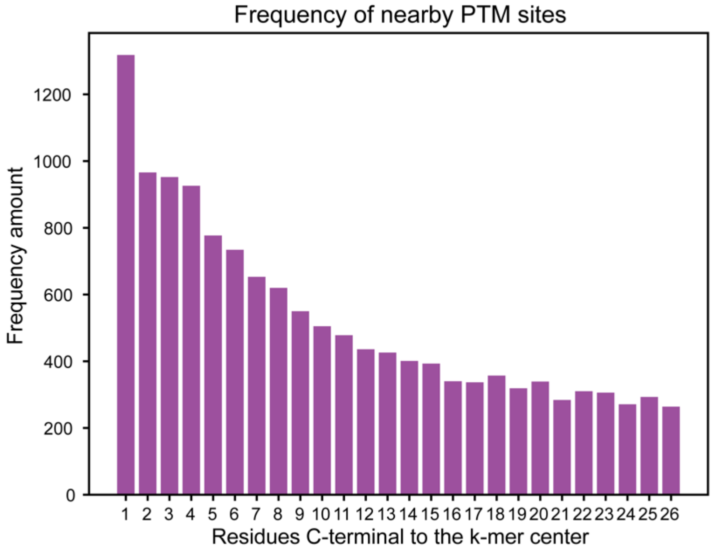

O-GlcNAc Site Atlas
Human-only proteins

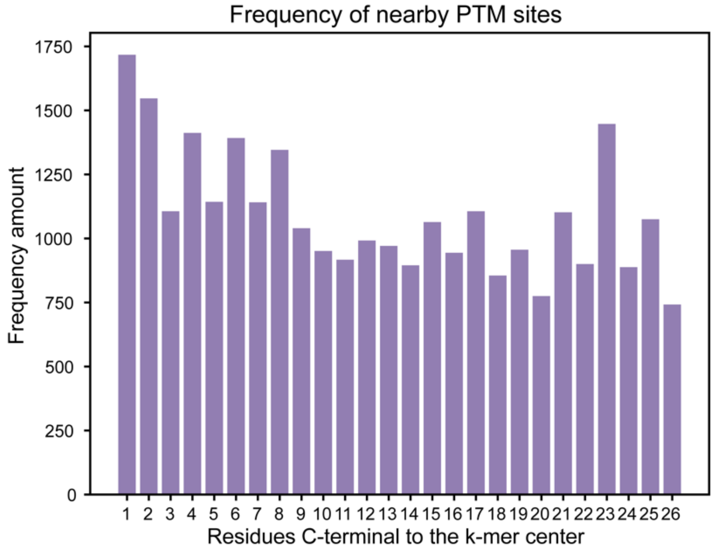

OGP O-glycosylation
All-organism proteins

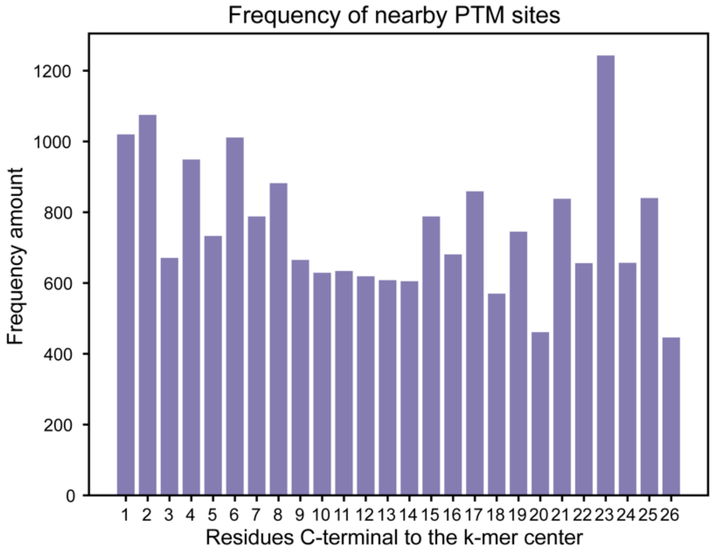

OGP GalNAc
All-organism proteins

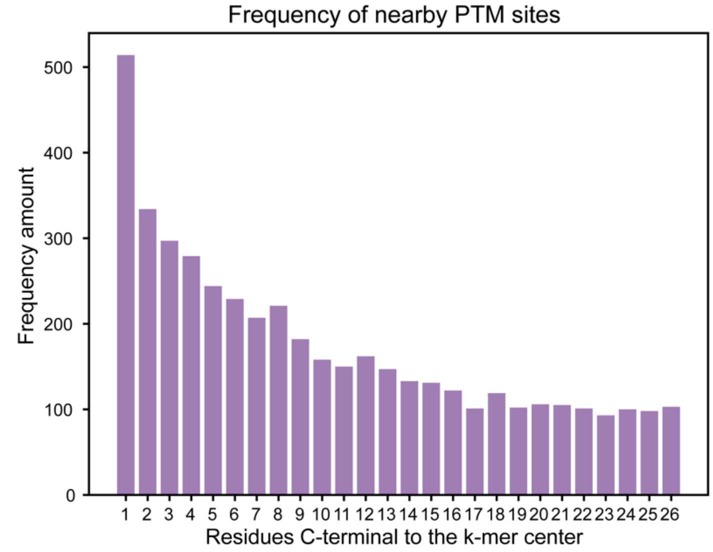

OGP HexNAc
All-organism proteins

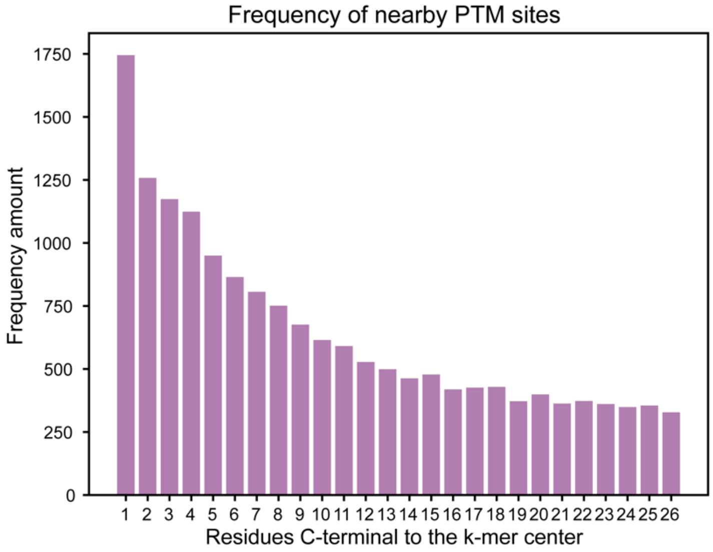

O-GlcNAc Site Atlas
All-organism proteins

S3.5. Frequency of Other N-Glycosylation Sites from LMNglyPred Datasets

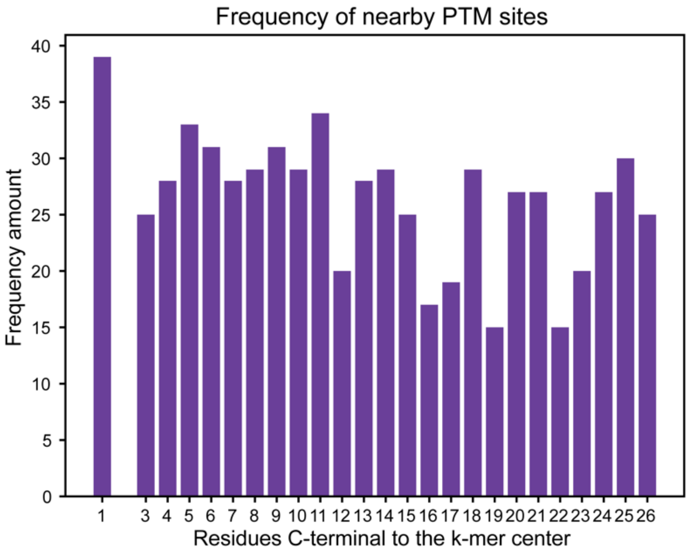

N-Glycosite Atlas dataset

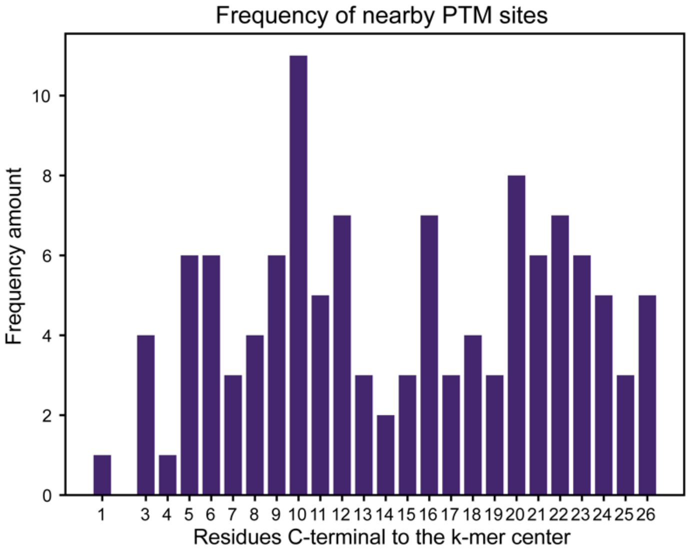

NGlyDE dataset

S3.6. Frequency of Other Phosphorylation (S,T) Sites from Sugiyama Kinase Datasets

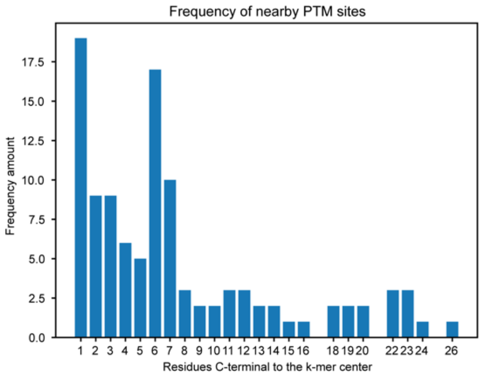

AurA

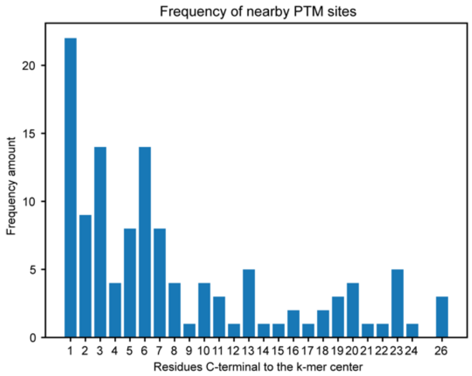

AurC

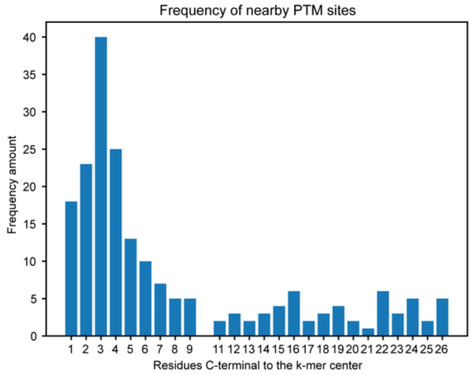

CaMK2δ

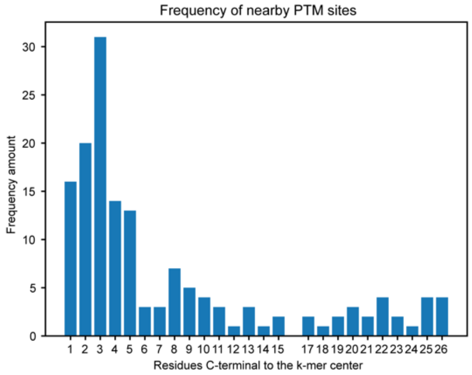

CaMK2γ

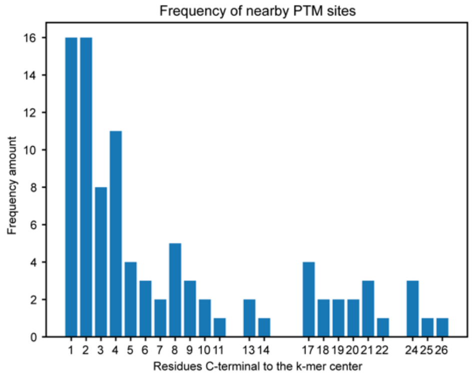

CDC2/CycB1

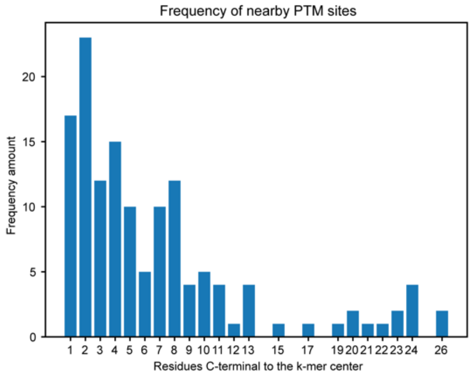

CDK5/p25

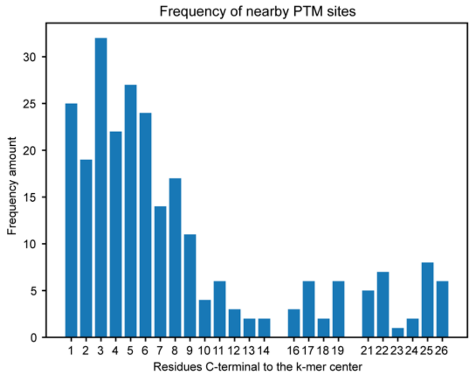

CHK1

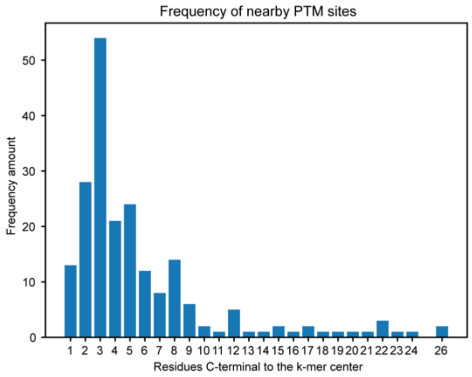

CK1δ

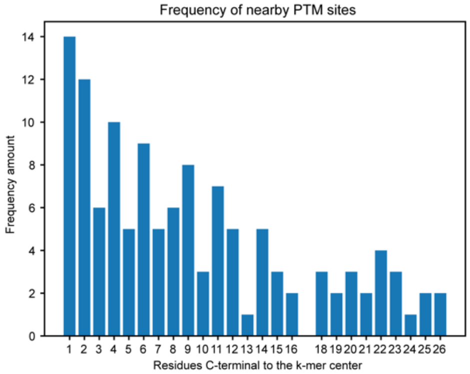

DCLK2

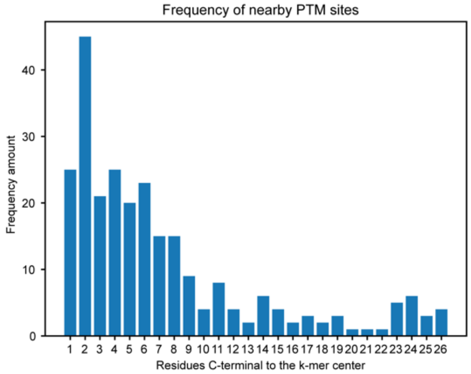

DYRK2

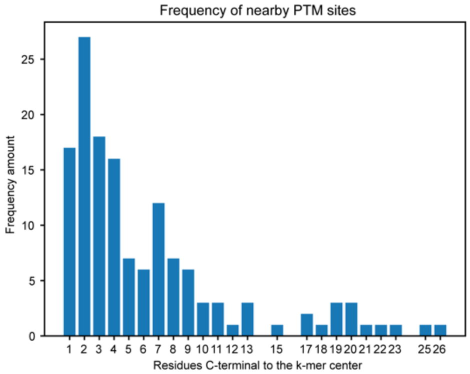

DYRK3

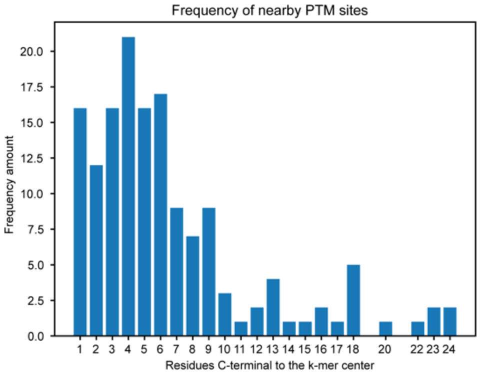

Erk1

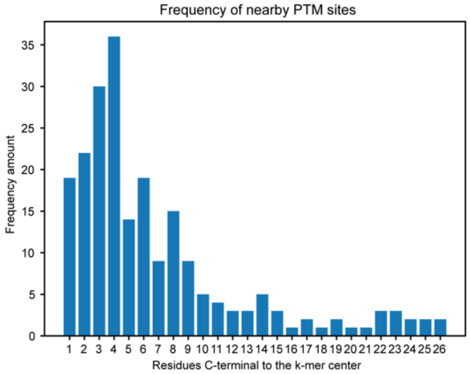

Erk2

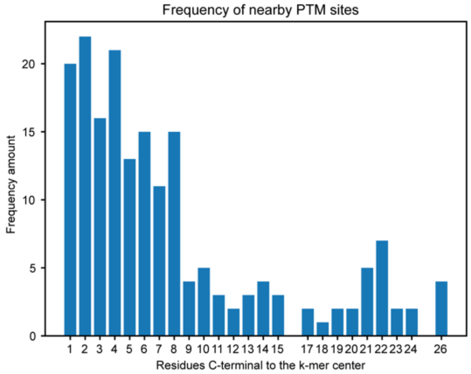

Erk5

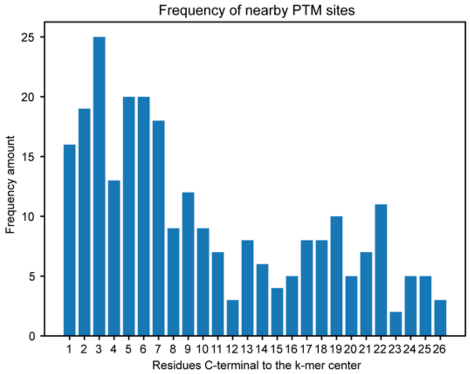

GCK

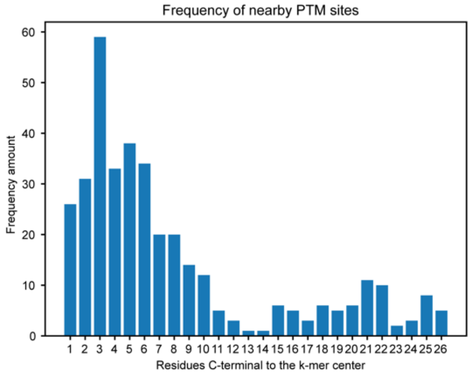

HGK

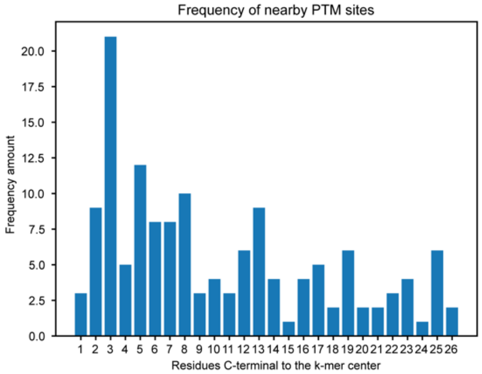

IKKα

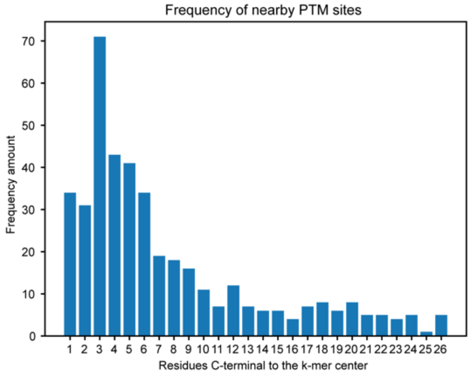

IKKβ

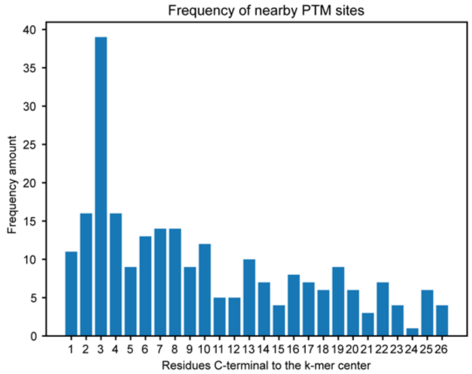

IKKε

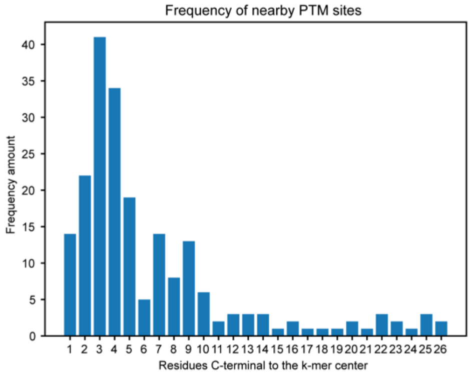

JNK1

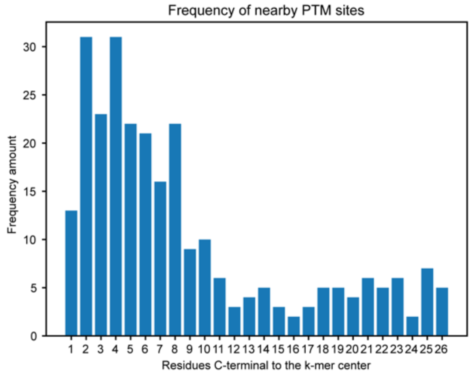

KHS1

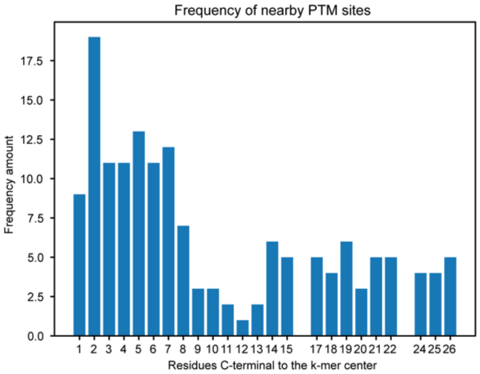

MAP3K1

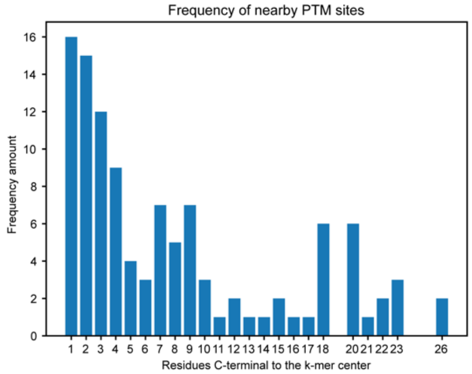

MAPKAPK3

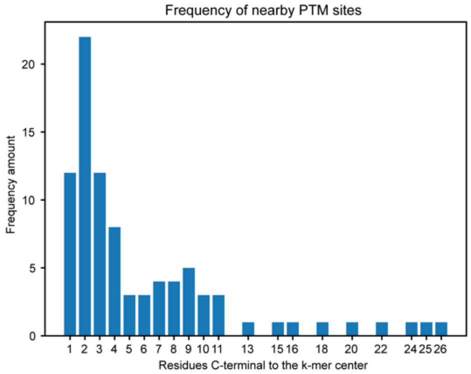

MAPKAPK5

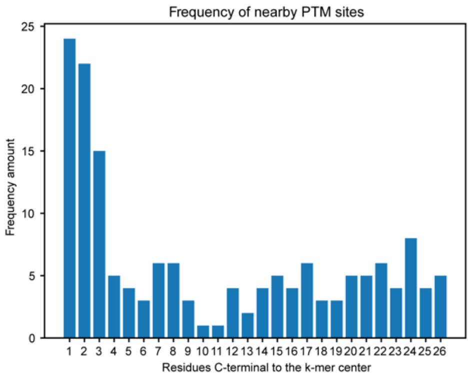

MARK2

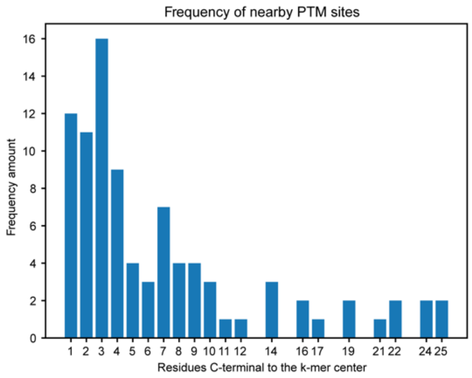

MARK4

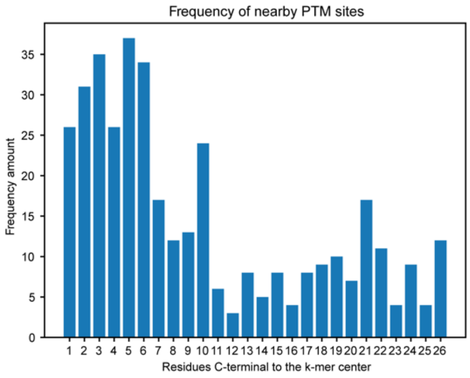

MINK

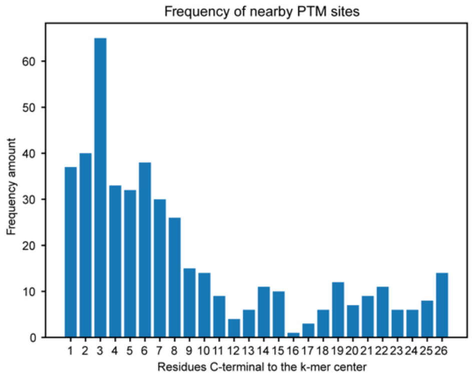

MLK1

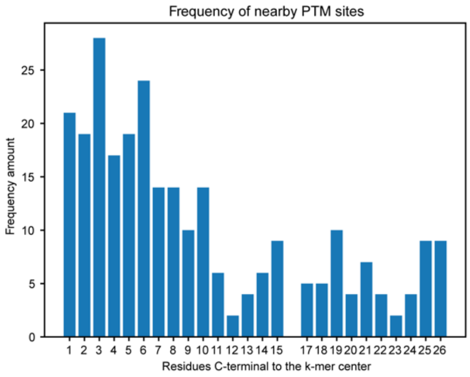

MLK2

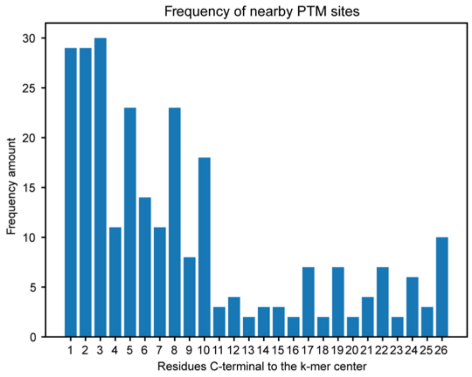

MLK3

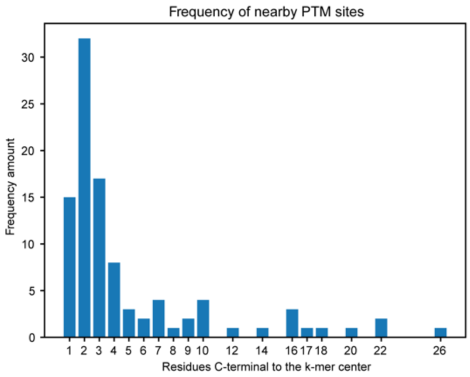

MSK1

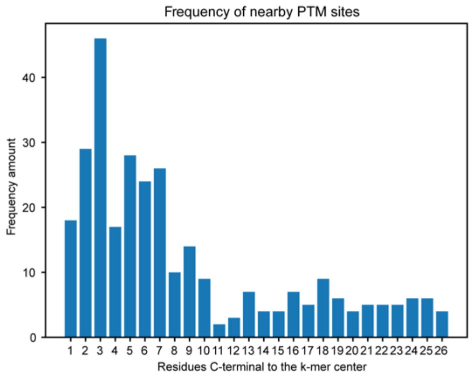

MST1

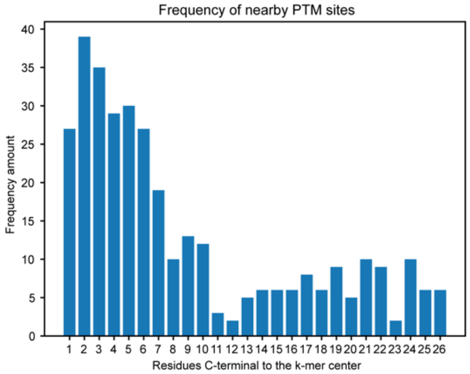

MST2

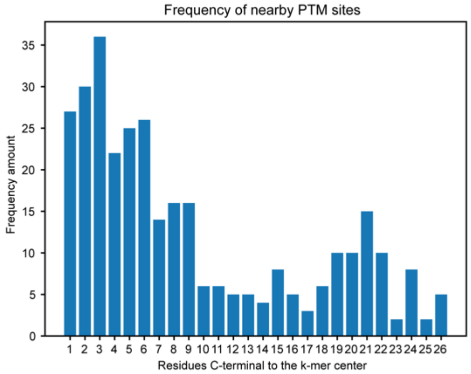

MST3

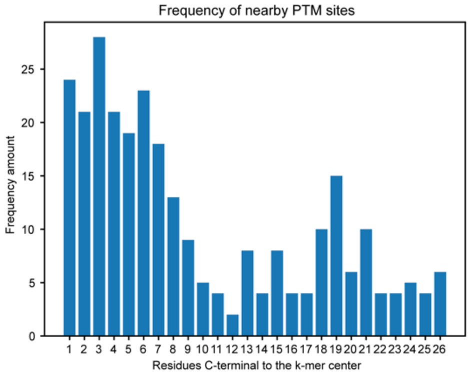

MST4

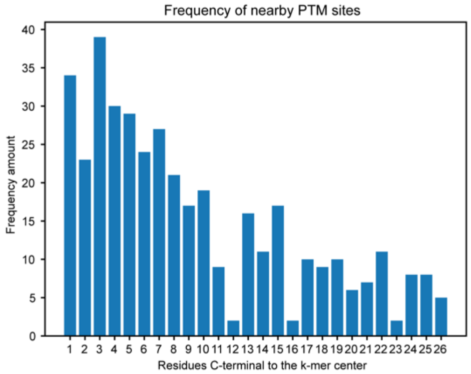

NEK1

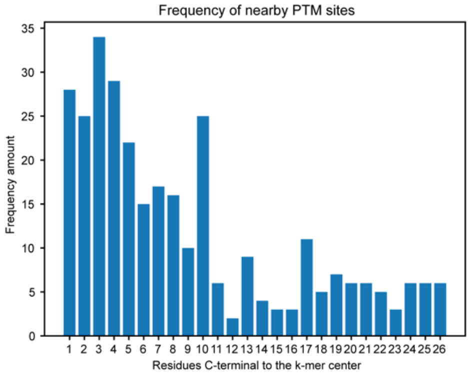

NEK2

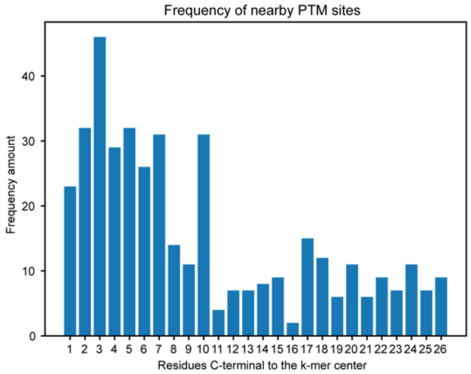

NEK4

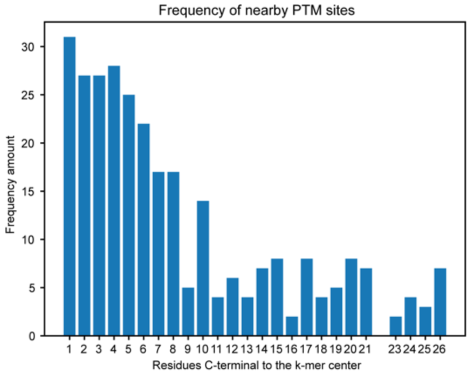

NEK6

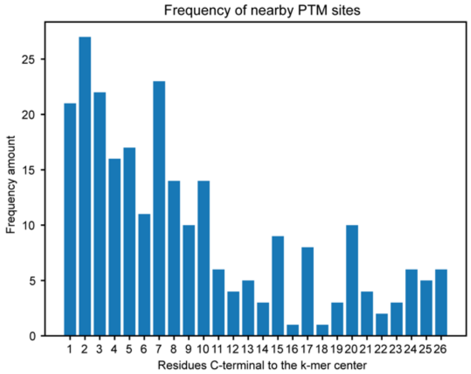

NEK7

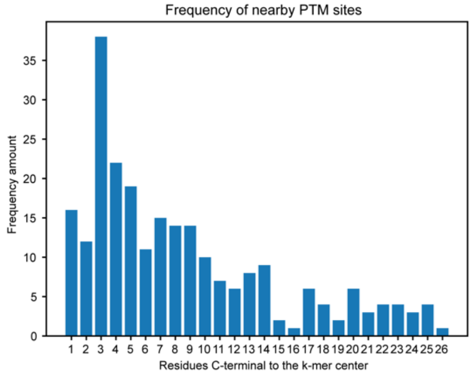

NEK9

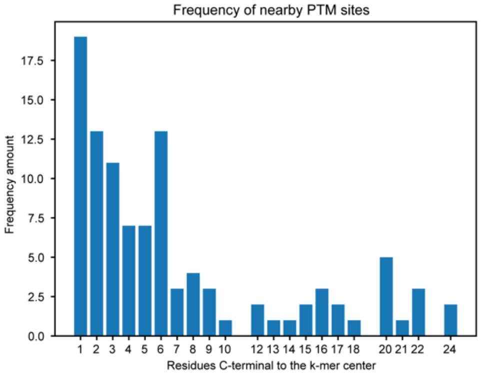

NIM1

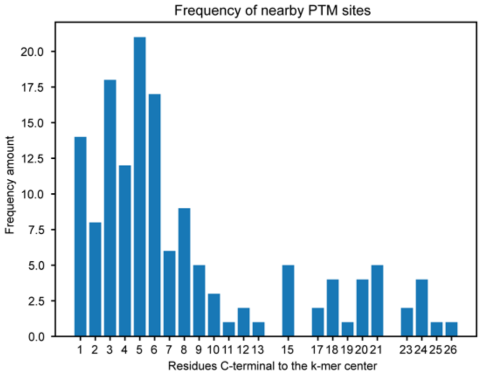

NLK

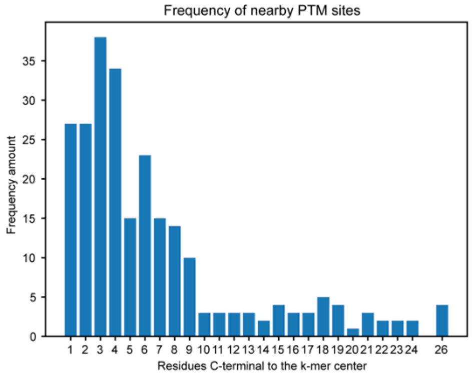

p38α

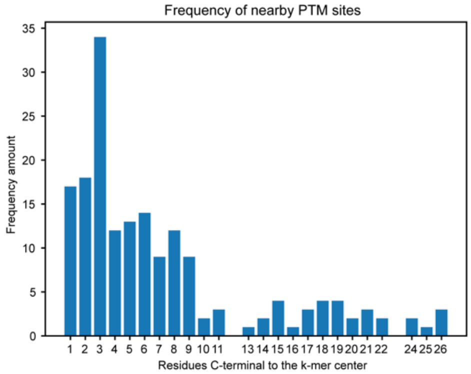

p38β

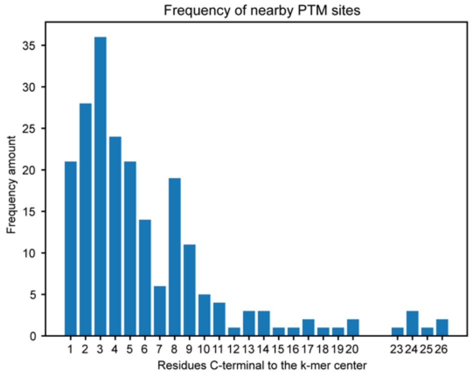

p38δ

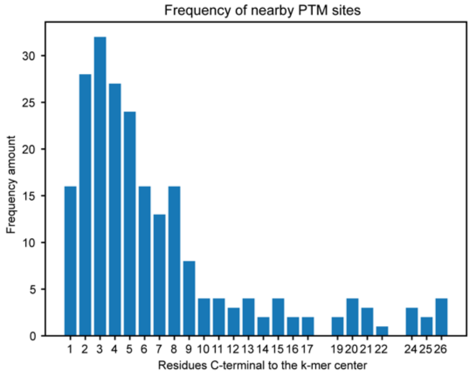

p38γ

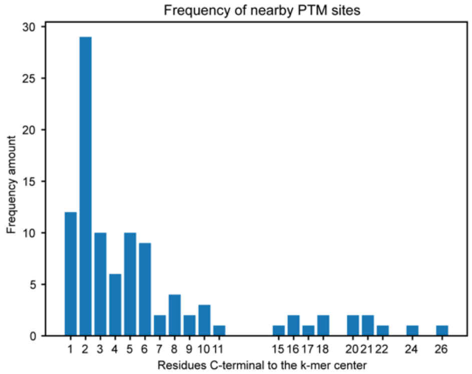

p70S6K

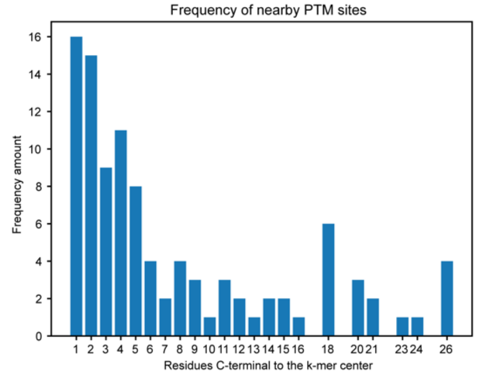

PAK1

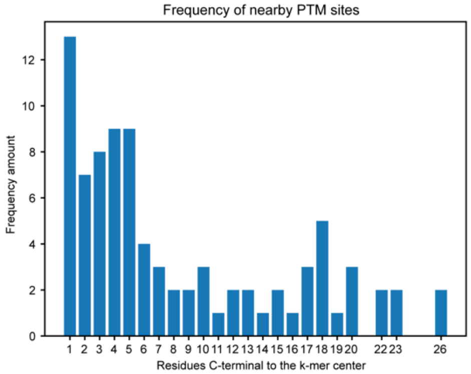

PAK2

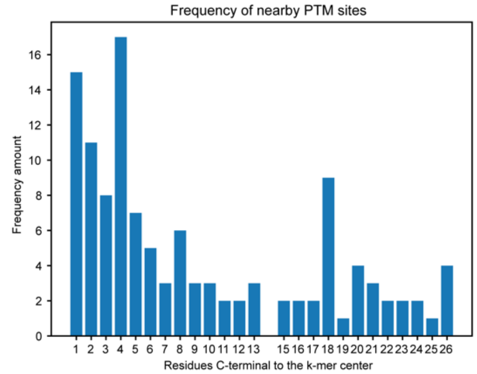

PAK3

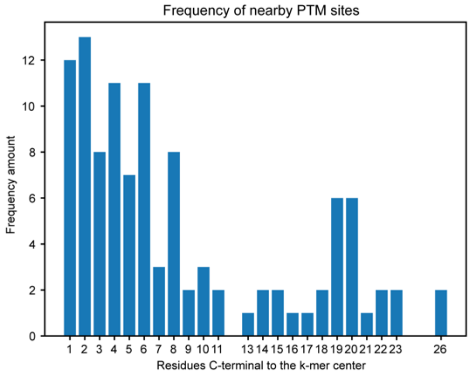

PHKG2

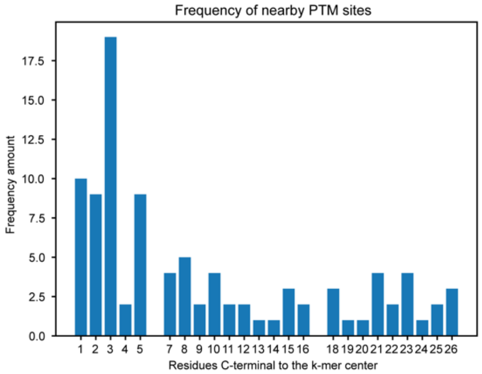

PIM1

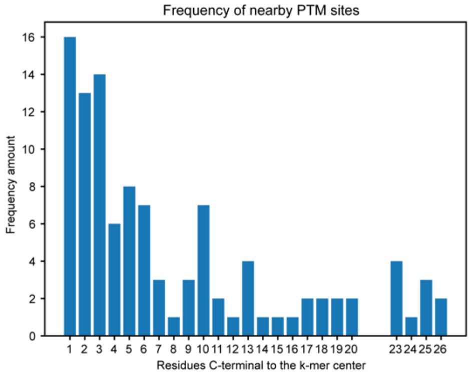

PIM3

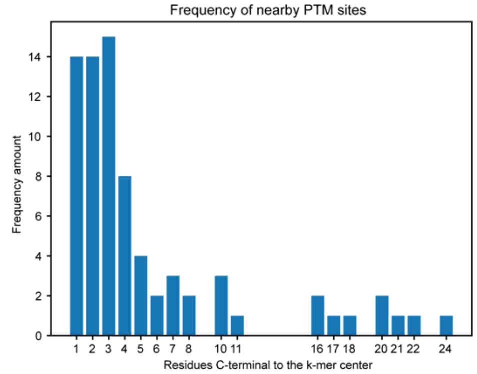

PKA Cα

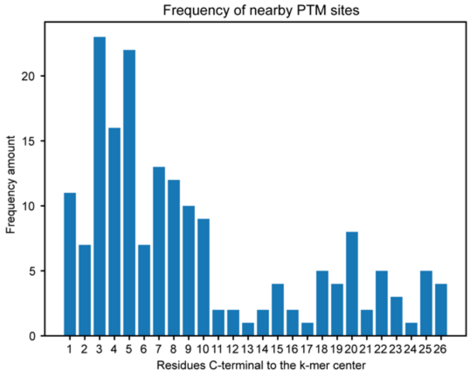

PLK1

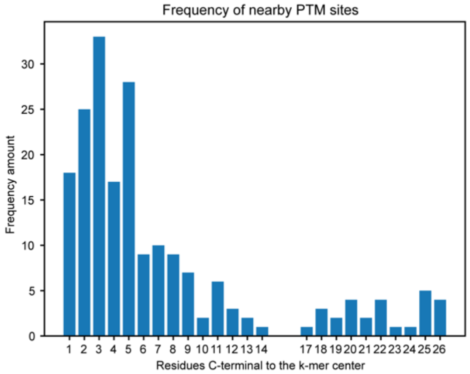

PLK3

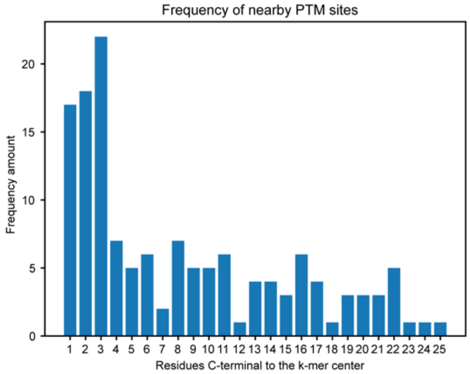

QIK

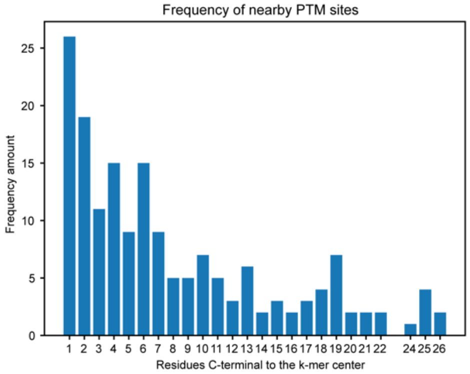

RSK3

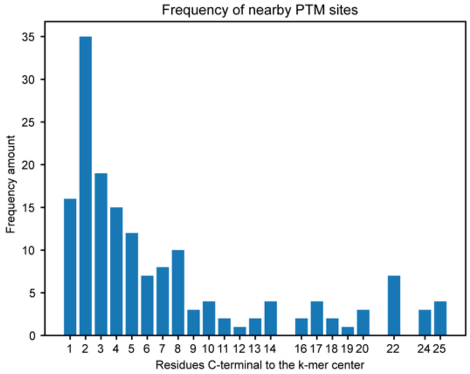

S9K3

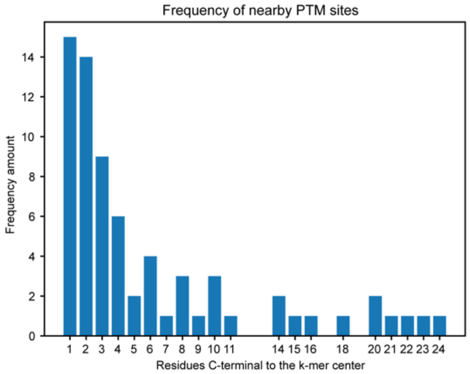

SIK

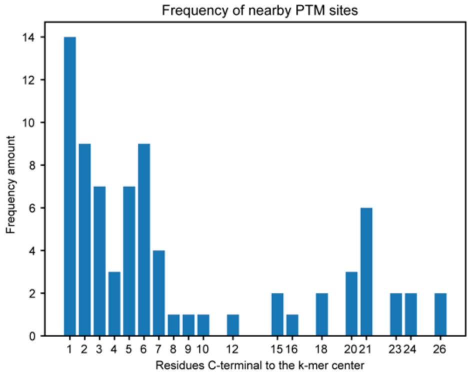

TAK1

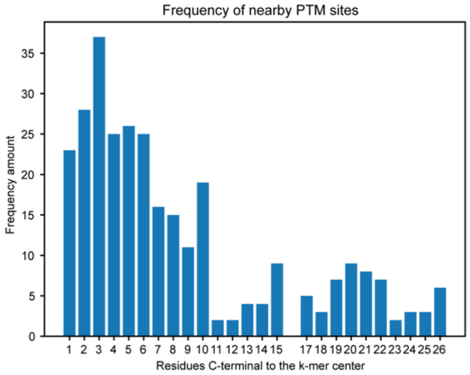

TAO2

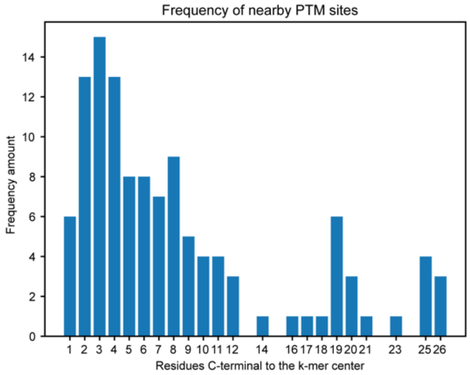

TLK1

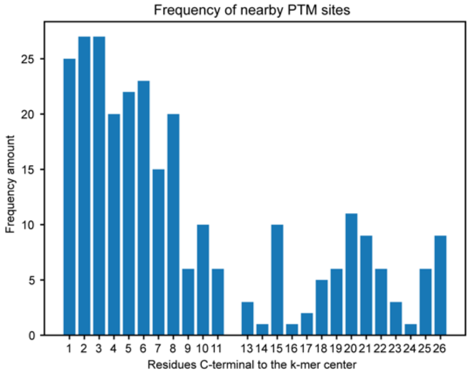

TNIK

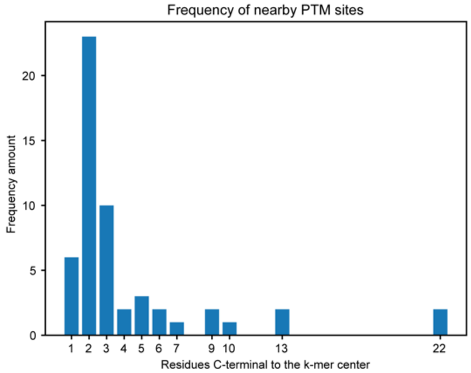

TSSK1

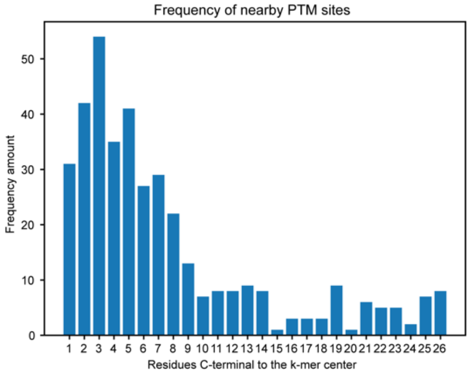

TTBK1

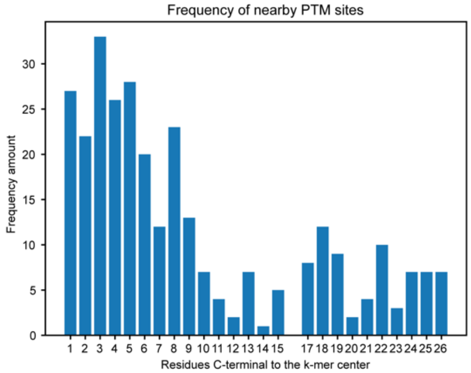

YSK1

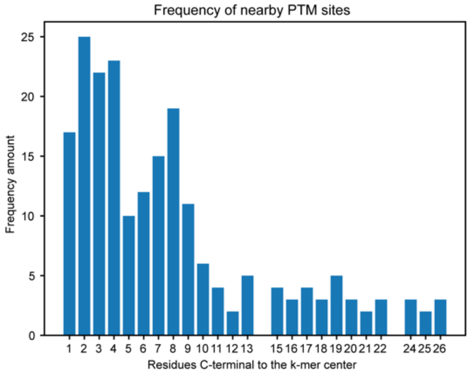

ZAK

S4. Tuning of Model Parameters

S4.1. Determination of Optimal "k" for k-mers

To ensure the best possible performance for our model, we tuned deep learning hyperparameters such as the learning rate, batch size, and epochs using the human phosphorylation (S,T) dataset. We also tested different k-mer lengths, which resulted in an optimal value of k = 53 that results in a high accuracy and reasonable size for running in models.

k-mer length	Accuracy
21	0.8430
31	0.8522
41	0.8583
51	0.8575
53	0.8624
55	0.8587
61	0.8610
71	0.8654

S4.2. Testing of Other Model Architectures

Different deep learning model architectures were tested for performance and run time. We chose the phosphorylation (S,T), N-glycosylation, and lysine acetylation models for testing because of the large datasets available, which offer variety towards frequencies of the same PTM.

One-Hot versus Embedding CNN

In designing our model, we explored two encoding methods for our CNN model: one-hot encoding and word embedding. One-hot encoding represents each letter as a zero vector with a length equal to the model's alphabet, placing a one at the index corresponding to the specific letter. This approach treats each letter as independent, discrete information, which results in the loss of information about the similarities between residues. To capture these similarities, we tried word embedding. Here, each residue was assigned an integer index, which was then input into an embedding layer that converts the index into a dense vector of floating-point values. These vectors are learned by the model during training. When comparing these two encoding methods across phosphorylation (S,T), N-glycosylation, and lysine acetylation models, word embedding consistently outperformed one-hot encoding.

	Word Embedding		One Hot Encoding	
**Model (Human-only)**	**+ PTMs**	**− PTMs**	**+ PTMs**	**− PTMs**
Phosphorylation (S,T)	**0.9301**	0.8812	0.9248	0.8703
N-Glycosylation (N)	**0.9807**	0.9732	0.9748	0.9705
Acetylation (K)	**0.8570**	0.7637	0.8192	0.7337

To ensure that our word embedding encoding captures relevant information, we performed PCA analysis on the embedding from our Phosphorylation (S,T) human model and saw that similar residues tended to be closer in vector space with phosphoserine and phospothreonine mapping very differently as shown below.

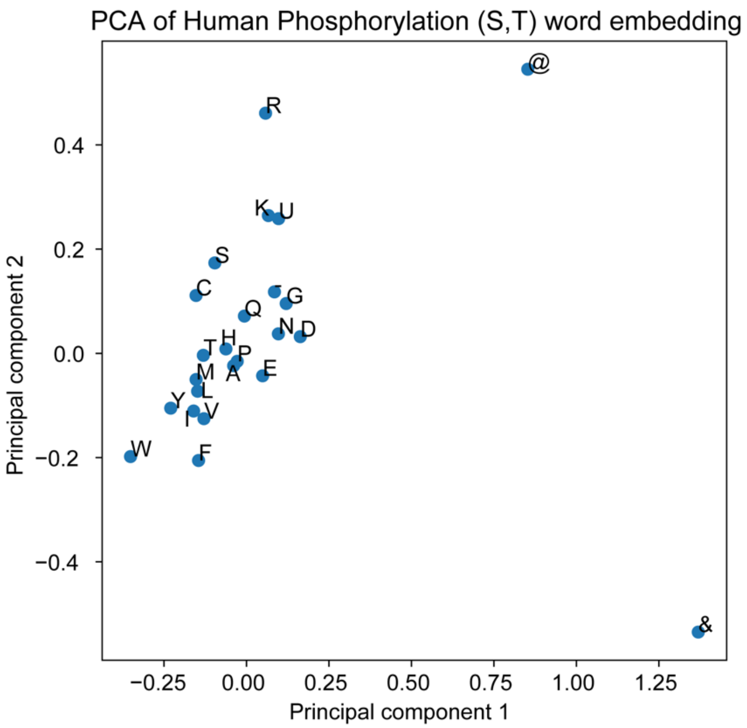

LSTM versus Attention

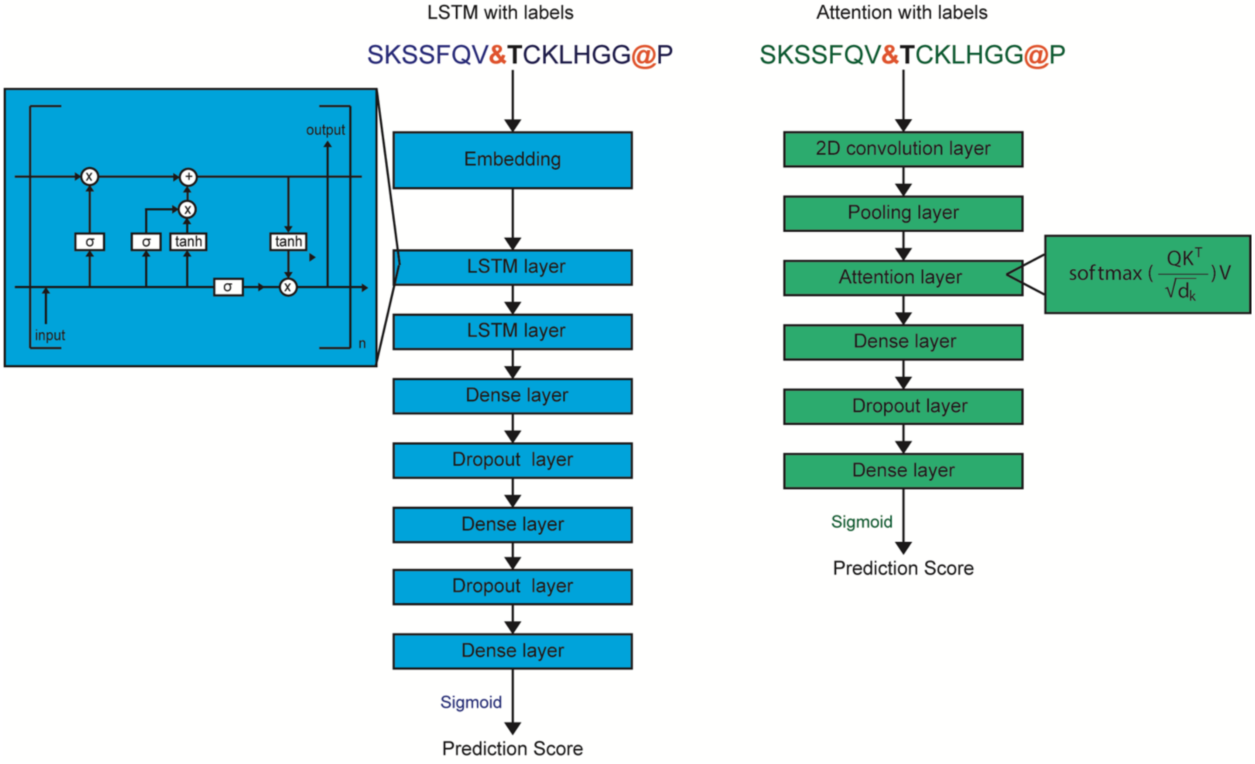

We also tested different model architectures, including those with a long short-term memory (LSTM) layer or a dot-product attention layer. LSTMs are a type of recurrent neural network (RNN) that can capture long-term dependencies by using a memory cell to maintain information over time. In contrast, attention mechanisms enable RNNs to shift from representing an input sequence as a single content vector to

generating a content vector for each input value, weighted by its relevance to the current state. When comparing these two models with a CNN across phosphorylation (S,T), N-glycosylation, and lysine acetylation models, LSTM consistently outperformed Attention.

	LSTM		Attention		CNN	
**Model (Human-only)**	**+ PTMs**	**− PTMs**	**+ PTMs**	**− PTMs**	**+ PTMs**	**− PTMs**
Phosphorylation (S,T)	0.9282[a]	0.8854[a]	0.9139[a]	0.8564[a]	**0.9301**	0.8812
N-Glycosylation (N)	**0.9872**[a]	0.9820[a]	0.9637[a]	0.9605[a]	0.9807	0.9732
Acetylation (K)	**0.8676**[a]	0.7891[a]	0.7351[a]	0.6324[a]	0.8570	0.7637

[a]A significant difference ($P < 0.05$) exists between the model architecture and CNN embedding model.

Overall, we found the best to be the embedding CNN model, which not only produces the highest AUC for phosphorylation of serine and threonine but is also a relatively simple model that runs on the order of minutes rather than hours as the LSTM and Attention models tend to do.

S4.3. Hyperparameter Tuning

Further tuning was done with the hyperparameters learning rate, dropout rate, L2 rate, batch size, and kernel size using the Human Phosphorylation (S,T) dataset. We tried learning rates of 0.1, 0.01, 1E−03, and 1E−04. We tried dropout rates of 0.9, 0.5, 0.2, and 0.1. We tried L2 rates of 1E−03, 1E−06, and 0. We tried batch sizes of 10, 30, 50, and 100. We tried kernel sizes of 3×3 and 5×5. We tested all possible combinations of these hyperparameters with a small k-mer size of 35 to have a reasonable run time. Our best results were a learning rate of 1E−03, a dropout rate of 0.1, and L2 rate of 1E−06, a batch size of 50, and a kernel size of 3×3, allowing our accuracy to improve from 79% to 85%. In our actual model, we changed the learning rate to 1E−04 and the batch size to 256, which had similar results and worked well for every PTM.

S4.4. Additional Model Architecture Results

PTM	Model			AUC	AUPRC	Accuracy	Recall	Precision	MCC	F1	Specificity
**Phosphorylation (S,T)**	LSTM	No labels	Avg	0.8854	0.884	0.8048	0.8048	0.8048	0.6097	0.8048	0.8048
			STD	0.0034	0.0034	0.0045	0.0045	0.0045	0.009	0.0045	0.0045
		Labels	Avg	0.9282	0.928	0.8519	0.8519	0.8519	0.7039	0.8519	0.8519
			STD	0.0019	0.002	0.0038	0.0038	0.0038	0.0076	0.0038	0.0038
			P value	5.14E−11	5.73E−11	4.79E−10	4.79E−10	4.79E−10	4.68E−10	4.79E−10	4.79E−10
**Phosphorylation (S,T)**	Attention	No labels	Avg	0.8564	0.8476	0.7747	0.7839	0.7721	0.5495	0.7778	0.7652
			STD	0.0042	0.0049	0.0048	0.0163	0.0078	0.0098	0.0055	0.0159
		Labels	Avg	0.9139	0.9166	0.8388	0.8299	0.8468	0.6778	0.8382	0.8477
			STD	0.0031	0.0027	0.004	0.0127	0.0042	0.0079	0.0057	0.0074
			P value	2.08E−14	6.95E−14	9.56E−12	5.42E−06	3.26E−09	9.97E−12	1.52E−11	1.11E−07
**Phosphorylation (S,T)**	One-Hot CNN	No labels	Avg	0.8703	0.861	0.7897	0.798	0.7872	0.5795	0.7925	0.7813
			STD	0.0024	0.0041	0.0039	0.0124	0.0078	0.008	0.004	0.0112
		Labels	Avg	0.9248	0.9263	0.8511	0.8404	0.8606	0.7025	0.8503	0.862
			STD	0.0026	0.0018	0.0042	0.0095	0.008	0.0085	0.0043	0.0099
			P value	1.53E−13	3.26E−12	5.51E−12	2.24E−05	2.29E−08	8.39E−12	1.74E−11	2.56E−07
**N-Glycosylation (N)**	LSTM	No labels	Avg	0.982	0.9823	0.9416	0.9416	0.9416	0.8831	0.9416	0.9416
			STD	0.0023	0.0023	0.0043	0.0043	0.0043	0.0087	0.0043	0.0043
		Labels	Avg	0.9872	0.9874	0.9486	0.9486	0.9486	0.8972	0.9486	0.9486
			STD	0.0016	0.0016	0.0041	0.0041	0.0041	0.0082	0.0041	0.0041
			P value	1.85E−07	2.74E−07	6.34E−06	6.34E−06	6.34E−06	7.08E−06	6.34E−06	6.34E−06
**N-Glycosylation (N)**	Attention	No labels	Avg	0.9605	0.9396	0.9278	0.9791	0.888	0.8602	0.9313	0.8764
			STD	0.0044	0.0075	0.006	0.0046	0.0104	0.0111	0.0057	0.0122
		Labels	Avg	0.9637	0.9459	0.9282	0.977	0.8901	0.8606	0.9315	0.8795
			STD	0.0029	0.0072	0.004	0.004	0.0083	0.0071	0.0041	0.0085
			P value	1.06E−01	5.14E−02	8.61E−01	1.51E−01	6.08E−01	9.25E−01	9.26E−01	5.65E−01
**N-Glycosylation (N)**	One-Hot CNN	No labels	Avg	0.9705	0.9557	0.9419	0.9832	0.9083	0.887	0.9442	0.9007

			STD	0.0041	0.0088	0.0038	0.0038	0.0086	0.0068	0.0038	0.0091
		Labels	Avg	0.9748	0.9644	0.9435	0.9794	0.9138	0.8893	0.9454	0.9076
			STD	0.0039	0.0068	0.0033	0.0044	0.0079	0.006	0.0034	0.0084
			P value	8.65E−05	3.14E−04	2.77E−02	5.45E−02	6.63E−03	6.98E−02	5.56E−02	6.79E−03
**Acetylation (K)**	LSTM	No labels	Avg	0.7891	0.7857	0.7204	0.7204	0.7204	0.4407	0.7204	0.7204
			STD	0.0181	0.019	0.0139	0.0139	0.0139	0.0279	0.0139	0.0139
		Labels	Avg	0.8676	0.8701	0.7743	0.7743	0.7743	0.5487	0.7743	0.7743
			STD	0.0112	0.0107	0.0122	0.0122	0.0122	0.0243	0.0122	0.0122
			P value	4.93E−09	6.08E−09	1.27E−07	1.27E−07	1.27E−07	1.26E−07	1.27E−07	1.27E−07
**Acetylation (K)**	Attention	No labels	Avg	0.6324	0.678	0.5923	0.6235	0.5469	0.1863	0.5673	0.5624
			STD	0.0777	0.0534	0.0621	0.2624	0.1917	0.125	0.191	0.2997
		Labels	Avg	0.7351	0.7457	0.6688	0.6346	0.68	0.3386	0.6556	0.7026
			STD	0.0206	0.0286	0.0156	0.0437	0.0184	0.0306	0.0241	0.0292
			P value	4.1E−03	1.58E−03	5.54E−03	8.93E−01	6.15E−02	5.94E−03	1.89E−01	1.72E−01
**Acetylation (K)**	One-Hot CNN	No labels	Avg	0.7337	0.7295	0.6688	0.678	0.6652	0.338	0.671	0.6596
			STD	0.0203	0.0196	0.0144	0.0253	0.0253	0.0292	0.0154	0.0359
		Labels	Avg	0.8192	0.8355	0.7389	0.7246	0.7457	0.4779	0.7345	0.7524
			STD	0.013	0.0076	0.0139	0.0264	0.0177	0.0267	0.0115	0.0358
			P value	6.69E−09	1.64E−08	4.44E−08	1.11E−03	4.49E−06	3.52E−08	1.71E−07	5.15E−05

S5. Additional Model Results

S5.1. Model results in human-only datasets taken from MusiteDeep

PTM			AUC	AUPRC	Accuracy	Recall	Precision	MCC	F1	Specificity
**Phosphorylation (S,T)**	No labels	Avg	0.8790	0.8709	0.7963	0.7897	0.8029	0.5932	0.7959	0.8031
		STD	0.0062	0.0069	0.0074	0.0246	0.0133	0.0147	0.0101	0.0193
	Labels	Avg	0.9301	0.9304	0.8575	0.8505	0.8646	0.7154	0.8573	0.8648
		STD	0.0015	0.0018	0.0028	0.0121	0.0102	0.0056	0.0031	0.0129
		P value	3.51E−10	1.75E−10	5.65E−11	1.51E−05	3.87E−09	4.57E−11	3.56E−09	6.59E−07
**Phosphorylation (Y)**	No labels	Avg	0.8030	0.7888	0.7250	0.7116	0.7331	0.4507	0.7207	0.7377
		STD	0.0150	0.0320	0.0153	0.0460	0.0339	0.0317	0.0236	0.0499
	Labels	Avg	0.8368	0.8533	0.7538	0.7243	0.7706	0.5100	0.7453	0.7846
		STD	0.0187	0.0275	0.0191	0.0481	0.0398	0.0375	0.0306	0.0301
		P value	0.0006	0.0002	0.0026	0.5725	0.0452	0.0020	0.0728	0.0292
**N-Glycosylation (N)**	No labels	Avg	0.9681	0.9453	0.9446	0.9856	0.9103	0.8922	0.9464	0.9040
		STD	0.0043	0.0088	0.0039	0.0040	0.0073	0.0074	0.0039	0.0075
	Labels	Avg	0.9806	0.9751	0.9480	0.9840	0.9173	0.8984	0.9495	0.9124
		STD	0.0028	0.0045	0.0038	0.0037	0.0075	0.0071	0.0039	0.0076
		P value	2.46E−06	4.45E−07	7.54E−02	3.94E−01	6.00E−02	8.75E−02	1.12E−01	3.15E−02
**O-Glycosylation (S,T)**	No labels	Avg	0.7035	0.7634	0.6591	0.8471	0.6527	0.2695	0.7309	0.4095
		STD	0.1014	0.0646	0.0717	0.1138	0.0706	0.1911	0.0553	0.2252
	Labels	Avg	0.7220	0.7662	0.6814	0.8672	0.6751	0.2851	0.7487	0.4081
		STD	0.1323	0.1058	0.1008	0.1472	0.0918	0.2709	0.0840	0.3170
		P value	0.7435	0.9457	0.5957	0.7491	0.5691	0.8898	0.6040	0.9915
**Ubiquitination (K)**	No labels	Avg	0.6513	0.6147	0.6134	0.5674	0.5738	0.2185	0.5535	0.6439
		STD	0.0473	0.0704	0.0412	0.1744	0.0632	0.0899	0.1158	0.1352
	Labels	Avg	0.8258	0.8356	0.7290	0.7215	0.7130	0.4691	0.7061	0.7383
		STD	0.0347	0.0288	0.0532	0.0980	0.0906	0.0855	0.0390	0.1419
		P value	1.00E−07	1.63E−06	8.13E−05	3.65E−02	1.63E−03	1.01E−05	3.21E−03	1.66E−01

**SUMOylation (K)**	No labels	Avg	0.8522	0.8619	0.7843	0.7664	0.7942	0.5763	0.7748	0.8057
		STD	0.0510	0.0557	0.0271	0.0829	0.0623	0.0519	0.0344	0.0768
	Labels	Avg	0.8688	0.8756	0.7863	0.7774	0.7873	0.5773	0.7775	0.7944
		STD	0.0616	0.0626	0.0484	0.1048	0.0606	0.0969	0.0595	0.0708
		P value	0.5418	0.6303	0.9121	0.8071	0.8141	0.9801	0.9058	0.7487
**Acetylation (K)**	No labels	Avg	0.7320	0.7069	0.6717	0.6286	0.6802	0.3462	0.6512	0.7151
		STD	0.0248	0.0281	0.0200	0.0540	0.0360	0.0406	0.0263	0.0453
	Labels	Avg	0.8530	0.8675	0.7708	0.7161	0.7967	0.5443	0.7532	0.8239
		STD	0.0102	0.0086	0.0115	0.0379	0.0214	0.0198	0.0164	0.0258
		P value	1.33E−08	6.48E−09	2.85E−09	1.06E−03	6.08E−07	6.60E−09	5.73E−08	1.91E−05
**Methylation (K)**	No labels	Avg	0.7600	0.7454	0.7239	0.5804	0.7132	0.4209	0.6225	0.8097
		STD	0.0661	0.0924	0.0587	0.1514	0.1052	0.1118	0.0968	0.1348
	Labels	Avg	0.7815	0.7725	0.7383	0.5998	0.7206	0.4504	0.6464	0.8300
		STD	0.0601	0.0667	0.0649	0.1141	0.1392	0.1466	0.1011	0.0930
		P value	0.4805	0.4867	0.6273	0.7635	0.9008	0.6376	0.6133	0.7152
**Methylation (R)**	No labels	Avg	0.9216	0.9197	0.8599	0.8418	0.8656	0.7211	0.8523	0.8770
		STD	0.0109	0.0162	0.0194	0.0431	0.0359	0.0384	0.0233	0.0387
	Labels	Avg	0.9472	0.9528	0.8861	0.8596	0.9015	0.7739	0.8789	0.9114
		STD	0.0088	0.0098	0.0100	0.0360	0.0297	0.0190	0.0123	0.0298
		P value	3.83E−05	1.06E−04	3.06E−03	3.55E−01	3.36E−02	2.60E−03	9.08E−03	5.00E−02
**Pyroglutamylation (Q)**	No labels	Avg	0.9205	0.9352	0.8312	0.7940	0.8438	0.6704	0.8016	0.8782
		STD	0.0848	0.0771	0.0568	0.1415	0.1609	0.1284	0.0954	0.1073
	Labels	Avg	0.9173	0.9262	0.8363	0.7995	0.8381	0.6695	0.8078	0.8718
		STD	0.0875	0.0795	0.0701	0.1232	0.1609	0.1511	0.1102	0.1233
		P value	0.9388	0.8105	0.8670	0.9302	0.9411	0.9899	0.9001	0.9080
**Palmitoylation (C)**	No labels	Avg	0.8388	0.8346	0.7213	0.5779	0.8133	0.4631	0.6668	0.8633
		STD	0.0580	0.0685	0.0685	0.1410	0.0649	0.1251	0.1073	0.0547
	Labels	Avg	0.9353	0.9436	0.8527	0.8380	0.8681	0.7068	0.8514	0.8688
		STD	0.0260	0.0248	0.0467	0.0575	0.0568	0.0932	0.0466	0.0599
		P value	0.0006	0.0009	0.0002	0.0003	0.0728	0.0002	0.0005	0.8411

**Hydroxylation (P)**	No labels	Avg	0.9813	0.9807	0.9635	0.9429	0.9851	0.9274	0.9633	0.9847
		STD	0.0184	0.0273	0.0229	0.0364	0.0175	0.0457	0.0249	0.0165
	Labels	Avg	0.9893	0.9928	0.9750	0.9606	0.9898	0.9499	0.9748	0.9889
		STD	0.0118	0.0081	0.0141	0.0272	0.0103	0.0282	0.0161	0.0112
		P value	0.2905	0.2299	0.2214	0.2594	0.4938	0.2266	0.2621	0.5454
**Hydroxylation (K)**	No labels	Avg	0.9710	0.9815	0.9765	0.9600	0.9933	0.9423	0.9749	0.9667
		STD	0.0547	0.0418	0.0288	0.0663	0.0200	0.0751	0.0358	0.1000
	Labels	Avg	0.9752	0.9785	0.9647	0.9400	0.9790	0.9165	0.9561	0.9576
		STD	0.0399	0.0366	0.0288	0.0800	0.0452	0.0732	0.0425	0.1007
		P value	0.8517	0.8760	0.3979	0.5711	0.4027	0.4702	0.3237	0.8497

S5.2. Model results in All-Organism Datasets from MusiteDeep

PTM			AUC	AUPRC	Accuracy	Recall	Precision	MCC	F1	Specificity
**Phosphorylation (S,T)**	No labels	Avg	0.8690	0.8527	0.7928	0.8017	0.7882	0.5862	0.7945	0.7838
		STD	0.0129	0.0117	0.0120	0.0301	0.0115	0.0239	0.0143	0.0202
	Labels	Avg	0.9275	0.9217	0.8564	0.8714	0.8463	0.7132	0.8586	0.8414
		STD	0.0038	0.0042	0.0054	0.0072	0.0098	0.0106	0.0049	0.0126
		P value	7.25E−08	2.78E−09	3.27E−09	5.06E−05	1.27E−09	3.62E−09	5.74E−08	2.63E−06
**Phosphorylation (Y)**	No labels	Avg	0.8921	0.8556	0.8342	0.8027	0.7970	0.6370	0.7993	0.8270
		STD	0.0319	0.0302	0.0164	0.0595	0.0282	0.0588	0.0409	0.1123
	Labels	Avg	0.9008	0.8693	0.8356	0.8336	0.7857	0.6497	0.8080	0.8211
		STD	0.0263	0.0320	0.0138	0.0297	0.0480	0.0453	0.0305	0.0706
		P value	0.5396	0.3609	0.8549	0.1863	0.5528	0.6147	0.6141	0.8955
**N-Glycosylation (N)**	No labels	Avg	0.9823	0.9691	0.9753	0.9967	0.9538	0.9478	0.9747	0.9509
		STD	0.0013	0.0109	0.0076	0.0013	0.0151	0.0040	0.0082	0.0027
	Labels	Avg	0.9887	0.9842	0.9767	0.9957	0.9574	0.9494	0.9761	0.9539
		STD	0.0019	0.0056	0.0070	0.0022	0.0141	0.0026	0.0076	0.0061
		P value	4.10E−07	2.50E−03	6.86E−01	2.55E−01	6.03E−01	3.08E−01	7.09E−01	1.99E−01
**O-Glycosylation (S,T)**	No labels	Avg	0.9472	0.9332	0.8691	0.8698	0.8188	0.7326	0.8412	0.8704
		STD	0.0183	0.0284	0.0230	0.0366	0.0746	0.0519	0.0409	0.0453
	Labels	Avg	0.9598	0.9435	0.8921	0.8930	0.8545	0.7811	0.8708	0.8929
		STD	0.0191	0.0248	0.0412	0.0598	0.0646	0.0778	0.0432	0.0628
		P value	0.1722	0.4237	0.1660	0.3370	0.2917	0.1395	0.1523	0.3973
**Ubiquitination (K)**	No labels	Avg	0.8876	0.8014	0.8256	0.7980	0.7138	0.6210	0.7519	0.8370
		STD	0.0302	0.0545	0.0304	0.0478	0.0598	0.0609	0.0416	0.0494
	Labels	Avg	0.9228	0.8623	0.8740	0.8397	0.7967	0.7216	0.8165	0.8910
		STD	0.0215	0.0291	0.0289	0.0424	0.0426	0.0546	0.0299	0.0340
		P value	0.0114	0.0106	0.0028	0.0664	0.0037	0.0017	0.0016	0.0159
**SUMOylation (K)**	No labels	Avg	0.9566	0.9302	0.9067	0.8912	0.8687	0.8033	0.8773	0.9194
		STD	0.0165	0.0321	0.0262	0.0392	0.0736	0.0557	0.0379	0.0387

	Labels	Avg	0.9661	0.9448	0.9135	0.9096	0.8699	0.8185	0.8867	0.9191
		STD	0.0167	0.0239	0.0277	0.0480	0.0725	0.0598	0.0408	0.0373
		P value	0.2418	0.2872	0.5972	0.3849	0.9717	0.5837	0.6182	0.9865
**Acetylation (K)**	No labels	Avg	0.8631	0.7605	0.7937	0.7567	0.7023	0.5637	0.7281	0.8147
		STD	0.0125	0.0204	0.0144	0.0180	0.0243	0.0243	0.0123	0.0270
	Labels	Avg	0.9044	0.8361	0.8357	0.8215	0.7541	0.6559	0.7850	0.8441
		STD	0.0185	0.0239	0.0192	0.0342	0.0389	0.0331	0.0195	0.0380
		P value	4.73E−05	1.18E−06	6.96E−05	2.01E−04	4.05E−03	4.03E−06	2.06E−06	7.65E−02
**Methylation (K)**	No labels	Avg	0.9648	0.9046	0.9213	0.8915	0.8252	0.8039	0.8542	0.9338
		STD	0.0161	0.0475	0.0228	0.0514	0.0755	0.0565	0.0420	0.0287
	Labels	Avg	0.9677	0.9167	0.9224	0.8851	0.8293	0.8038	0.8538	0.9367
		STD	0.0104	0.0367	0.0222	0.0494	0.0753	0.0586	0.0466	0.0267
		P value	0.6586	0.5539	0.9228	0.7921	0.9078	0.9993	0.9862	0.8318
**Methylation (R)**	No labels	Avg	0.9282	0.8695	0.8643	0.8672	0.7930	0.7191	0.8277	0.8625
		STD	0.0171	0.0247	0.0195	0.0389	0.0317	0.0404	0.0250	0.0266
	Labels	Avg	0.9451	0.9062	0.8842	0.8802	0.8245	0.7579	0.8512	0.8865
		STD	0.0134	0.0161	0.0147	0.0237	0.0219	0.0298	0.0180	0.0186
		P value	0.0321	0.0019	0.0254	0.4076	0.0263	0.0334	0.0360	0.0413
**Pyroglutamylation (Q)**	No labels	Avg	0.9610	0.9414	0.9072	0.8850	0.9049	0.8128	0.8927	0.9239
		STD	0.0132	0.0307	0.0306	0.0812	0.0242	0.0630	0.0468	0.0189
	Labels	Avg	0.9797	0.9781	0.9408	0.9346	0.9338	0.8808	0.9337	0.9472
		STD	0.0080	0.0085	0.0126	0.0309	0.0249	0.0259	0.0167	0.0142
		P value	0.0025	0.0059	0.0100	0.1135	0.0223	0.0112	0.0304	0.0093
**Palmitoylation (C)**	No labels	Avg	0.9492	0.9236	0.8832	0.8061	0.8963	0.7570	0.8471	0.9375
		STD	0.0168	0.0216	0.0328	0.0629	0.0282	0.0600	0.0358	0.0197
	Labels	Avg	0.9721	0.9493	0.9339	0.9314	0.9064	0.8629	0.9180	0.9369
		STD	0.0085	0.0218	0.0158	0.0304	0.0342	0.0322	0.0189	0.0210
		P value	0.0029	0.0216	0.0011	0.0001	0.5031	0.0004	0.0001	0.9491
**Hydroxylation (P)**	No labels	Avg	0.9088	0.8562	0.8319	0.7685	0.8086	0.6497	0.7874	0.8750
		STD	0.0125	0.0267	0.0129	0.0350	0.0247	0.0282	0.0198	0.0207

	Labels	Avg	0.9796	0.9676	0.9426	0.9404	0.9196	0.8815	0.9298	0.9442
		STD	0.0048	0.0088	0.0123	0.0217	0.0162	0.0263	0.0166	0.0091
		P value	3.15E−09	1.35E−07	3.44E−13	2.31E−09	7.19E−09	6.14E−13	4.20E−12	7.00E−07
**Hydroxylation (K)**	No labels	Avg	0.9186	0.8368	0.8470	0.7803	0.7842	0.6602	0.7734	0.8915
		STD	0.0330	0.0517	0.0465	0.1016	0.0871	0.0761	0.0473	0.0401
	Labels	Avg	0.9472	0.8763	0.8687	0.8777	0.7683	0.7154	0.8016	0.8732
		STD	0.0211	0.0985	0.0440	0.0606	0.1731	0.1101	0.1283	0.0562
		P value	0.0450	0.3054	0.3222	0.0262	0.8097	0.2336	0.5476	0.4374

S6. Additional ROC Curves

S6.1. AUC for Models Trained on MusiteDeep Datasets

Phosphorylation (S,T)

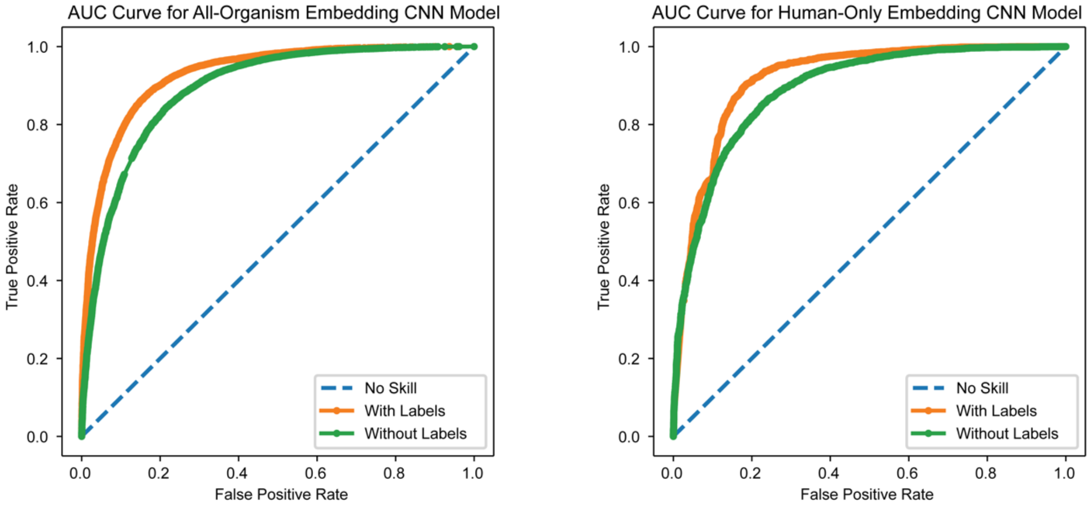

Phosphorylation (Y)

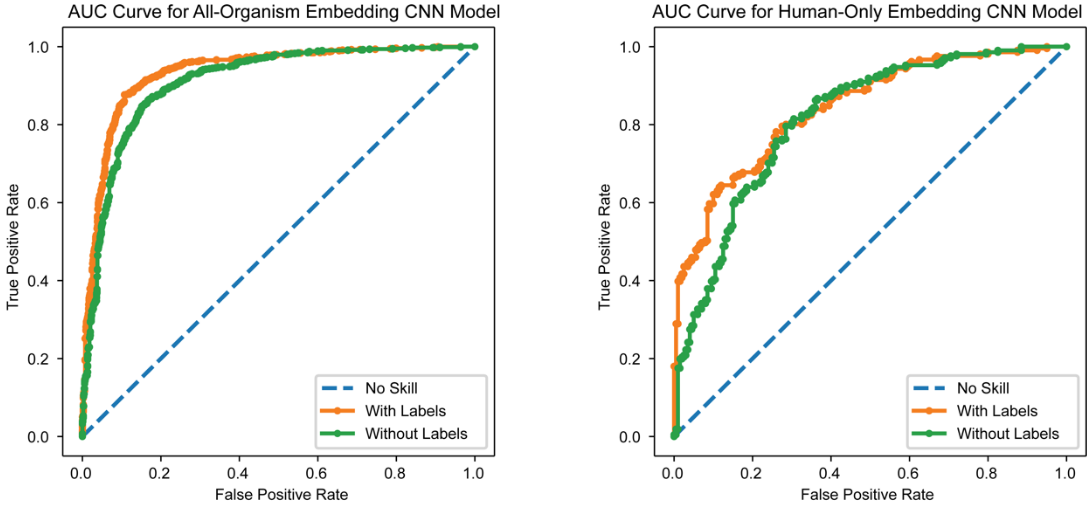

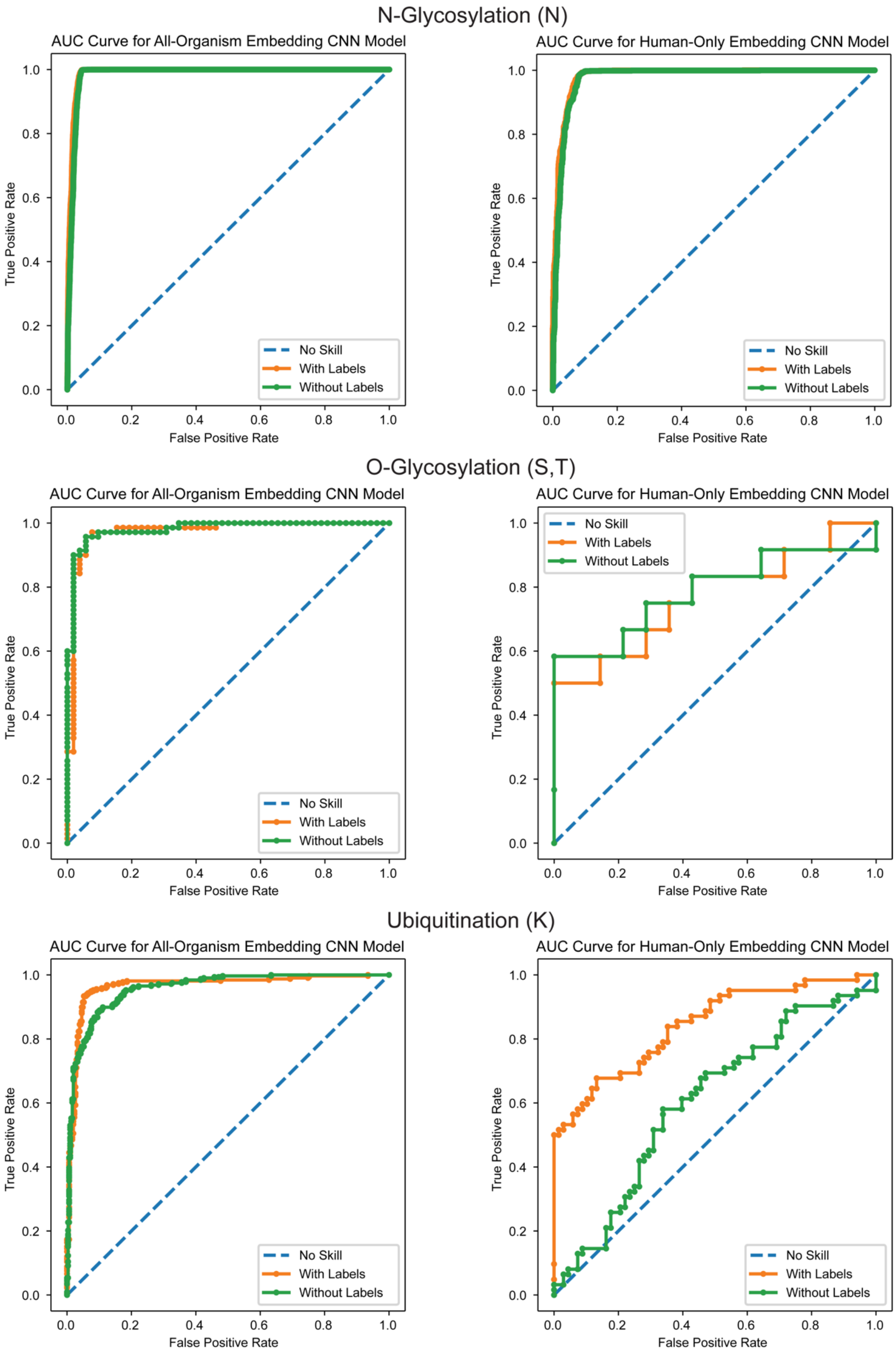
N-Glycosylation (N)
AUC Curve for All-Organism Embedding CNN Model
AUC Curve for Human-Only Embedding CNN Model
O-Glycosylation (S,T)
AUC Curve for All-Organism Embedding CNN Model
AUC Curve for Human-Only Embedding CNN Model
Ubiquitination (K)
AUC Curve for All-Organism Embedding CNN Model
AUC Curve for Human-Only Embedding CNN Model
True Positive Rate
False Positive Rate
0.0
0.2
0.4
0.6
0.8
1.0
No Skill
With Labels
Without Labels

SUMOylation (K)

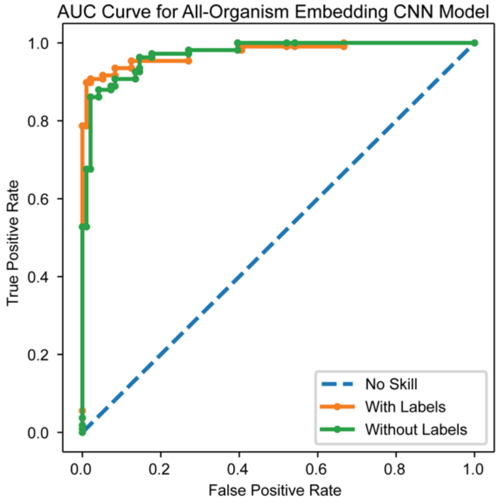

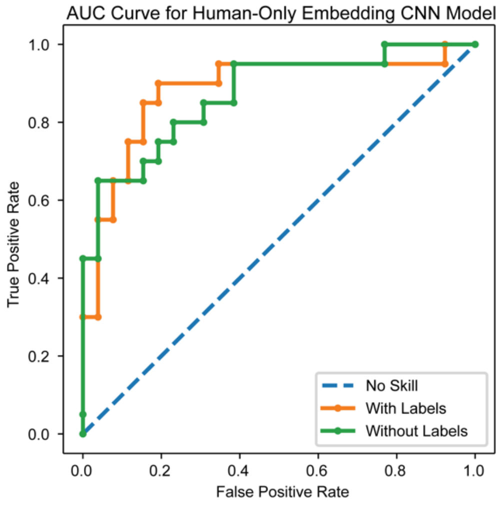

Acetylation (K)

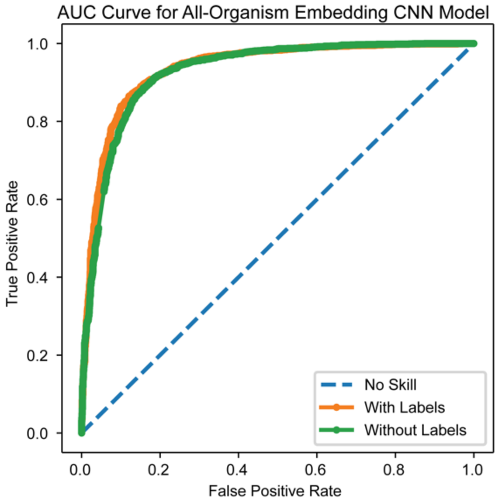

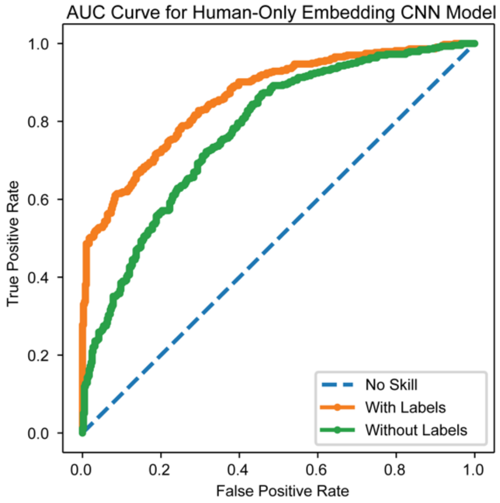

Methylation (K)

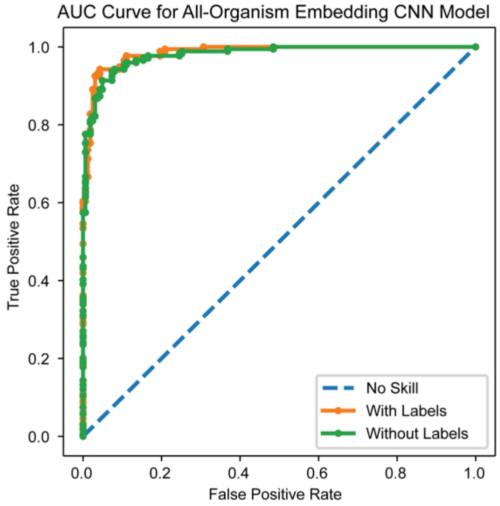

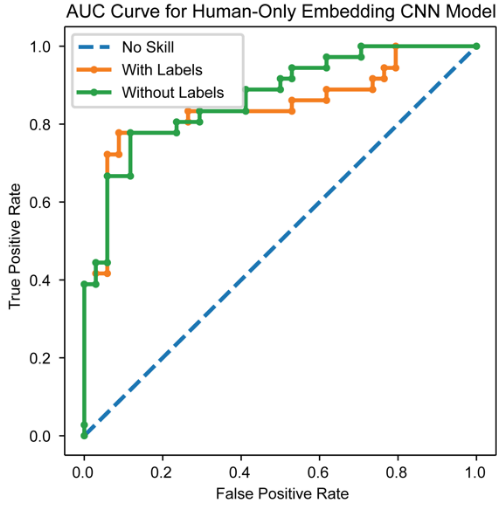

Methylation (R)

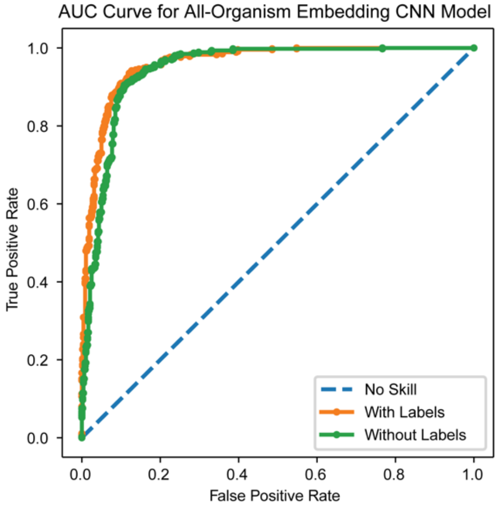

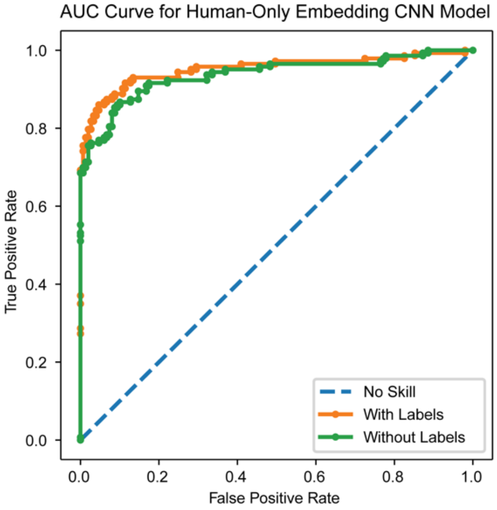

Pyroglutamylation (Q)

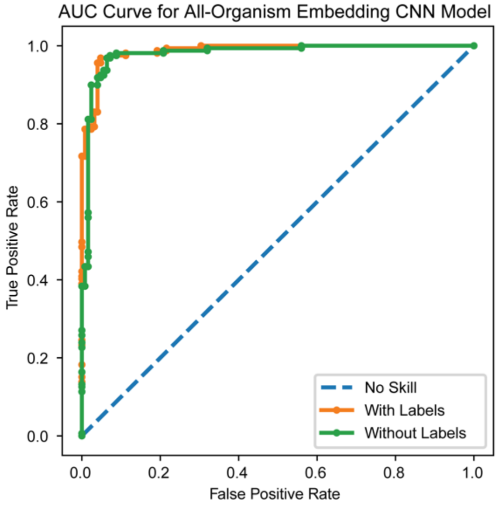

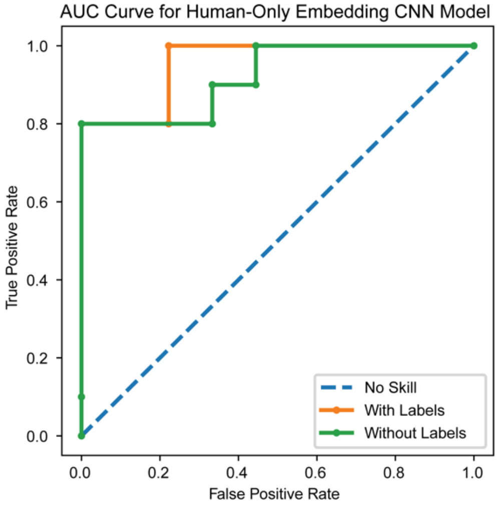

Palmitoylation (C)

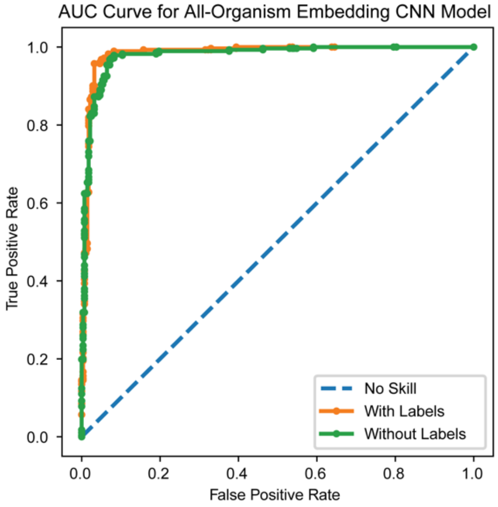

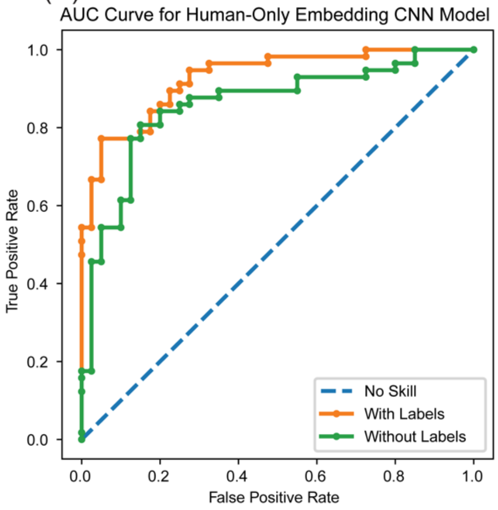

Hydroxylation (P)

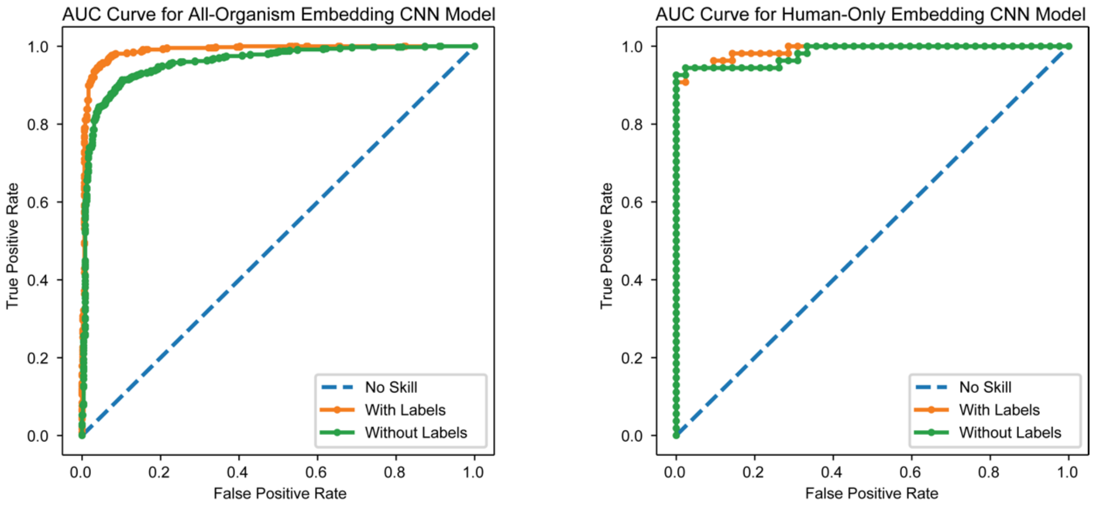

Hydroxylation (K)

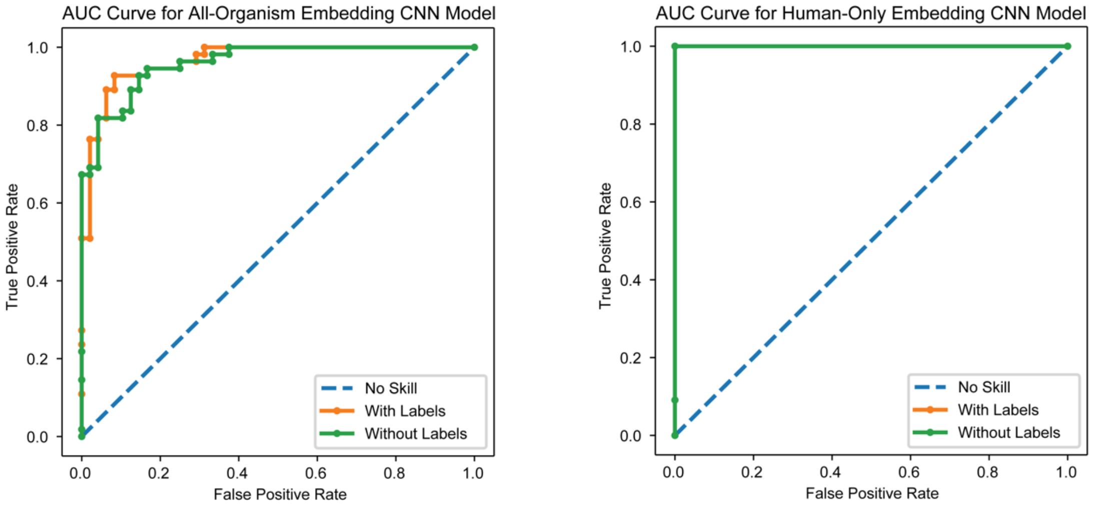

S6.2. AUPRC for Models Trained on MusiteDeep Datasets

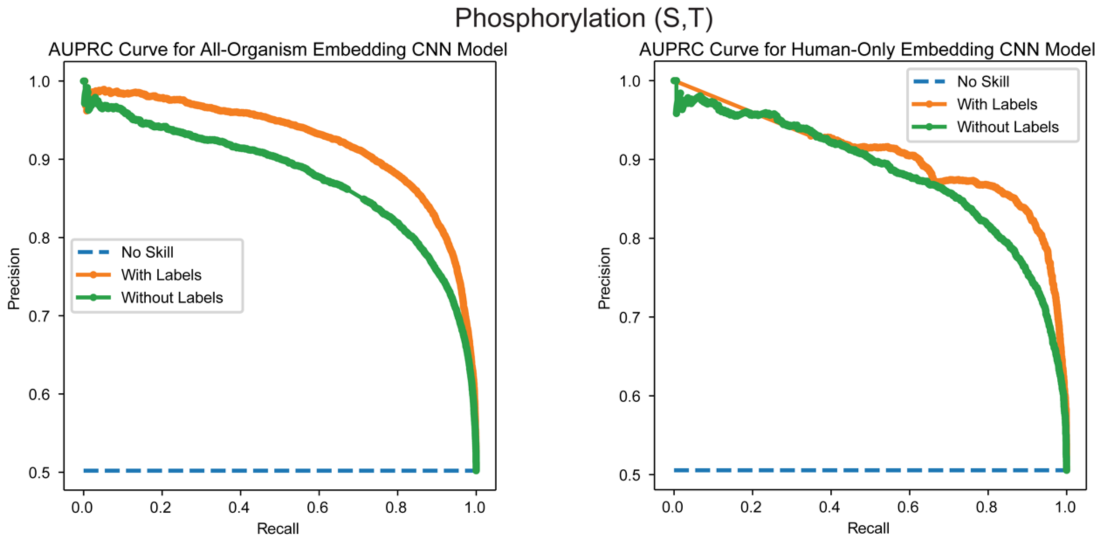

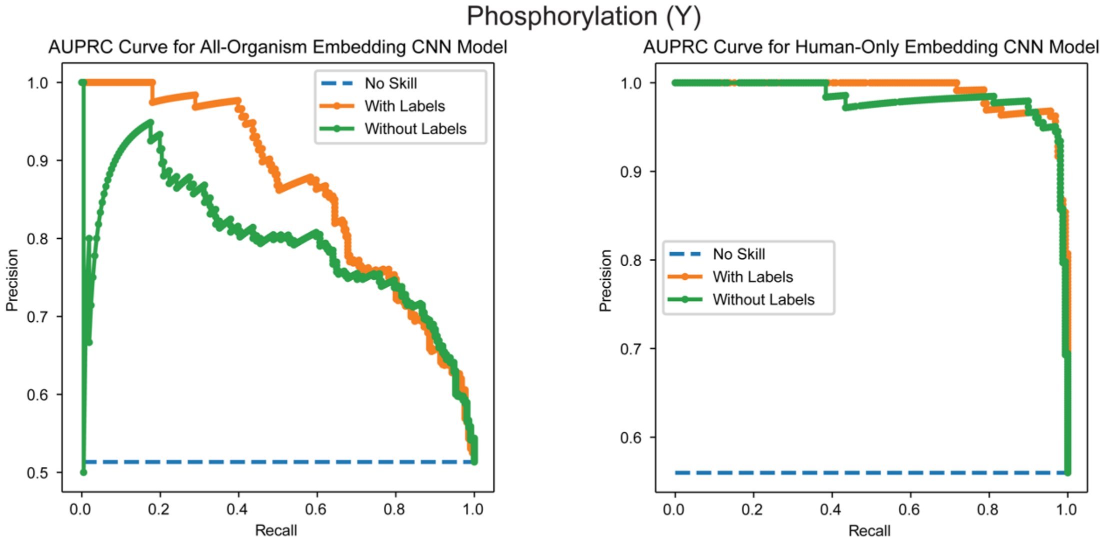

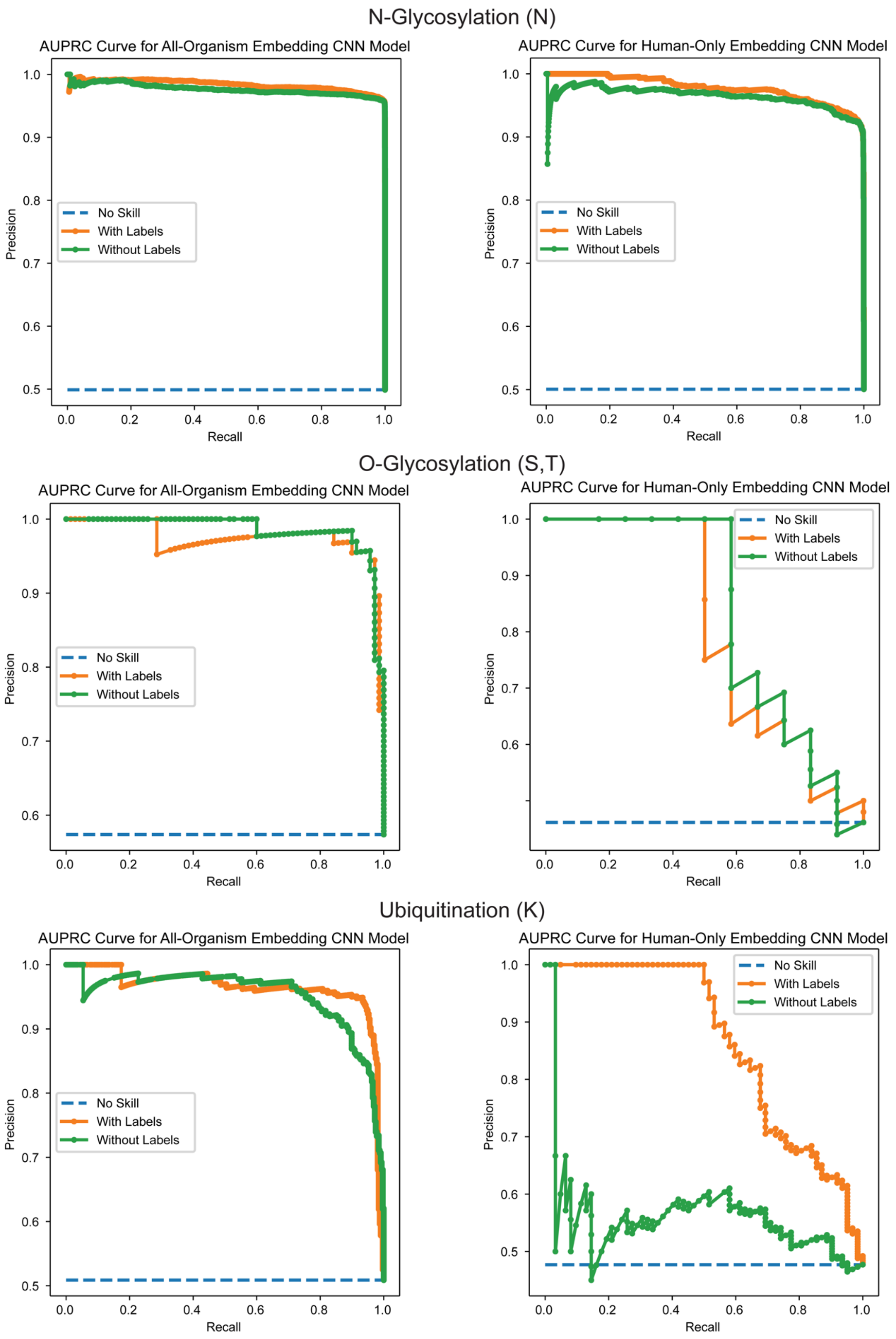

N-Glycosylation (N)
AUPRC Curve for All-Organism Embedding CNN Model
AUPRC Curve for Human-Only Embedding CNN Model
O-Glycosylation (S,T)
AUPRC Curve for All-Organism Embedding CNN Model
AUPRC Curve for Human-Only Embedding CNN Model
Ubiquitination (K)
AUPRC Curve for All-Organism Embedding CNN Model
AUPRC Curve for Human-Only Embedding CNN Model
Precision
Recall
No Skill
With Labels
Without Labels

SUMOylation (K)

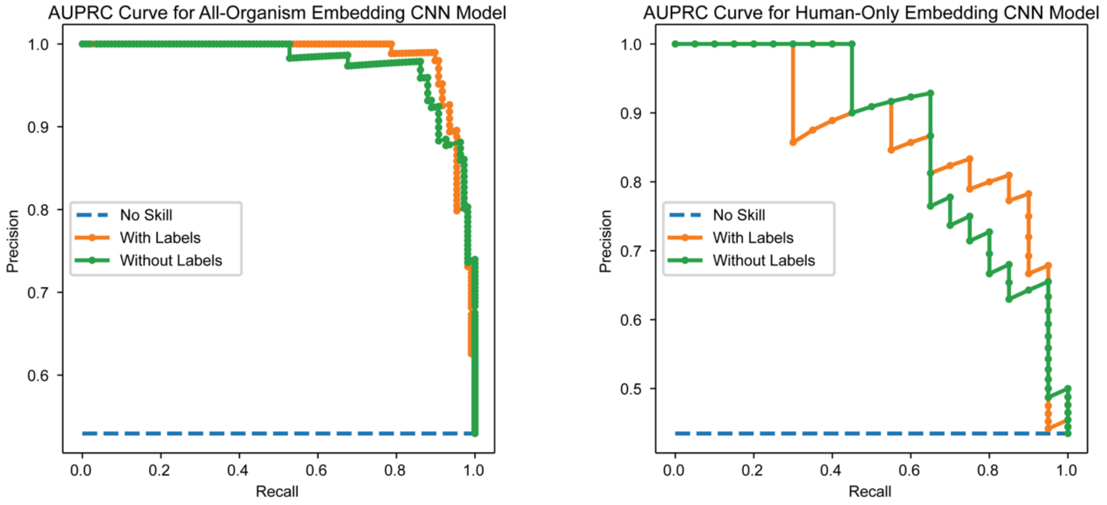

Acetylation (K)

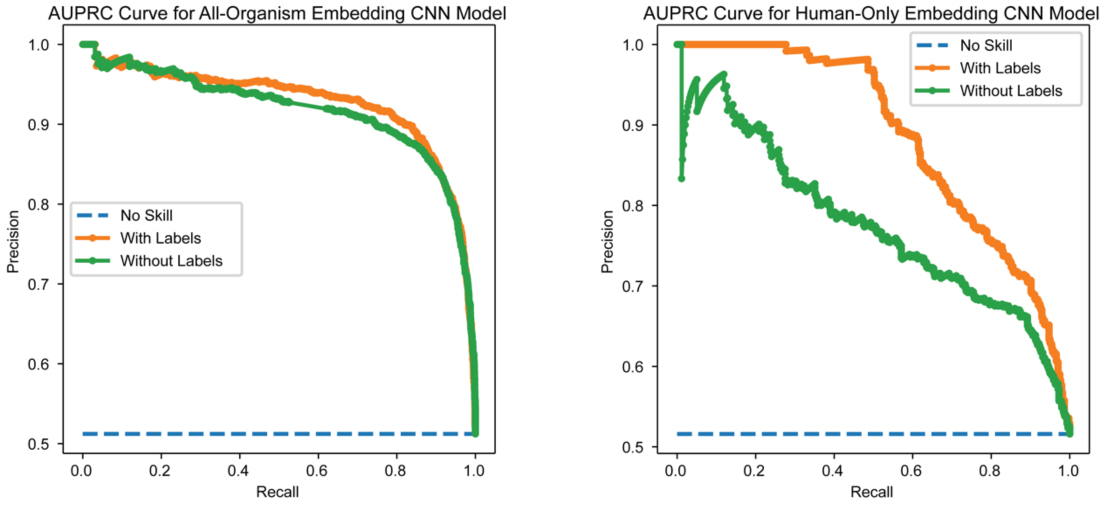

Methylation (K)

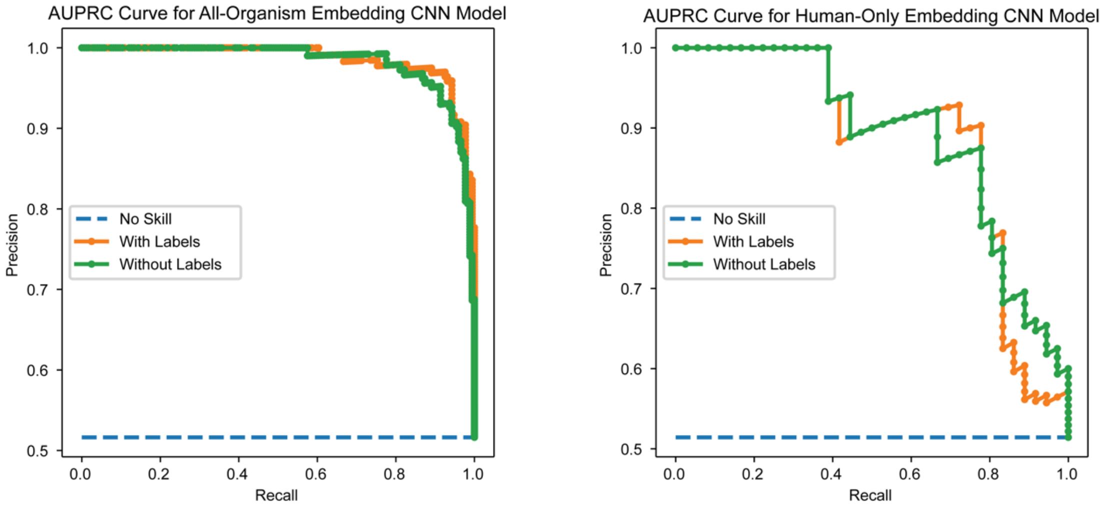

Methylation (R)

AUPRC Curve for All-Organism Embedding CNN Model

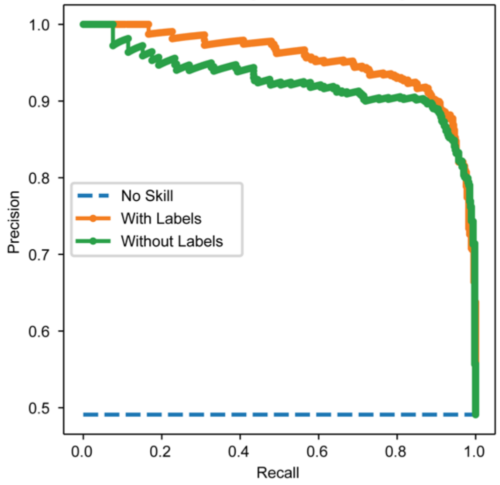

AUPRC Curve for Human-Only Embedding CNN Model

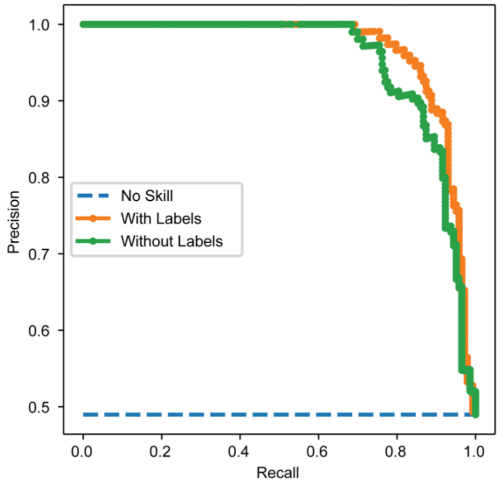

Pyroglutamylation (Q)

AUPRC Curve for All-Organism Embedding CNN Model

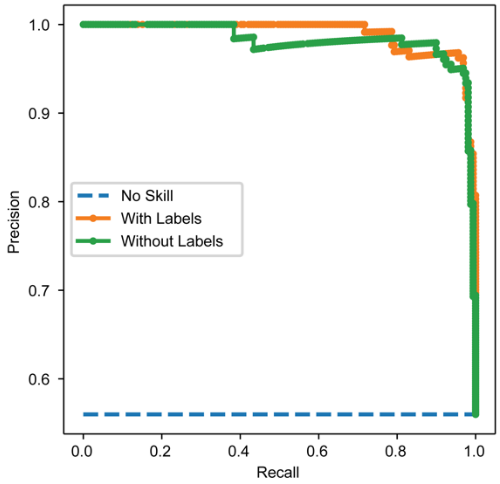

AUPRC Curve for Human-Only Embedding CNN Model

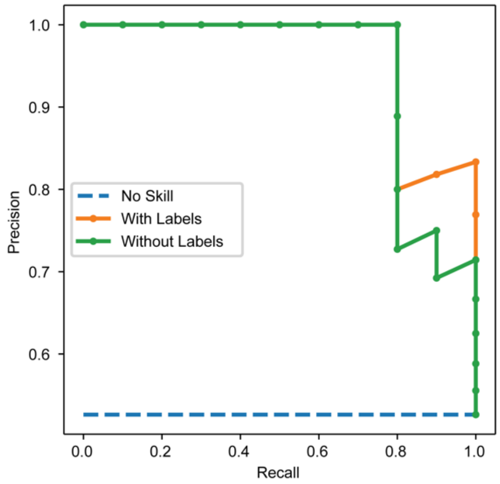

Palmitoylation (C)

AUPRC Curve for All-Organism Embedding CNN Model

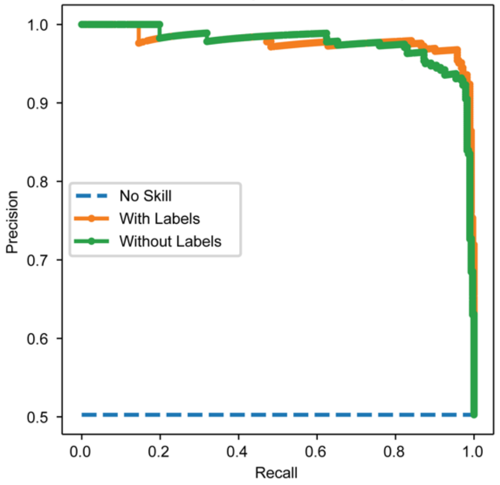

AUPRC Curve for Human-Only Embedding CNN Model

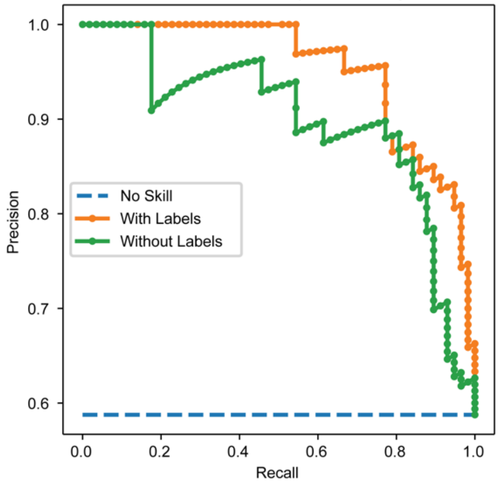

Hydroxylation (P)

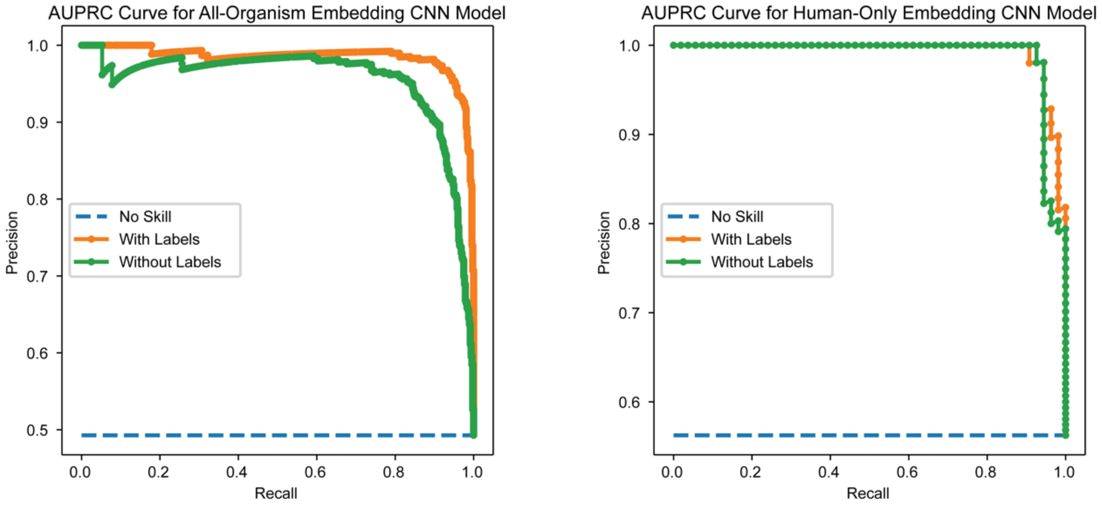

Hydroxylation (K)

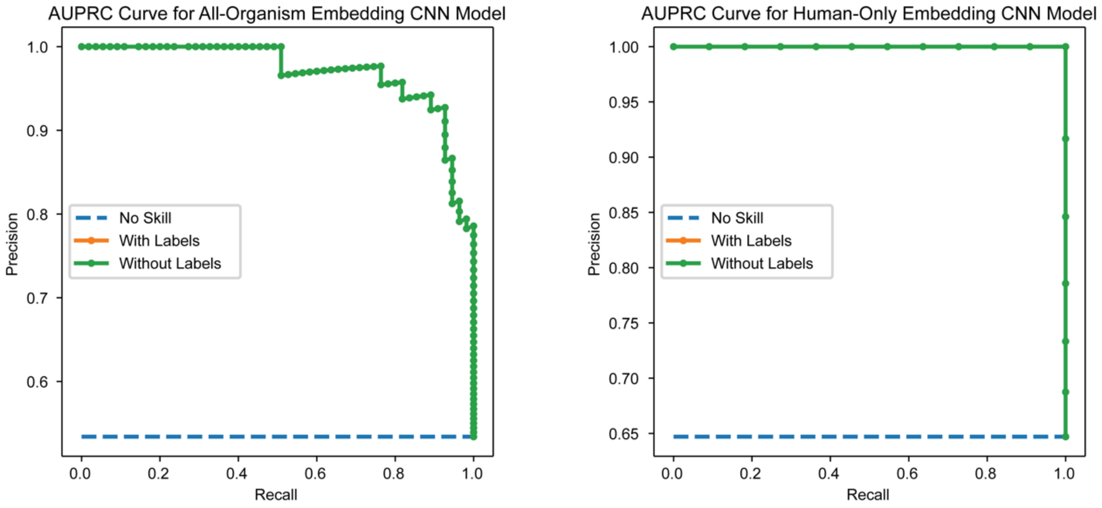

S7. Additional O-Glycosylation Models

S7.1 Model Results

PTM			AUC	AUPRC	Accuracy	Recall	Precision	MCC	F1	Specificity
**OGP Full Dataset, All-organism**	No labels	Avg	0.8647	0.8670	0.7772	0.8023	0.7624	0.5558	0.7814	0.7525
		STD	0.0175	0.0190	0.0167	0.0274	0.0194	0.0334	0.0152	0.0276
	Labels	Avg	0.9574	0.9653	0.8983	0.8637	0.9268	0.7985	0.8939	0.9324
		STD	0.0065	0.0066	0.0058	0.0162	0.0132	0.0116	0.0073	0.0139
		P value	7.82E−09	1.29E−08	3.19E−10	3.99E−05	4.87E−13	3.13E−10	3.89E−11	1.46E−10
**OGP Full Dataset, Human-only**	No labels	Avg	0.8544	0.8593	0.7635	0.7751	0.7534	0.5277	0.7635	0.7516
		STD	0.0163	0.0234	0.0183	0.0423	0.0238	0.0378	0.0261	0.0267
	Labels	Avg	0.9531	0.9631	0.8970	0.8534	0.9328	0.7968	0.8912	0.9399
		STD	0.0052	0.0038	0.0079	0.0176	0.0119	0.0147	0.0085	0.0103
		P value	3.24E−09	2.14E−07	1.01E−10	2.53E−04	2.44E−11	2.23E−10	2.74E−08	2.77E−10
**OGP GalNAc only, All-organism**	No labels	Avg	0.8939	0.8990	0.8060	0.8441	0.7851	0.6141	0.8131	0.7678
		STD	0.0154	0.0161	0.0155	0.0211	0.0271	0.0295	0.0167	0.0313
	Labels	Avg	0.9625	0.9682	0.8988	0.8721	0.9218	0.7987	0.8961	0.9251
		STD	0.0044	0.0037	0.0088	0.0145	0.0134	0.0177	0.0094	0.0158
		P value	9.51E−08	1.95E−07	2.17E−10	4.79E−03	4.07E−09	9.39E−11	2.99E−09	3.97E−09
**OGP GalNAc Only Human-only**	No labels	Avg	0.8848	0.8781	0.7898	0.7918	0.7889	0.5828	0.7862	0.7865
		STD	0.0403	0.0618	0.0468	0.1128	0.0344	0.0907	0.0723	0.0464
	Labels	Avg	0.9721	0.9781	0.9233	0.8849	0.9595	0.8492	0.9206	0.9620
		STD	0.0035	0.0018	0.0078	0.0109	0.0118	0.0155	0.0071	0.0121
		P value	1.07E−04	9.05E−04	1.03E−05	3.55E−02	2.00E−08	7.84E−06	3.33E−04	5.48E−07
**OGP HexNAc Only All-organism**	No labels	Avg	0.7961	0.7724	0.7252	0.7654	0.7039	0.4528	0.7327	0.6855
		STD	0.0193	0.0155	0.0225	0.0406	0.0222	0.0443	0.0246	0.0359
	Labels	Avg	0.9139	0.9166	0.8319	0.8219	0.8368	0.6652	0.8280	0.8419
		STD	0.0050	0.0071	0.0096	0.0381	0.0260	0.0175	0.0110	0.0335
		P value	5.38E−09	3.38E−12	1.59E−08	6.99E−03	1.08E−09	1.82E−08	1.31E−07	1.90E−08

**OGP HexNAc Only, Human-only**	No labels	Avg	0.8463	0.8218	0.7702	0.8104	0.7489	0.5440	0.7774	0.7312
		STD	0.0165	0.0253	0.0147	0.0446	0.0214	0.0329	0.0180	0.0295
	Labels	Avg	0.9366	0.9436	0.8619	0.8458	0.8727	0.7238	0.8589	0.8771
		STD	0.0079	0.0073	0.0108	0.0111	0.0178	0.0217	0.0098	0.0226
		P value	1.67E−09	4.48E−08	4.39E−11	4.28E−02	1.42E−10	4.34E−10	1.06E−08	1.47E−09
**O-GlcNAc Site Atlas, All-organism**	No labels	Avg	0.7744	0.7647	0.6994	0.6704	0.7128	0.4015	0.6888	0.7282
		STD	0.0135	0.0194	0.0112	0.0570	0.0260	0.0229	0.0235	0.0523
	Labels	Avg	0.8794	0.8898	0.7965	0.7621	0.8179	0.5946	0.7887	0.8306
		STD	0.0055	0.0062	0.0073	0.0201	0.0150	0.0147	0.0095	0.0191
		P value	6.55E−11	1.64E−09	4.35E−13	7.81E−04	3.97E−08	7.94E−13	6.55E−08	1.61E−04
**O-GlcNAc Site Atlas, Human-only**	No labels	Avg	0.7670	0.7631	0.7008	0.6154	0.7376	0.4072	0.6693	0.7847
		STD	0.0311	0.0337	0.0280	0.0620	0.0276	0.0530	0.0421	0.0316
	Labels	Avg	0.9122	0.9235	0.8281	0.7668	0.8717	0.6612	0.8154	0.8886
		STD	0.0035	0.0043	0.0061	0.0210	0.0215	0.0128	0.0080	0.0222
		P value	1.70E−07	1.31E−07	1.26E−07	2.43E−05	1.93E−09	6.46E−08	1.72E−06	4.57E−07

S7.2 AUC ROC Curves

OGP O-Glycosylation

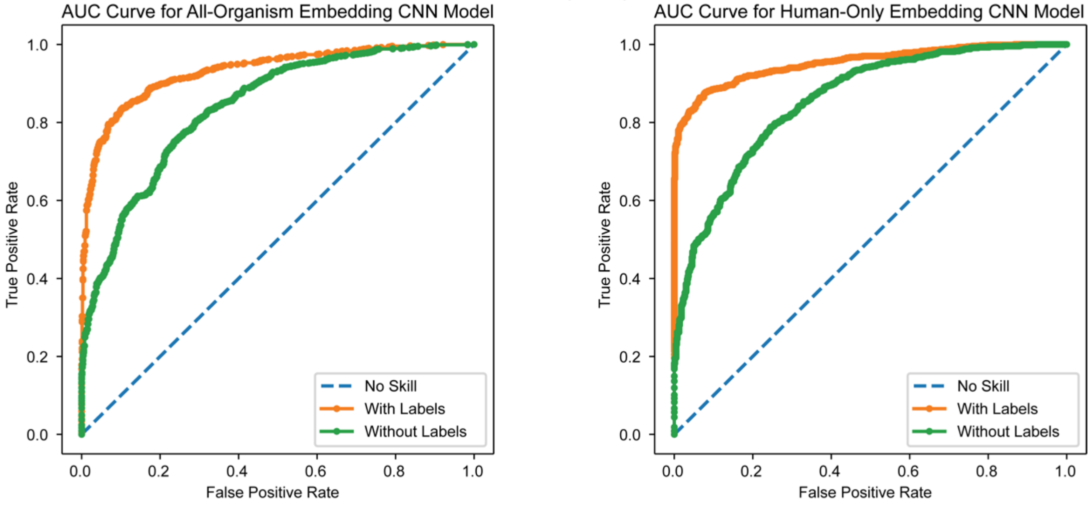

OGP GalNAc

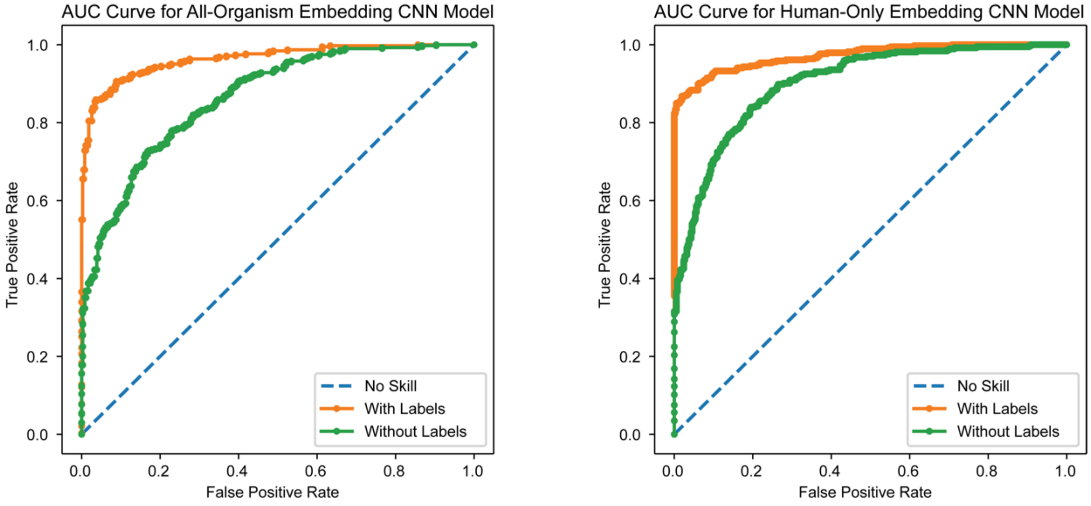

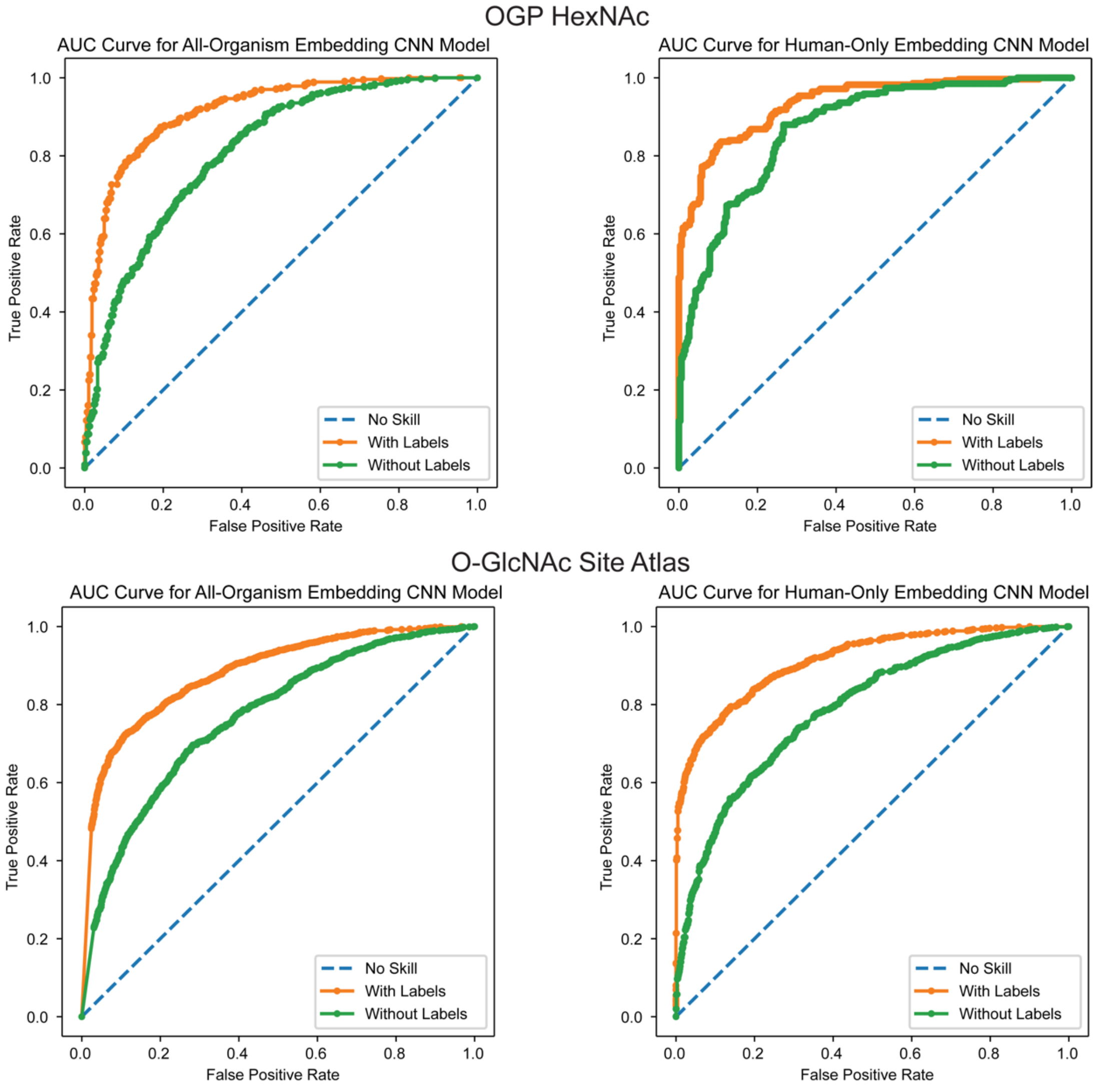
OGP HexNAc
AUC Curve for All-Organism Embedding CNN Model
AUC Curve for Human-Only Embedding CNN Model
True Positive Rate
False Positive Rate
No Skill
With Labels
Without Labels
0.0
0.2
0.4
0.6
0.8
1.0
O-GlcNAc Site Atlas
AUC Curve for All-Organism Embedding CNN Model
AUC Curve for Human-Only Embedding CNN Model
True Positive Rate
False Positive Rate
No Skill
With Labels
Without Labels

S7.3. AUPRC ROC curves

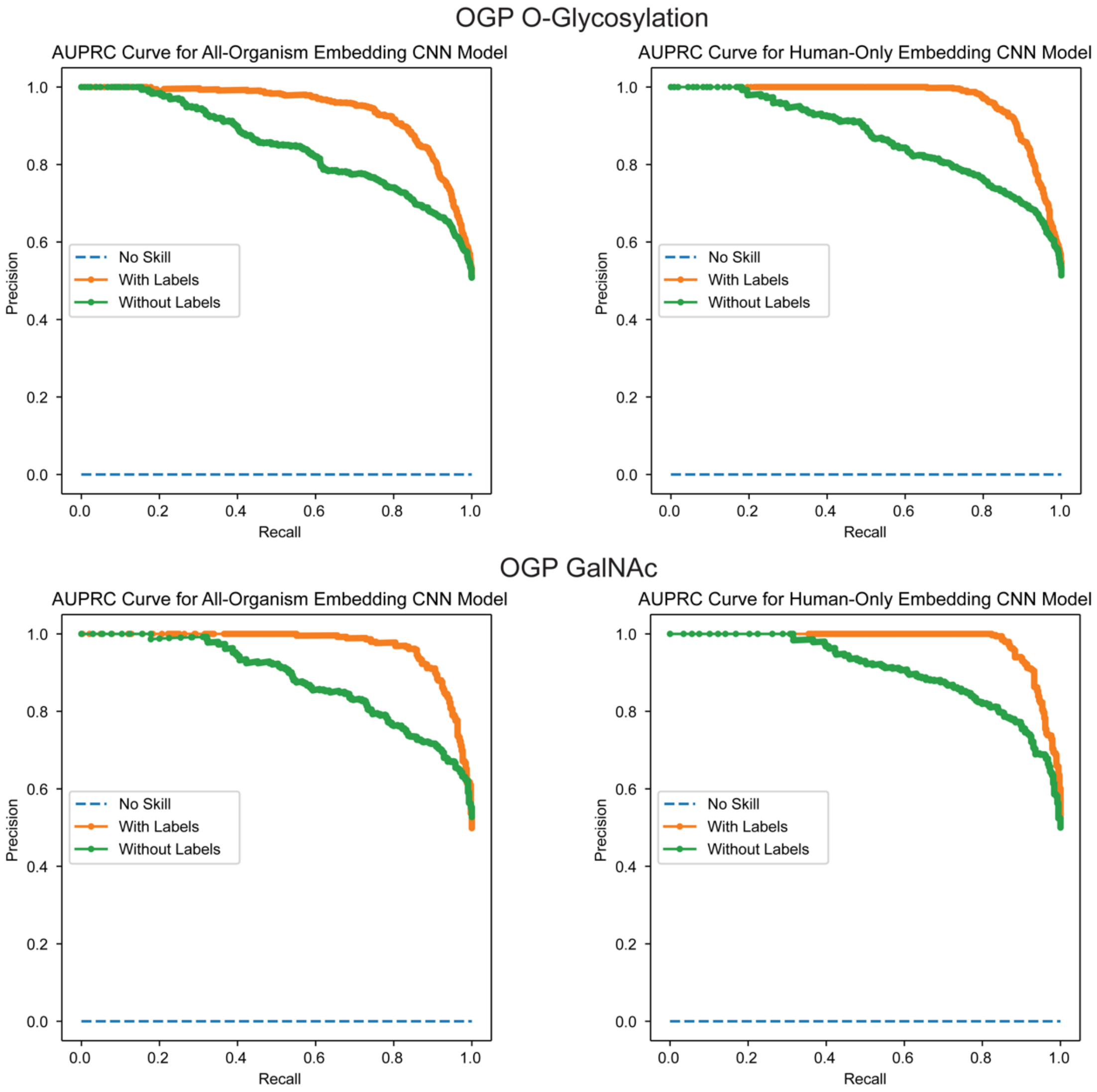

OGP HexNAc

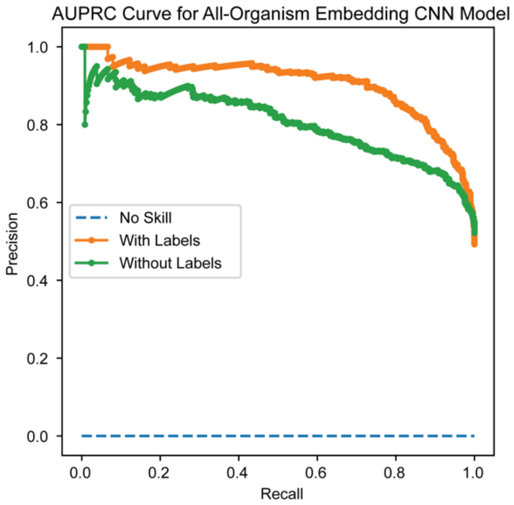

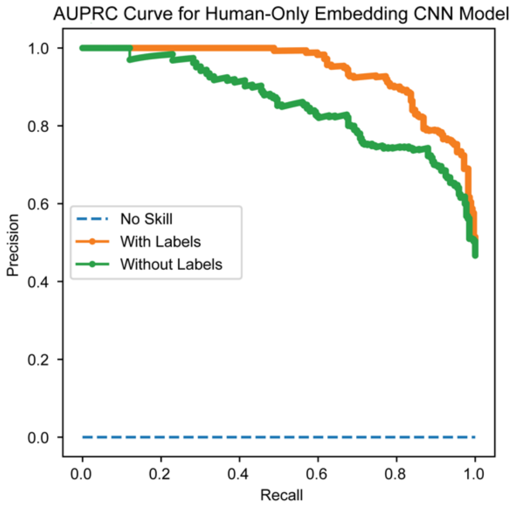

O-GlcNAc Site Atlas

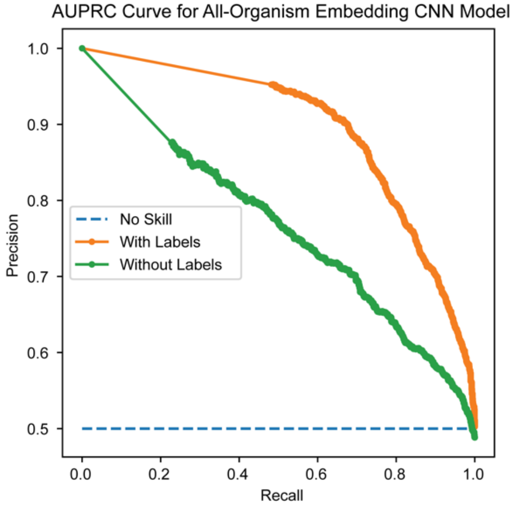

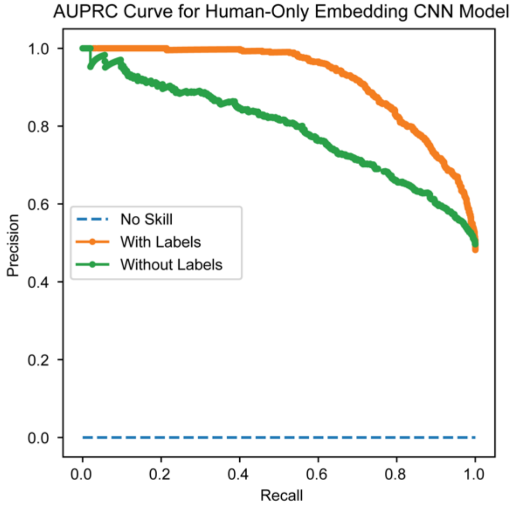

S8. N-Glycosylation Sequon-Specific Models

S8.1. Model Results

PTM			AUC	AUPRC	Accuracy	Recall	Precision	MCC	F1	Specificity
**N-Glycosite Atlas, All-organism**	No labels	Avg	0.6557	0.4819	0.6645	0.1751	0.5350	0.1423	0.2577	0.6645
		STD	0.0114	0.0142	0.0088	0.0634	0.0190	0.0347	0.0752	0.0088
	Labels	Avg	0.6869	0.5209	0.6792	0.2720	0.5664	0.2086	0.3658	0.6792
		STD	0.0260	0.0313	0.0136	0.0340	0.0411	0.0320	0.0315	0.0136
		P value	0.0013	0.0007	0.0215	0.0000	0.0548	0.0001	0.0001	0.0215
**NGlyDE Dataset, Human-only**	No labels	Avg	0.6698	0.7827	0.6913	0.9464	0.7028	0.1838	0.8044	0.6913
		STD	0.0206	0.0307	0.0261	0.0583	0.0393	0.0884	0.0182	0.0261
	Labels	Avg	0.7545	0.8369	0.7270	0.9374	0.7260	0.3420	0.8175	0.7270
		STD	0.0559	0.0477	0.0254	0.0403	0.0279	0.0848	0.0214	0.0254
		P value	0.0001	0.0091	0.0077	0.7117	0.1429	0.0000	0.2180	0.0077

S8.2 AUC ROC Curves

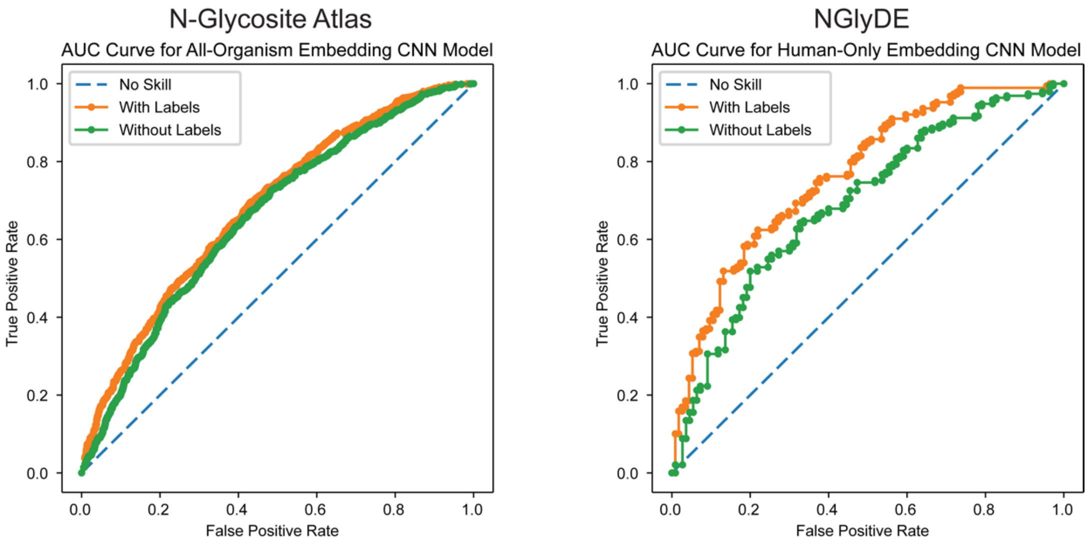

S8.3 AUPRC ROC Curves

N-Glycosite Atlas

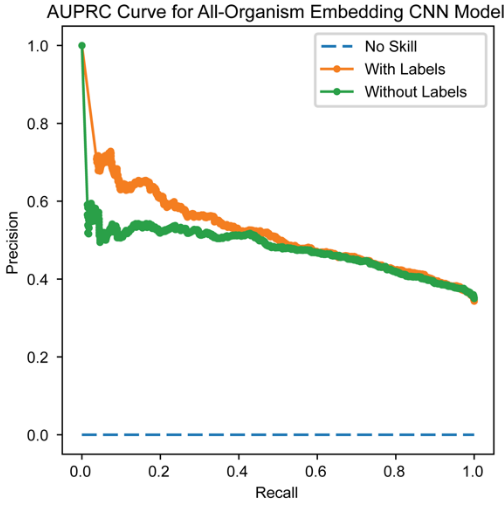

NGlyDE

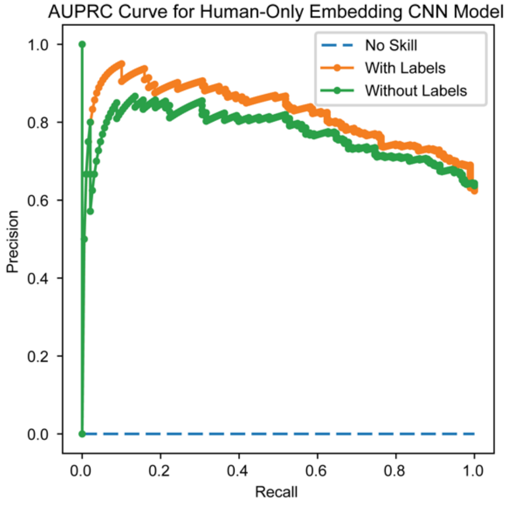

S9. Additional Kinase-Specific Model Results

S9.1. List of Kinases Used

AurA	AurC	CDC2/CycB1	CDK5/p25	CHK1	CK1δ
CaMK2δ	CaMK2γ	DCLK2	DYRK2	DYRK3	Erk1
Erk2	Erk5	GCK	HGK	IKKα	IKKβ
IKKε	JNK1	KHS1	MAP3K1	MAPKAPK3	MAPKAPK5
MARK2	MARK4	MINK	MLK1	MLK2	MLK3
MSK1	MST1	MST2	MST3	MST4	NEK1
NEK2	NEK4	NEK6	NEK7	NEK9	NIM1
NLK	p38α	p38β	p38δ	p38γ	p70S6K
PAK1	PAK2	PAK3	PHKG2	PIM1	PIM3
PKA Cα	PLK1	PLK3	QIK	RSK3	SGK3
SIK	TAK1	TAO2	TLK1	TNIK	TSSK1
TTBK1	YSK1	ZAK			

S9.2 Kinase-Specific Model Results

Kinase			AUC	AUPRC	Accuracy	Recall	Precision	MCC	F1	Specificity
**AurA**	No labels	Average	0.8761	0.8567	0.8188	0.8686	0.7873	0.6428	0.8242	0.8188
		STD	0.0372	0.0600	0.0457	0.0533	0.0670	0.0880	0.0494	0.0457
	Labels	Average	0.9328	0.9174	0.8594	0.8641	0.8563	0.7187	0.8594	0.8594
		STD	0.0201	0.0440	0.0239	0.0387	0.0378	0.0491	0.0275	0.0239
		P value	0.0013	0.0258	0.0336	0.8415	0.0173	0.0401	0.0826	0.0336
**AurC**	No labels	Average	0.9019	0.8788	0.8351	0.8392	0.8430	0.6695	0.8395	0.8351
		STD	0.0401	0.0504	0.0342	0.0469	0.0599	0.0733	0.0392	0.0342
	Labels	Average	0.9363	0.9299	0.8807	0.8923	0.8793	0.7603	0.8848	0.8807
		STD	0.0182	0.0308	0.0143	0.0425	0.0276	0.0290	0.0212	0.0143
		P value	0.0363	0.0204	0.0030	0.0217	0.1226	0.0049	0.0087	0.0030
**CDC2/CycB1**	No labels	Average	0.9256	0.9234	0.8553	0.8461	0.8631	0.7115	0.8529	0.8553
		STD	0.0198	0.0296	0.0261	0.0466	0.0422	0.0510	0.0246	0.0261
	Labels	Average	0.9605	0.9588	0.8947	0.9020	0.8853	0.7901	0.8917	0.8947
		STD	0.0116	0.0155	0.0187	0.0610	0.0327	0.0375	0.0293	0.0187
		P value	0.0004	0.0069	0.0020	0.0435	0.2283	0.0017	0.0072	0.0020
**CDK5/p25**	No labels	Average	0.9000	0.9016	0.8250	0.8396	0.8133	0.6498	0.8254	0.8250
		STD	0.0304	0.0337	0.0385	0.0472	0.0542	0.0752	0.0436	0.0385
	Labels	Average	0.9326	0.9310	0.8630	0.8619	0.8797	0.7263	0.8699	0.8630
		STD	0.0164	0.0183	0.0253	0.0472	0.0157	0.0477	0.0250	0.0253
		P value	0.0135	0.0379	0.0254	0.3317	0.0050	0.0209	0.0185	0.0254
**CHK1**	No labels	Average	0.8887	0.8842	0.8000	0.8107	0.7951	0.6079	0.7979	0.8000
		STD	0.0189	0.0325	0.0327	0.0946	0.0481	0.0627	0.0422	0.0327
	Labels	Average	0.9376	0.9348	0.8663	0.8736	0.8686	0.7343	0.8693	0.8663
		STD	0.0224	0.0305	0.0356	0.0622	0.0448	0.0700	0.0381	0.0356
		P value	0.0001	0.0032	0.0007	0.1158	0.0036	0.0008	0.0014	0.0007
**CK1δ**	No labels	Average	0.9106	0.8950	0.8383	0.8602	0.8222	0.6781	0.8388	0.8383
		STD	0.0179	0.0296	0.0215	0.0557	0.0407	0.0411	0.0277	0.0215

	Labels	Average	0.9552	0.9546	0.8750	0.8933	0.8608	0.7527	0.8752	0.8750
		STD	0.0091	0.0127	0.0255	0.0525	0.0464	0.0521	0.0320	0.0255
		P value	0.0000	0.0001	0.0041	0.2121	0.0770	0.0036	0.0192	0.0041
**CaMK2δ**	No labels	Average	0.8935	0.8865	0.8144	0.8452	0.8047	0.6299	0.8230	0.8144
		STD	0.0301	0.0339	0.0445	0.0701	0.0391	0.0905	0.0452	0.0445
	Labels	Average	0.9385	0.9296	0.8536	0.8800	0.8372	0.7105	0.8561	0.8536
		STD	0.0188	0.0341	0.0302	0.0553	0.0536	0.0589	0.0369	0.0302
		P value	0.0017	0.0151	0.0441	0.2579	0.1605	0.0403	0.1065	0.0441
**CaMK2γ**	No labels	Average	0.9034	0.8875	0.8235	0.8432	0.8076	0.6468	0.8234	0.8235
		STD	0.0250	0.0472	0.0264	0.0527	0.0265	0.0512	0.0219	0.0264
	Labels	Average	0.9466	0.9392	0.8783	0.8734	0.8851	0.7545	0.8787	0.8783
		STD	0.0228	0.0311	0.0346	0.0346	0.0515	0.0683	0.0382	0.0346
		P value	0.0013	0.0147	0.0015	0.1702	0.0014	0.0015	0.0020	0.0015
**DCLK2**	No labels	Average	0.8869	0.8717	0.7969	0.8308	0.7670	0.5988	0.7951	0.7969
		STD	0.0338	0.0594	0.0391	0.0560	0.0629	0.0787	0.0410	0.0391
	Labels	Average	0.9365	0.9367	0.8670	0.8709	0.8735	0.7380	0.8693	0.8670
		STD	0.0241	0.0302	0.0403	0.0839	0.0380	0.0779	0.0439	0.0403
		P value	0.0025	0.0115	0.0015	0.2508	0.0006	0.0014	0.0016	0.0015
**DYRK2**	No labels	Average	0.8718	0.8614	0.7968	0.8224	0.7794	0.5926	0.7995	0.7968
		STD	0.0195	0.0318	0.0212	0.0358	0.0327	0.0442	0.0221	0.0212
	Labels	Average	0.9284	0.9211	0.8552	0.8693	0.8479	0.7131	0.8569	0.8552
		STD	0.0220	0.0320	0.0305	0.0450	0.0528	0.0580	0.0334	0.0305
		P value	0.0000	0.0009	0.0002	0.0255	0.0048	0.0001	0.0006	0.0002
**DYRK3**	No labels	Average	0.8831	0.8626	0.7990	0.7969	0.7950	0.6015	0.7920	0.7990
		STD	0.0294	0.0488	0.0345	0.0660	0.0620	0.0642	0.0375	0.0345
	Labels	Average	0.9305	0.9087	0.8631	0.8918	0.8403	0.7309	0.8628	0.8631
		STD	0.0286	0.0530	0.0273	0.0416	0.0614	0.0541	0.0243	0.0273
		P value	0.0027	0.0712	0.0004	0.0023	0.1367	0.0002	0.0002	0.0004
**Erk1**	No labels	Average	0.8887	0.8773	0.8158	0.8212	0.8117	0.6336	0.8134	0.8158
		STD	0.0350	0.0409	0.0372	0.0635	0.0601	0.0737	0.0397	0.0372

	Labels	Average	0.9361	0.9236	0.8579	0.8860	0.8348	0.7229	0.8566	0.8579
		STD	0.0254	0.0344	0.0382	0.0543	0.0718	0.0741	0.0396	0.0382
		P value	0.0045	0.0185	0.0293	0.0323	0.4684	0.0196	0.0328	0.0293
**Erk2**	No labels	Average	0.9020	0.9061	0.8341	0.8456	0.8338	0.6728	0.8367	0.8341
		STD	0.0174	0.0158	0.0170	0.0617	0.0420	0.0321	0.0212	0.0170
	Labels	Average	0.9539	0.9475	0.8948	0.8946	0.8905	0.7888	0.8918	0.8948
		STD	0.0282	0.0304	0.0361	0.0290	0.0525	0.0726	0.0334	0.0361
		P value	0.0003	0.0029	0.0005	0.0502	0.0214	0.0008	0.0008	0.0005
**Erk5**	No labels	Average	0.8505	0.8470	0.7802	0.7776	0.7960	0.5639	0.7848	0.7802
		STD	0.0465	0.0433	0.0420	0.0734	0.0282	0.0805	0.0420	0.0420
	Labels	Average	0.9298	0.9233	0.8490	0.8443	0.8639	0.6997	0.8508	0.8490
		STD	0.0238	0.0361	0.0376	0.0794	0.0543	0.0738	0.0462	0.0376
		P value	0.0005	0.0008	0.0018	0.0810	0.0052	0.0015	0.0053	0.0018
**GCK**	No labels	Average	0.8565	0.8520	0.7564	0.7788	0.7489	0.5160	0.7610	0.7564
		STD	0.0318	0.0396	0.0266	0.0526	0.0520	0.0523	0.0288	0.0266
	Labels	Average	0.9169	0.9107	0.8336	0.8151	0.8505	0.6703	0.8306	0.8336
		STD	0.0130	0.0260	0.0173	0.0494	0.0464	0.0363	0.0271	0.0173
		P value	0.0002	0.0020	0.0000	0.1493	0.0004	0.0000	0.0001	0.0000
**HGK**	No labels	Average	0.8761	0.8652	0.7877	0.7797	0.7919	0.5725	0.7852	0.7877
		STD	0.0256	0.0452	0.0299	0.0494	0.0415	0.0610	0.0400	0.0299
	Labels	Average	0.9416	0.9407	0.8673	0.8666	0.8764	0.7384	0.8692	0.8673
		STD	0.0201	0.0271	0.0299	0.0673	0.0312	0.0541	0.0307	0.0299
		P value	0.0000	0.0007	0.0000	0.0064	0.0001	0.0000	0.0001	0.0000
**IKKα**	No labels	Average	0.8519	0.8389	0.7826	0.7970	0.7650	0.5711	0.7771	0.7826
		STD	0.0367	0.0422	0.0466	0.1010	0.0354	0.0928	0.0566	0.0466
	Labels	Average	0.9337	0.9214	0.8554	0.8708	0.8372	0.7131	0.8521	0.8554
		STD	0.0255	0.0338	0.0367	0.0667	0.0401	0.0756	0.0395	0.0367
		P value	0.0000	0.0003	0.0018	0.0866	0.0008	0.0024	0.0049	0.0018
**IKKβ**	No labels	Average	0.8565	0.8291	0.7757	0.7968	0.7579	0.5592	0.7728	0.7757
		STD	0.0191	0.0337	0.0308	0.0847	0.0502	0.0626	0.0398	0.0308

	Labels	Average	0.9442	0.9410	0.8739	0.8884	0.8614	0.7485	0.8741	0.8739
		STD	0.0095	0.0157	0.0169	0.0267	0.0282	0.0339	0.0150	0.0169
		P value	0.0000	0.0000	0.0000	0.0104	0.0001	0.0000	0.0000	0.0000
**IKKε**	No labels	Average	0.8719	0.8609	0.7940	0.7843	0.7982	0.5895	0.7901	0.7940
		STD	0.0240	0.0360	0.0341	0.0565	0.0419	0.0676	0.0406	0.0341
	Labels	Average	0.9276	0.9247	0.8500	0.8717	0.8405	0.7020	0.8545	0.8500
		STD	0.0241	0.0244	0.0365	0.0384	0.0494	0.0725	0.0293	0.0365
		P value	0.0001	0.0005	0.0035	0.0015	0.0661	0.0032	0.0013	0.0035
**JNK1**	No labels	Average	0.9040	0.8990	0.8357	0.8332	0.8449	0.6750	0.8368	0.8357
		STD	0.0272	0.0349	0.0324	0.0383	0.0651	0.0642	0.0318	0.0324
	Labels	Average	0.9499	0.9361	0.8818	0.9196	0.8481	0.7676	0.8814	0.8818
		STD	0.0137	0.0233	0.0178	0.0241	0.0411	0.0331	0.0191	0.0178
		P value	0.0005	0.0176	0.0022	0.0000	0.9042	0.0019	0.0027	0.0022
**KHS1**	No labels	Average	0.8514	0.8280	0.7708	0.7898	0.7620	0.5452	0.7737	0.7708
		STD	0.0183	0.0410	0.0178	0.0384	0.0461	0.0340	0.0175	0.0178
	Labels	Average	0.9401	0.9315	0.8639	0.8801	0.8523	0.7294	0.8647	0.8639
		STD	0.0185	0.0257	0.0305	0.0407	0.0440	0.0585	0.0275	0.0305
		P value	0.0000	0.0000	0.0000	0.0001	0.0005	0.0000	0.0000	0.0000
**MAP3K1**	No labels	Average	0.8680	0.8449	0.7827	0.8001	0.7549	0.5689	0.7746	0.7827
		STD	0.0235	0.0447	0.0283	0.0490	0.0676	0.0576	0.0423	0.0283
	Labels	Average	0.9182	0.9075	0.8327	0.8256	0.8339	0.6672	0.8274	0.8327
		STD	0.0270	0.0428	0.0328	0.0450	0.0684	0.0634	0.0376	0.0328
		P value	0.0005	0.0072	0.0028	0.2652	0.0239	0.0029	0.0120	0.0028
**MAPKAPK3**	No labels	Average	0.8773	0.8784	0.7985	0.7854	0.8164	0.5995	0.7979	0.7985
		STD	0.0179	0.0347	0.0260	0.0624	0.0509	0.0508	0.0344	0.0260
	Labels	Average	0.9192	0.9157	0.8466	0.8574	0.8380	0.6946	0.8460	0.8466
		STD	0.0245	0.0292	0.0367	0.0662	0.0440	0.0729	0.0428	0.0367
		P value	0.0007	0.0245	0.0054	0.0289	0.3481	0.0054	0.0174	0.0054
**MAPKAPK5**	No labels	Average	0.8106	0.7890	0.7292	0.7674	0.7066	0.4652	0.7326	0.7292
		STD	0.0445	0.0741	0.0411	0.0887	0.0521	0.0873	0.0529	0.0411

	Labels	Average	0.8845	0.8879	0.8104	0.8258	0.8149	0.6226	0.8180	0.8104
		STD	0.0395	0.0408	0.0596	0.0729	0.0758	0.1189	0.0602	0.0596
		P value	0.0016	0.0035	0.0039	0.1450	0.0028	0.0054	0.0051	0.0039
**MARK2**	No labels	Average	0.8486	0.8293	0.7648	0.7531	0.7778	0.5299	0.7635	0.7648
		STD	0.0402	0.0581	0.0467	0.0729	0.0588	0.0955	0.0546	0.0467
	Labels	Average	0.9363	0.9300	0.8648	0.8841	0.8453	0.7316	0.8617	0.8648
		STD	0.0199	0.0234	0.0236	0.0456	0.0557	0.0442	0.0215	0.0236
		P value	0.0001	0.0004	0.0001	0.0004	0.0223	0.0001	0.0003	0.0001
**MARK4**	No labels	Average	0.8425	0.8266	0.7645	0.7587	0.7700	0.5270	0.7635	0.7645
		STD	0.0366	0.0539	0.0365	0.0380	0.0541	0.0756	0.0385	0.0365
	Labels	Average	0.9062	0.8895	0.8255	0.8469	0.8082	0.6573	0.8232	0.8255
		STD	0.0346	0.0477	0.0383	0.0595	0.0711	0.0733	0.0365	0.0383
		P value	0.0013	0.0176	0.0028	0.0019	0.2172	0.0016	0.0033	0.0028
**MINK**	No labels	Average	0.8495	0.8345	0.7732	0.7726	0.7880	0.5488	0.7784	0.7732
		STD	0.0327	0.0577	0.0252	0.0497	0.0470	0.0495	0.0299	0.0252
	Labels	Average	0.9415	0.9391	0.8631	0.8615	0.8595	0.7263	0.8600	0.8631
		STD	0.0230	0.0252	0.0394	0.0515	0.0383	0.0786	0.0404	0.0394
		P value	0.0000	0.0003	0.0000	0.0015	0.0025	0.0000	0.0002	0.0000
**MLK1**	No labels	Average	0.8655	0.8473	0.7880	0.7818	0.7861	0.5781	0.7820	0.7880
		STD	0.0208	0.0356	0.0254	0.0542	0.0405	0.0503	0.0288	0.0254
	Labels	Average	0.9446	0.9400	0.8770	0.8845	0.8659	0.7553	0.8743	0.8770
		STD	0.0186	0.0178	0.0310	0.0532	0.0261	0.0622	0.0328	0.0310
		P value	0.0000	0.0000	0.0000	0.0007	0.0002	0.0000	0.0000	0.0000
**MLK2**	No labels	Average	0.8853	0.8768	0.8009	0.8502	0.7698	0.6041	0.8065	0.8009
		STD	0.0196	0.0268	0.0376	0.0483	0.0660	0.0712	0.0477	0.0376
	Labels	Average	0.9453	0.9506	0.8698	0.8827	0.8806	0.7409	0.8794	0.8698
		STD	0.0166	0.0193	0.0352	0.0425	0.0639	0.0696	0.0329	0.0352
		P value	0.0000	0.0000	0.0008	0.1473	0.0020	0.0006	0.0016	0.0008
**MLK3**	No labels	Average	0.8329	0.8089	0.7563	0.7458	0.7745	0.5141	0.7569	0.7563
		STD	0.0345	0.0512	0.0414	0.0842	0.0464	0.0797	0.0530	0.0414

	Labels	Average	0.9155	0.9116	0.8373	0.8343	0.8349	0.6758	0.8328	0.8373
		STD	0.0200	0.0252	0.0257	0.0504	0.0487	0.0508	0.0312	0.0257
		P value	0.0000	0.0001	0.0002	0.0165	0.0148	0.0001	0.0022	0.0002
**MSK1**	No labels	Average	0.8951	0.8925	0.8237	0.7979	0.8378	0.6498	0.8134	0.8237
		STD	0.0315	0.0446	0.0403	0.0781	0.0655	0.0832	0.0459	0.0403
	Labels	Average	0.9335	0.9329	0.8535	0.8398	0.8684	0.7092	0.8523	0.8535
		STD	0.0191	0.0278	0.0204	0.0367	0.0458	0.0409	0.0194	0.0204
		P value	0.0070	0.0356	0.0686	0.1693	0.2672	0.0765	0.0369	0.0686
**MST1**	No labels	Average	0.8858	0.8665	0.8101	0.8249	0.8026	0.6216	0.8122	0.8101
		STD	0.0304	0.0406	0.0274	0.0465	0.0449	0.0544	0.0313	0.0274
	Labels	Average	0.9536	0.9538	0.8799	0.8756	0.8874	0.7606	0.8804	0.8799
		STD	0.0122	0.0141	0.0227	0.0376	0.0358	0.0460	0.0206	0.0227
		P value	0.0000	0.0001	0.0000	0.0209	0.0004	0.0000	0.0001	0.0000
**MST2**	No labels	Average	0.8727	0.8411	0.7856	0.7971	0.7691	0.5718	0.7808	0.7856
		STD	0.0203	0.0364	0.0306	0.0699	0.0446	0.0632	0.0421	0.0306
	Labels	Average	0.9470	0.9459	0.8673	0.8760	0.8572	0.7354	0.8657	0.8673
		STD	0.0115	0.0138	0.0209	0.0359	0.0298	0.0406	0.0215	0.0209
		P value	0.0000	0.0000	0.0000	0.0097	0.0002	0.0000	0.0001	0.0000
**MST3**	No labels	Average	0.8925	0.8760	0.8119	0.8221	0.8034	0.6234	0.8116	0.8119
		STD	0.0202	0.0302	0.0260	0.0555	0.0268	0.0532	0.0311	0.0260
	Labels	Average	0.9558	0.9490	0.8863	0.9046	0.8735	0.7743	0.8877	0.8863
		STD	0.0107	0.0229	0.0266	0.0371	0.0480	0.0533	0.0306	0.0266
		P value	0.0000	0.0000	0.0000	0.0020	0.0018	0.0000	0.0001	0.0000
**MST4**	No labels	Average	0.8842	0.8712	0.8098	0.8365	0.7917	0.6209	0.8124	0.8098
		STD	0.0249	0.0383	0.0351	0.0264	0.0608	0.0677	0.0381	0.0351
	Labels	Average	0.9218	0.9208	0.8434	0.8634	0.8312	0.6870	0.8466	0.8434
		STD	0.0318	0.0309	0.0334	0.0309	0.0376	0.0673	0.0286	0.0334
		P value	0.0122	0.0076	0.0523	0.0627	0.1177	0.0524	0.0467	0.0523
**NEK1**	No labels	Average	0.8532	0.8505	0.7599	0.7892	0.7498	0.5226	0.7665	0.7599
		STD	0.0301	0.0372	0.0381	0.0609	0.0650	0.0711	0.0473	0.0381

	Labels	Average	0.9289	0.9333	0.8472	0.8482	0.8538	0.6940	0.8493	0.8472
		STD	0.0150	0.0191	0.0223	0.0590	0.0326	0.0450	0.0298	0.0223
		P value	0.0000	0.0000	0.0000	0.0516	0.0008	0.0000	0.0005	0.0000
**NEK2**	No labels	Average	0.8489	0.8246	0.7695	0.8187	0.7519	0.5471	0.7801	0.7695
		STD	0.0292	0.0494	0.0237	0.0664	0.0519	0.0509	0.0221	0.0237
	Labels	Average	0.9383	0.9362	0.8715	0.8766	0.8690	0.7433	0.8715	0.8715
		STD	0.0207	0.0282	0.0273	0.0351	0.0469	0.0559	0.0261	0.0273
		P value	0.0000	0.0000	0.0000	0.0369	0.0001	0.0000	0.0000	0.0000
**NEK4**	No labels	Average	0.8479	0.8070	0.7667	0.8013	0.7499	0.5355	0.7731	0.7667
		STD	0.0295	0.0603	0.0259	0.0597	0.0389	0.0544	0.0335	0.0259
	Labels	Average	0.9494	0.9497	0.8761	0.8830	0.8729	0.7547	0.8765	0.8761
		STD	0.0144	0.0150	0.0278	0.0528	0.0370	0.0548	0.0287	0.0278
		P value	0.0000	0.0000	0.0000	0.0066	0.0000	0.0000	0.0000	0.0000
**NEK6**	No labels	Average	0.8718	0.8614	0.8000	0.8223	0.7941	0.6028	0.8063	0.8000
		STD	0.0178	0.0330	0.0239	0.0517	0.0357	0.0483	0.0257	0.0239
	Labels	Average	0.9309	0.9287	0.8576	0.8727	0.8476	0.7160	0.8590	0.8576
		STD	0.0170	0.0231	0.0274	0.0438	0.0379	0.0540	0.0292	0.0274
		P value	0.0000	0.0001	0.0002	0.0389	0.0065	0.0002	0.0008	0.0002
**NEK7**	No labels	Average	0.8790	0.8562	0.7876	0.8031	0.7703	0.5776	0.7844	0.7876
		STD	0.0115	0.0273	0.0153	0.0606	0.0275	0.0346	0.0244	0.0153
	Labels	Average	0.9214	0.9158	0.8444	0.8587	0.8316	0.6889	0.8442	0.8444
		STD	0.0264	0.0315	0.0263	0.0465	0.0251	0.0543	0.0274	0.0263
		P value	0.0008	0.0005	0.0001	0.0436	0.0001	0.0001	0.0001	0.0001
**NEK9**	No labels	Average	0.8665	0.8406	0.7963	0.8228	0.7546	0.5943	0.7848	0.7963
		STD	0.0336	0.0531	0.0301	0.0605	0.0611	0.0610	0.0418	0.0301
	Labels	Average	0.9423	0.9369	0.8713	0.8642	0.8700	0.7431	0.8654	0.8713
		STD	0.0193	0.0178	0.0250	0.0454	0.0380	0.0520	0.0189	0.0250
		P value	0.0000	0.0003	0.0000	0.1201	0.0002	0.0000	0.0002	0.0000
**NIM1**	No labels	Average	0.8576	0.8536	0.7733	0.7803	0.7780	0.5513	0.7764	0.7733
		STD	0.0450	0.0423	0.0552	0.0915	0.0444	0.1083	0.0571	0.0552

	Labels	Average	0.9063	0.8926	0.8305	0.8764	0.8115	0.6677	0.8403	0.8305
		STD	0.0270	0.0427	0.0204	0.0567	0.0413	0.0407	0.0202	0.0204
		P value	0.0141	0.0668	0.0134	0.0172	0.1146	0.0111	0.0088	0.0134
**NLK**	No labels	Average	0.8582	0.8309	0.7756	0.7539	0.7646	0.5544	0.7545	0.7756
		STD	0.0363	0.0628	0.0449	0.0770	0.0870	0.0912	0.0555	0.0449
	Labels	Average	0.9261	0.9176	0.8444	0.8676	0.8259	0.6931	0.8438	0.8444
		STD	0.0183	0.0199	0.0268	0.0564	0.0427	0.0529	0.0226	0.0268
		P value	0.0002	0.0024	0.0013	0.0024	0.0800	0.0014	0.0008	0.0013
**PAK1**	No labels	Average	0.9184	0.9181	0.8207	0.8324	0.8233	0.6530	0.8207	0.8207
		STD	0.0237	0.0243	0.0358	0.0847	0.0787	0.0524	0.0360	0.0358
	Labels	Average	0.9335	0.9337	0.8565	0.8371	0.8783	0.7171	0.8551	0.8565
		STD	0.0327	0.0370	0.0256	0.0546	0.0415	0.0494	0.0262	0.0256
		P value	0.2800	0.3037	0.0263	0.8905	0.0853	0.0155	0.0337	0.0263
**PAK2**	No labels	Average	0.8856	0.8670	0.8051	0.7974	0.7975	0.6107	0.7949	0.8051
		STD	0.0288	0.0478	0.0501	0.0726	0.0722	0.0982	0.0583	0.0501
	Labels	Average	0.9351	0.9274	0.8500	0.8524	0.8349	0.7006	0.8419	0.8500
		STD	0.0220	0.0267	0.0401	0.0570	0.0600	0.0800	0.0448	0.0401
		P value	0.0008	0.0051	0.0508	0.0915	0.2478	0.0477	0.0722	0.0508
**PAK3**	No labels	Average	0.8996	0.8990	0.7976	0.7916	0.8043	0.5921	0.7972	0.7976
		STD	0.0210	0.0277	0.0248	0.0395	0.0444	0.0489	0.0346	0.0248
	Labels	Average	0.9306	0.9280	0.8532	0.8602	0.8479	0.7078	0.8529	0.8532
		STD	0.0266	0.0288	0.0344	0.0571	0.0385	0.0704	0.0362	0.0344
		P value	0.0139	0.0432	0.0011	0.0092	0.0394	0.0009	0.0037	0.0011
**PHKG2**	No labels	Average	0.8648	0.8361	0.7779	0.7702	0.7637	0.5529	0.7657	0.7779
		STD	0.0368	0.0592	0.0395	0.0441	0.0674	0.0809	0.0475	0.0395
	Labels	Average	0.9362	0.9326	0.8654	0.8866	0.8524	0.7338	0.8674	0.8654
		STD	0.0277	0.0386	0.0325	0.0626	0.0447	0.0630	0.0386	0.0325
		P value	0.0002	0.0009	0.0001	0.0003	0.0048	0.0001	0.0001	0.0001
**PIM1**	No labels	Average	0.8863	0.8642	0.8048	0.8207	0.7963	0.6102	0.8060	0.8048
		STD	0.0310	0.0493	0.0404	0.0482	0.0730	0.0810	0.0471	0.0404

	Labels	Average	0.9347	0.9265	0.8587	0.8733	0.8536	0.7184	0.8623	0.8587
		STD	0.0242	0.0439	0.0352	0.0353	0.0568	0.0690	0.0367	0.0352
		P value	0.0018	0.0112	0.0075	0.0176	0.0803	0.0071	0.0117	0.0075
**PIM3**	No labels	Average	0.8731	0.8525	0.7862	0.8517	0.7481	0.5801	0.7943	0.7862
		STD	0.0366	0.0608	0.0472	0.0552	0.0671	0.0918	0.0475	0.0472
	Labels	Average	0.9285	0.9276	0.8500	0.8742	0.8431	0.7020	0.8569	0.8500
		STD	0.0340	0.0348	0.0467	0.0486	0.0598	0.0925	0.0429	0.0467
		P value	0.0038	0.0060	0.0099	0.3718	0.0054	0.0117	0.0089	0.0099
**PKA Cα**	No labels	Average	0.9192	0.9095	0.8407	0.8463	0.8442	0.6831	0.8435	0.8407
		STD	0.0302	0.0355	0.0387	0.0546	0.0533	0.0765	0.0387	0.0387
	Labels	Average	0.9316	0.9188	0.8626	0.8741	0.8590	0.7276	0.8646	0.8626
		STD	0.0190	0.0342	0.0276	0.0483	0.0547	0.0529	0.0346	0.0276
		P value	0.3120	0.5801	0.1843	0.2668	0.5687	0.1701	0.2375	0.1843
**PLK1**	No labels	Average	0.9109	0.9067	0.8398	0.8502	0.8456	0.6814	0.8455	0.8398
		STD	0.0311	0.0429	0.0299	0.0553	0.0594	0.0614	0.0355	0.0299
	Labels	Average	0.9581	0.9483	0.8935	0.9128	0.8776	0.7881	0.8940	0.8935
		STD	0.0154	0.0194	0.0313	0.0370	0.0441	0.0624	0.0314	0.0313
		P value	0.0013	0.0205	0.0016	0.0124	0.2120	0.0018	0.0066	0.0016
**PLK3**	No labels	Average	0.9159	0.9154	0.8333	0.8317	0.8458	0.6653	0.8375	0.8333
		STD	0.0303	0.0299	0.0401	0.0669	0.0369	0.0789	0.0449	0.0401
	Labels	Average	0.9574	0.9442	0.9000	0.9334	0.8706	0.8027	0.8999	0.9000
		STD	0.0179	0.0361	0.0215	0.0395	0.0342	0.0430	0.0227	0.0215
		P value	0.0031	0.0825	0.0006	0.0014	0.1573	0.0004	0.0025	0.0006
**QIK**	No labels	Average	0.8286	0.8240	0.7562	0.7804	0.7571	0.5122	0.7670	0.7562
		STD	0.0222	0.0292	0.0317	0.0478	0.0436	0.0666	0.0296	0.0317
	Labels	Average	0.8982	0.8821	0.8226	0.8549	0.8019	0.6484	0.8260	0.8226
		STD	0.0126	0.0349	0.0244	0.0466	0.0361	0.0479	0.0212	0.0244
		P value	0.0000	0.0013	0.0001	0.0036	0.0289	0.0001	0.0002	0.0001
**RSK3**	No labels	Average	0.8622	0.8573	0.7693	0.7986	0.7628	0.5433	0.7772	0.7693
		STD	0.0321	0.0451	0.0427	0.0629	0.0677	0.0813	0.0450	0.0427

	Labels	Average	0.9140	0.9062	0.8336	0.8516	0.8210	0.6676	0.8347	0.8336
		STD	0.0190	0.0309	0.0313	0.0432	0.0518	0.0618	0.0354	0.0313
		P value	0.0009	0.0164	0.0021	0.0535	0.0565	0.0020	0.0078	0.0021
**SGK3**	No labels	Average	0.8801	0.8625	0.7990	0.8338	0.7675	0.6048	0.7963	0.7990
		STD	0.0244	0.0406	0.0351	0.0692	0.0635	0.0728	0.0441	0.0351
	Labels	Average	0.9330	0.9288	0.8519	0.8400	0.8669	0.7062	0.8511	0.8519
		STD	0.0317	0.0364	0.0385	0.0429	0.0664	0.0784	0.0359	0.0385
		P value	0.0010	0.0018	0.0070	0.8238	0.0045	0.0109	0.0100	0.0070
**SIK**	No labels	Average	0.8601	0.8503	0.7695	0.7584	0.7695	0.5447	0.7596	0.7695
		STD	0.0309	0.0323	0.0321	0.0838	0.0475	0.0614	0.0376	0.0321
	Labels	Average	0.8943	0.8820	0.8158	0.8315	0.8137	0.6344	0.8198	0.8158
		STD	0.0365	0.0283	0.0512	0.0775	0.0379	0.0991	0.0418	0.0512
		P value	0.0463	0.0397	0.0361	0.0708	0.0434	0.0357	0.0049	0.0361
**TAK1**	No labels	Average	0.8779	0.9014	0.7823	0.7789	0.8111	0.5631	0.7924	0.7823
		STD	0.0429	0.0285	0.0383	0.0713	0.0394	0.0844	0.0366	0.0383
	Labels	Average	0.9332	0.9133	0.8532	0.8662	0.8499	0.7146	0.8526	0.8532
		STD	0.0254	0.0549	0.0522	0.0985	0.0664	0.0982	0.0552	0.0522
		P value	0.0047	0.5755	0.0045	0.0466	0.1532	0.0026	0.0151	0.0045
**TAO2**	No labels	Average	0.8511	0.8316	0.7693	0.7817	0.7714	0.5408	0.7746	0.7693
		STD	0.0340	0.0611	0.0466	0.0553	0.0727	0.0919	0.0525	0.0466
	Labels	Average	0.9414	0.9390	0.8669	0.8681	0.8659	0.7352	0.8654	0.8669
		STD	0.0205	0.0180	0.0365	0.0593	0.0411	0.0714	0.0369	0.0365
		P value	0.0000	0.0004	0.0001	0.0050	0.0043	0.0001	0.0006	0.0001
**TLK1**	No labels	Average	0.9137	0.8937	0.8214	0.7929	0.8476	0.6481	0.8154	0.8214
		STD	0.0300	0.0485	0.0300	0.0643	0.0727	0.0612	0.0404	0.0300
	Labels	Average	0.9353	0.9215	0.8643	0.8631	0.8673	0.7271	0.8637	0.8643
		STD	0.0344	0.0421	0.0365	0.0608	0.0418	0.0753	0.0378	0.0365
		P value	0.1739	0.2106	0.0144	0.0286	0.4905	0.0257	0.0173	0.0144
**TNIK**	No labels	Average	0.8711	0.8445	0.8000	0.8250	0.7723	0.6035	0.7959	0.8000
		STD	0.0396	0.0494	0.0332	0.0712	0.0373	0.0691	0.0396	0.0332

	Labels	Average	0.9591	0.9547	0.8936	0.8791	0.9021	0.7870	0.8897	0.8936
		STD	0.0154	0.0250	0.0220	0.0337	0.0392	0.0446	0.0271	0.0220
		P value	0.0001	0.0000	0.0000	0.0604	0.0000	0.0000	0.0000	0.0000
**TSSK1**	No labels	Average	0.8705	0.8472	0.7941	0.8023	0.7934	0.5948	0.7937	0.7941
		STD	0.0326	0.0606	0.0423	0.0873	0.0661	0.0847	0.0511	0.0423
	Labels	Average	0.8838	0.8737	0.8049	0.7820	0.8400	0.6157	0.8068	0.8049
		STD	0.0343	0.0426	0.0373	0.0724	0.0520	0.0695	0.0414	0.0373
		P value	0.4084	0.2979	0.5734	0.5973	0.1144	0.5735	0.5574	0.5734
**TTBK1**	No labels	Average	0.8807	0.8607	0.8094	0.8399	0.7882	0.6224	0.8118	0.8094
		STD	0.0142	0.0258	0.0219	0.0490	0.0286	0.0441	0.0219	0.0219
	Labels	Average	0.9620	0.9577	0.8981	0.9110	0.8857	0.7967	0.8977	0.8981
		STD	0.0111	0.0190	0.0168	0.0298	0.0273	0.0341	0.0173	0.0168
		P value	0.0000	0.0000	0.0000	0.0021	0.0000	0.0000	0.0000	0.0000
**YSK1**	No labels	Average	0.8873	0.8684	0.7993	0.8274	0.7819	0.6034	0.8018	0.7993
		STD	0.0348	0.0727	0.0423	0.0643	0.0686	0.0858	0.0508	0.0423
	Labels	Average	0.9504	0.9521	0.8787	0.9016	0.8671	0.7574	0.8835	0.8787
		STD	0.0196	0.0194	0.0255	0.0318	0.0389	0.0516	0.0278	0.0255
		P value	0.0003	0.0072	0.0002	0.0083	0.0058	0.0004	0.0008	0.0002
**ZAK**	No labels	Average	0.8985	0.8756	0.8234	0.8299	0.8181	0.6516	0.8210	0.8234
		STD	0.0253	0.0452	0.0340	0.0464	0.0737	0.0669	0.0379	0.0340
	Labels	Average	0.9579	0.9471	0.8928	0.9092	0.8786	0.7893	0.8915	0.8928
		STD	0.0222	0.0414	0.0341	0.0535	0.0583	0.0655	0.0382	0.0341
		P value	0.0001	0.0026	0.0004	0.0036	0.0703	0.0003	0.0010	0.0004
**p38α**	No labels	Average	0.8764	0.8602	0.7906	0.7677	0.8069	0.5825	0.7846	0.7906
		STD	0.0274	0.0514	0.0271	0.0580	0.0606	0.0544	0.0423	0.0271
	Labels	Average	0.9362	0.9319	0.8724	0.8952	0.8658	0.7444	0.8796	0.8724
		STD	0.0188	0.0285	0.0317	0.0418	0.0355	0.0638	0.0312	0.0317
		P value	0.0001	0.0026	0.0000	0.0001	0.0242	0.0000	0.0001	0.0000
**p38β**	No labels	Average	0.8728	0.8695	0.8044	0.8160	0.8079	0.6115	0.8092	0.8044
		STD	0.0331	0.0565	0.0380	0.0706	0.0671	0.0813	0.0470	0.0380

	Labels	Average	0.9323	0.9241	0.8626	0.8834	0.8467	0.7299	0.8616	0.8626
		STD	0.0202	0.0195	0.0204	0.0637	0.0475	0.0448	0.0212	0.0204
		P value	0.0003	0.0192	0.0012	0.0476	0.1763	0.0018	0.0097	0.0012
**p38δ**	No labels	Average	0.9001	0.8736	0.8269	0.8224	0.8181	0.6523	0.8192	0.8269
		STD	0.0161	0.0293	0.0215	0.0365	0.0336	0.0416	0.0200	0.0215
	Labels	Average	0.9413	0.9323	0.8731	0.8740	0.8712	0.7471	0.8713	0.8731
		STD	0.0238	0.0268	0.0295	0.0468	0.0467	0.0592	0.0328	0.0295
		P value	0.0006	0.0003	0.0015	0.0182	0.0135	0.0012	0.0010	0.0015
**p38γ**	No labels	Average	0.9036	0.8966	0.8379	0.8269	0.8432	0.6744	0.8341	0.8379
		STD	0.0204	0.0303	0.0288	0.0440	0.0289	0.0552	0.0260	0.0288
	Labels	Average	0.9440	0.9421	0.8697	0.8818	0.8638	0.7380	0.8709	0.8697
		STD	0.0226	0.0335	0.0374	0.0530	0.0572	0.0710	0.0407	0.0374
		P value	0.0009	0.0074	0.0592	0.0283	0.3518	0.0491	0.0367	0.0592
**p70S6K**	No labels	Average	0.9115	0.8939	0.8268	0.8094	0.8248	0.6541	0.8138	0.8268
		STD	0.0351	0.0421	0.0456	0.0868	0.0547	0.0900	0.0535	0.0456
	Labels	Average	0.9488	0.9412	0.8928	0.8926	0.8944	0.7858	0.8924	0.8928
		STD	0.0188	0.0264	0.0361	0.0409	0.0536	0.0711	0.0368	0.0361
		P value	0.0140	0.0119	0.0034	0.0221	0.0138	0.0031	0.0023	0.0034

S10. Additional Cross-Model Figures

S10.1. O-GlcNAc Prediction with Phosphorylation (S,T) Sites Model Results

PTM			AUC	AUPRC	Accuracy	Recall	Precision	MCC	F1	Specificity
**O-GlcNAc Atlas, Human-only**	No labels	Avg	0.8099	0.8161	0.7267	0.6940	0.7474	0.4563	0.7186	0.7267
		STD	0.0158	0.0167	0.0136	0.0363	0.0280	0.0270	0.0159	0.0136
	GlcNAc Labels	Avg	0.9122	0.9235	0.8281	0.7668	0.8717	0.6612	0.8154	0.8281
		STD	0.0037	0.0046	0.0064	0.0221	0.0226	0.0135	0.0084	0.0064
		P value	8.63E−09	8.73E−09	6.25E−09	9.31E−05	8.51E−07	4.96E−09	1.53E−08	6.25E−09
	Phosphorylation (S,T) Labels	Avg	0.8157	0.8194	0.7322	0.6919	0.7549	0.4667	0.7215	0.7322
		STD	0.0098	0.0131	0.0097	0.0350	0.0113	0.0183	0.0173	0.0097
		P value	0.3422	0.6227	0.3310	0.8972	0.4463	0.3340	0.7042	0.3310
**O-GlcNAc Atlas, All-organism**	No labels	Average	0.766	0.767	0.692	0.66	0.705	0.386	0.68	0.692
		STD	0.01	0.012	0.009	0.043	0.023	0.018	0.015	0.009
	O-GlcNAc Labels	Average	0.879	0.89	0.796	0.762	0.818	0.595	0.789	0.796
		STD	0.006	0.007	0.008	0.021	0.016	0.016	0.01	0.008
		P-value	1.88E−12	5.31E−10	2.74E−11	1.32E−05	5.64E−08	3.22E−11	1.67E−08	2.74E−11
	Phosphorylation (S,T) labels	Average	0.783	0.781	0.705	0.667	0.723	0.412	0.693	0.705
		STD	0.011	0.009	0.013	0.035	0.016	0.024	0.018	0.013
		P value	0.001	0.008	0.017	0.719	0.051	0.013	0.101	0.017

S10.2. Human O-GlcNAc Prediction with Phosphorylation (S,T) Sites Frequency and ROC Graphs

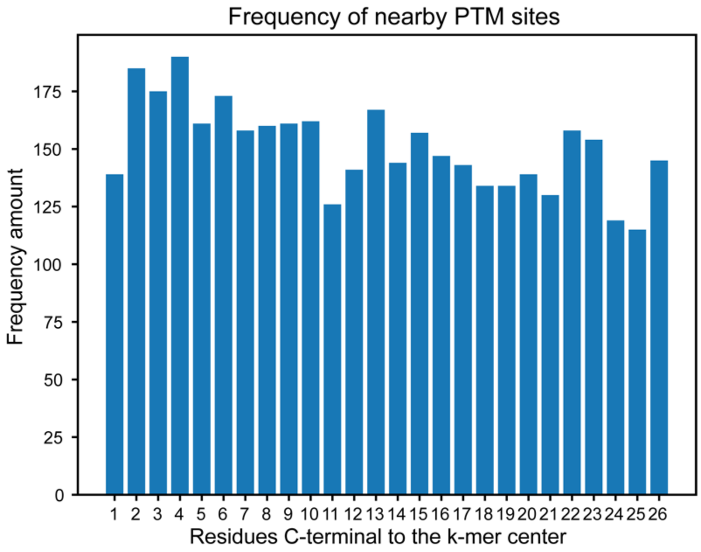

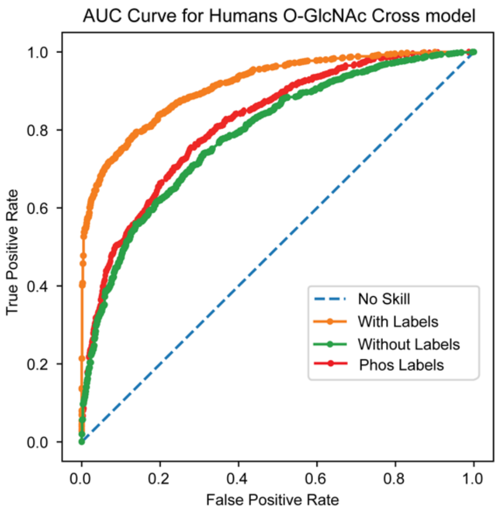

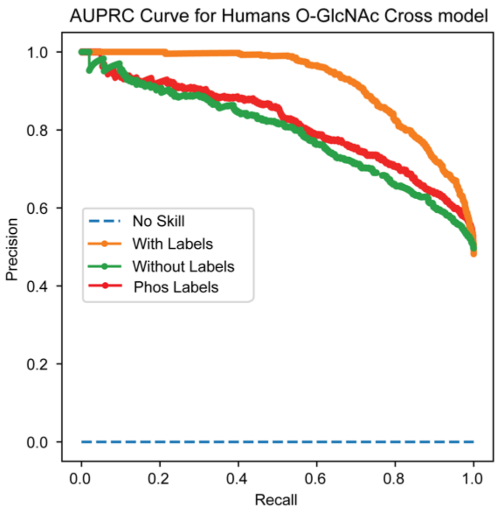

S11. Effect of Homology on Model Performance

S11.1. Evaluation of Sequence Identity

To accurately evaluate the performance of a sequence-based deep learning model, it is important to consider sequence similarity between the training data and independent test sets. Given that we are evaluating enzymatic PTMs, some level of sequence similarity is expected in some cases because enzymes typically recognize particular sequences. For every model, we ensured that no k-mers present in the training and validation data were present in the test data (*i.e.,* a sequence similarity cutoff of 100%). To further evaluate how similarities impact our datasets, we used CD-Hit (Fu *et al.* 2012) to remove sequences from the test sets that shared different levels of sequence similarity down to a cutoff of 40% sequence identity and plotted the AUC values of each cutoff. In these plots, we saw that there was often an elbow at a cutoff of around 80% so we included all the results at this similarity level. The results of this evaluation are shown in Supplementary Fig. 11.2–11.5. This evaluation was not done for kinase-specific datasets because they are small datasets already and because particular kinases likely have specific recognition sites that might require high sequence identity, and we want our models to capture that.

For some modifications, specifically the hydroxylation of lysine and proline, there were no instances of sequences below a certain identity cutoff, which indicates that proteins containing these modifications are likely to be homologous. (That homology makes sense because the proteins having these PTMs are often collagens.) For most modifications, we saw a slight decrease in performance as we decreased sequence identity. More interestingly, the labeled model performance decreased more than did the unlabeled models. This dichotomy is likely due, in part, to a technical limitation, as k-mers with modified amino acids had to be converted to canonical amino acids for comparison using CD-Hit and then converted back. Inclusion of modified amino acids generally lowers the sequence identity, so those sets in our model likely have lower levels of sequence identity than do the unlabeled sets. Additionally, this decrease could also indicate that incorporating PTM labels allows a model to distinguish between high-identity sequences and would likely increase its performance in context-specific prediction (where some potential modification sites are modified whereas others are not). Regardless, labeled models that perform significantly better than their unlabeled counterpart generally maintain this advantage at lower levels of sequence identity though to a lesser extent.

Finally, it is important to note that sequence identity greatly depends on whether an analysis is performed at a protein or peptide level (*e.g.*, our 53 k-mer) and, in the latter case, on the length of the peptide fragment.

S11.2. Using CD-Hit to Lower Test-Set Homology in Musite Deep Datasets

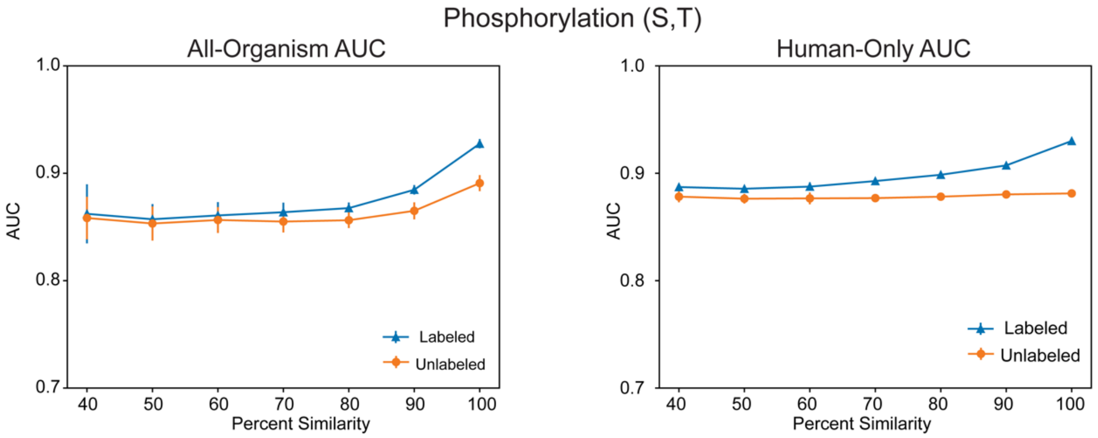

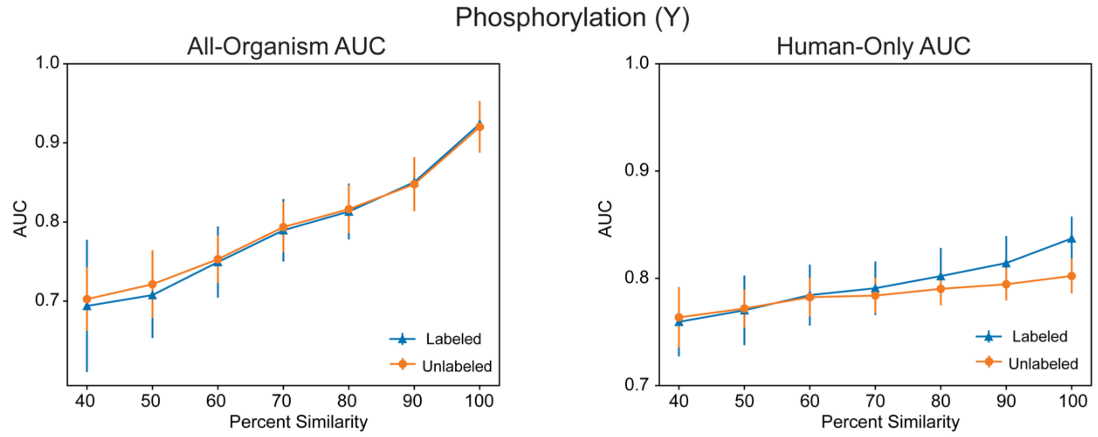

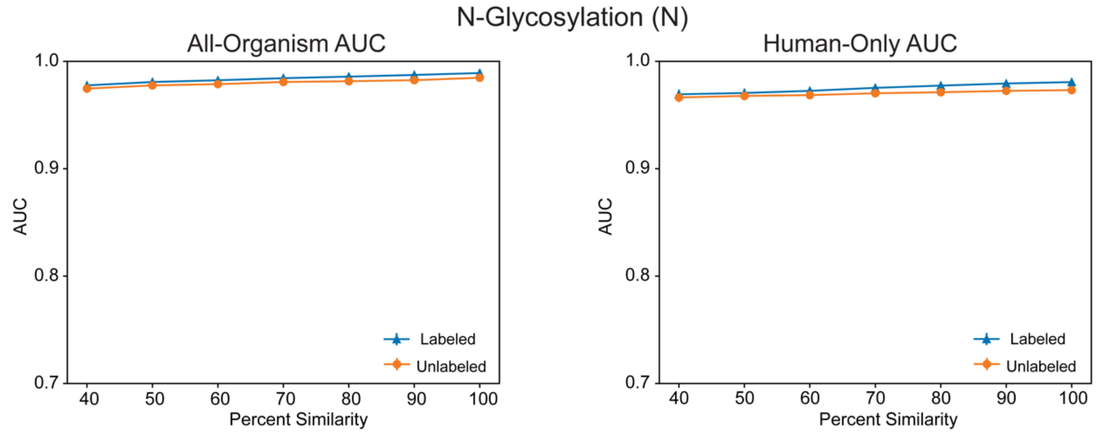

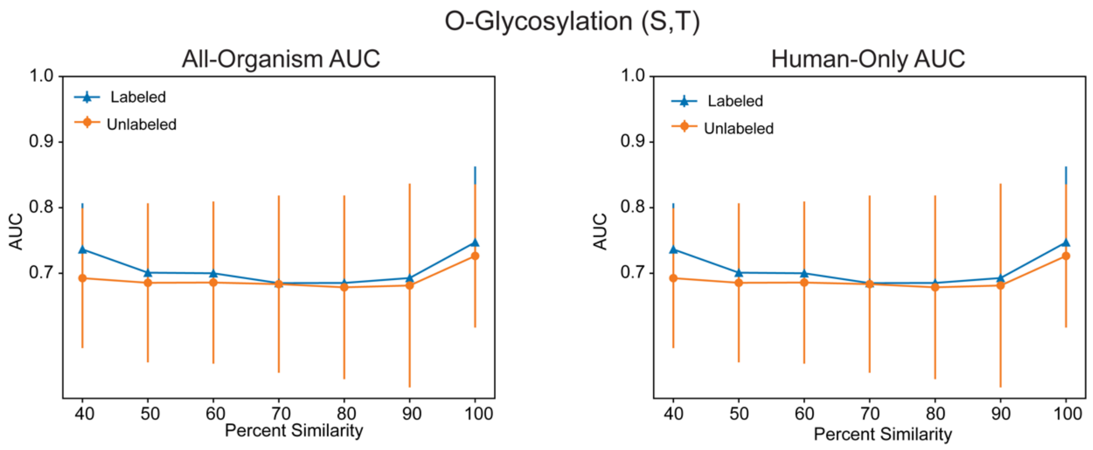
O-Glycosylation (S,T)
All-Organism AUC
Human-Only AUC
Labeled
Unlabeled
AUC
Percent Similarity

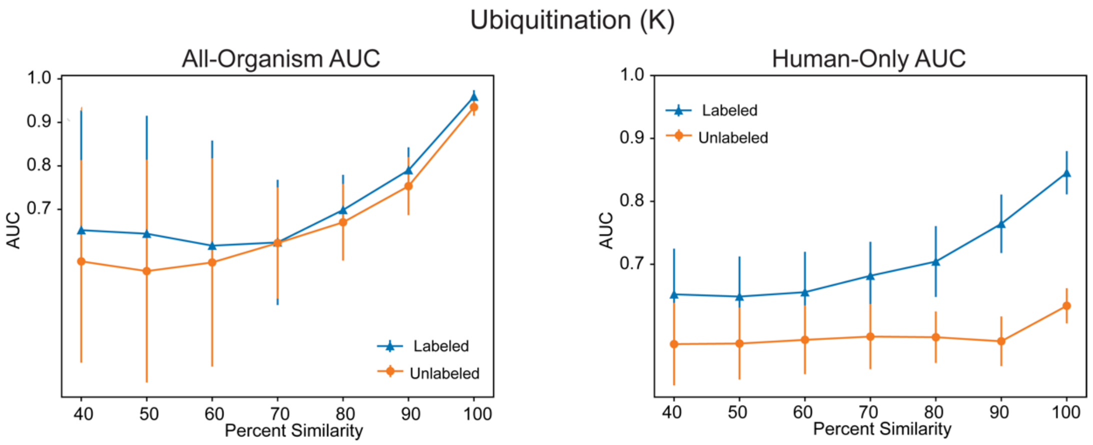
Ubiquitination (K)
All-Organism AUC
Human-Only AUC
Labeled
Unlabeled
AUC
Percent Similarity

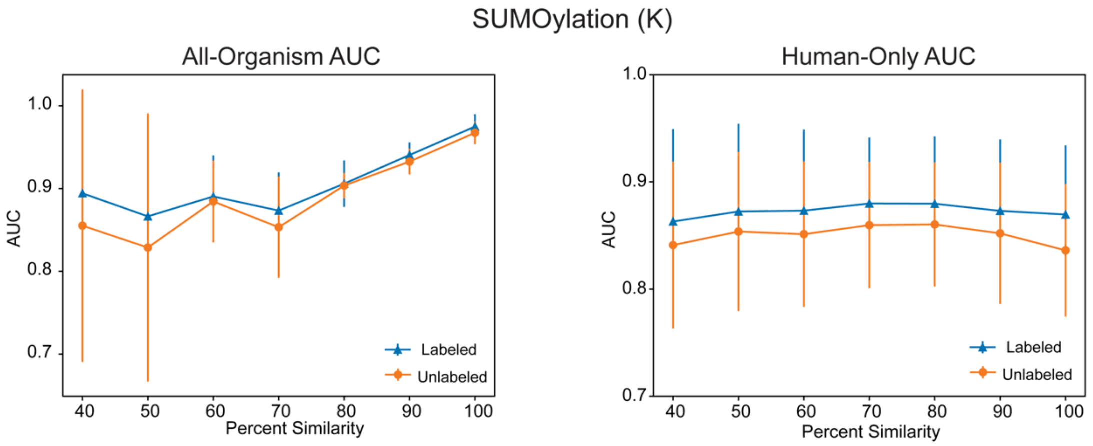
SUMOylation (K)
All-Organism AUC
Human-Only AUC
Labeled
Unlabeled
AUC
Percent Similarity

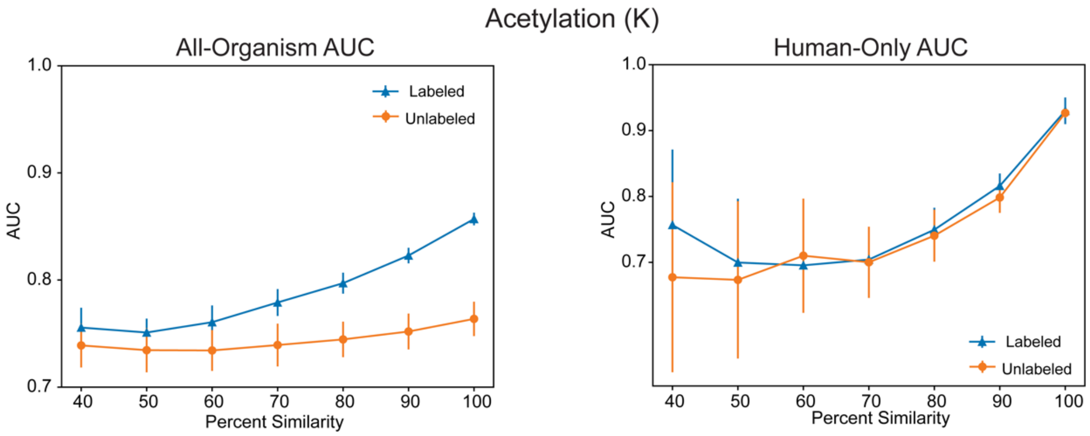
Acetylation (K)
All-Organism AUC
Human-Only AUC
Labeled
Unlabeled
AUC
Percent Similarity
1.0
0.9
0.8
0.7
40
50
60
70
80
90
100

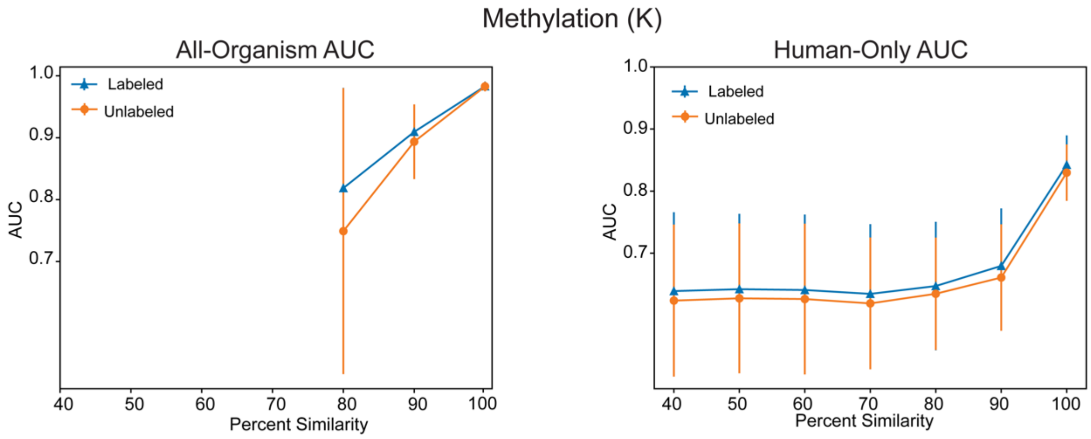
Methylation (K)
All-Organism AUC
Human-Only AUC
Labeled
Unlabeled
AUC
Percent Similarity
1.0
0.9
0.8
0.7
40
50
60
70
80
90
100

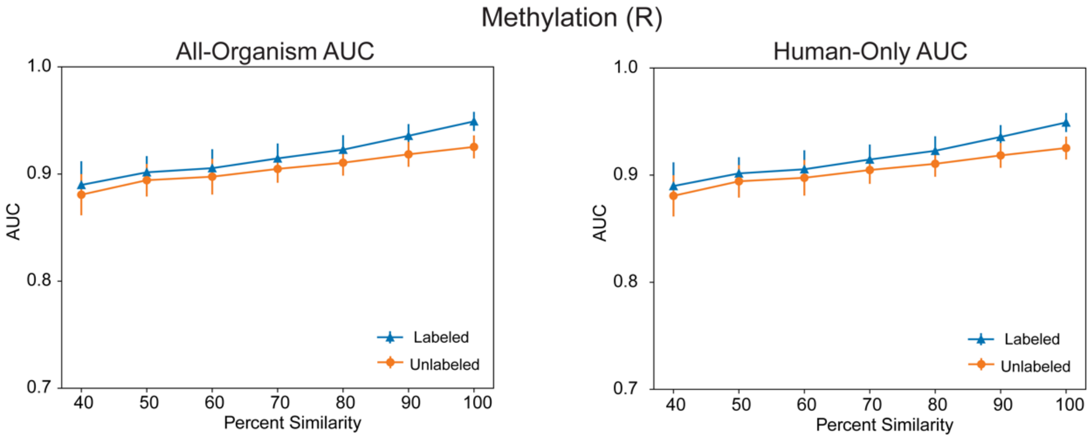
Methylation (R)
All-Organism AUC
Human-Only AUC
Labeled
Unlabeled
AUC
Percent Similarity
1.0
0.9
0.8
0.7
40
50
60
70
80
90
100

Pyroglutamylation (Q)

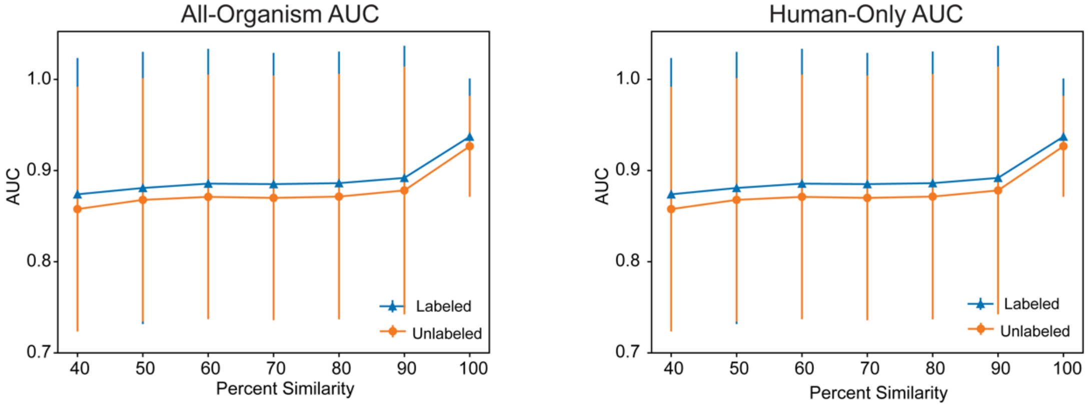

Palmitoylation (C)

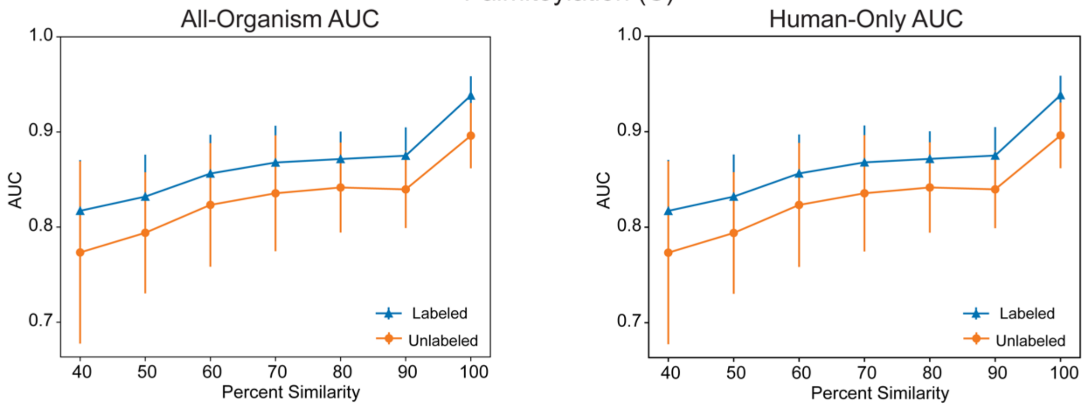

Hydroxylation (P)

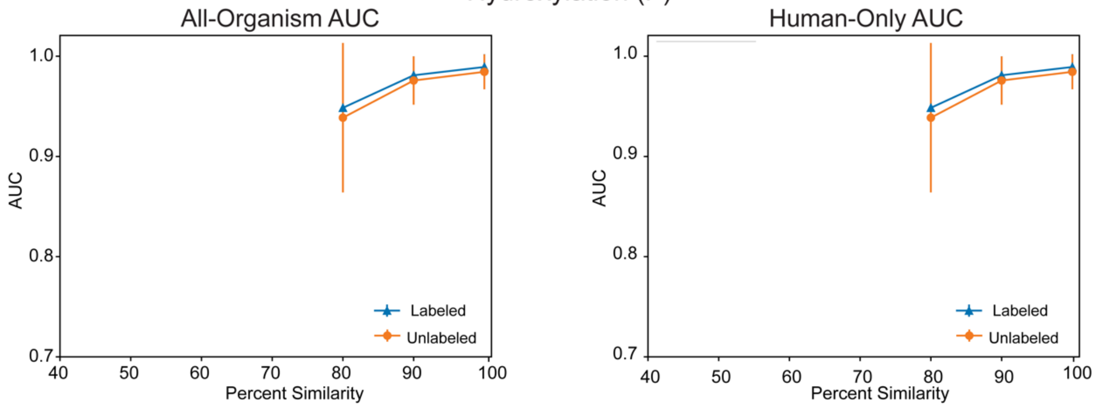

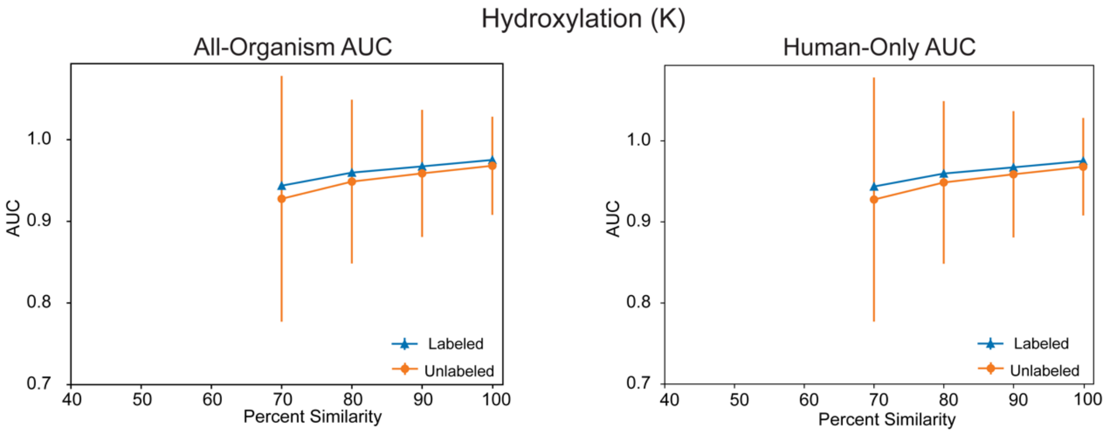
Hydroxylation (K)
All-Organism AUC
Human-Only AUC
AUC
0.7
0.8
0.9
1.0
40
50
60
70
80
90
100
Percent Similarity
Labeled
Unlabeled

Table S11.2: Model Results at 80% Sequence Similarity Cutoff for MusiteDeep Datasets

PTM	Labels	Stat	AUC	AUPRC	Accuracy	Recall	Precision	MCC	F1	Specificity
**Phosphorylation (S,T) All-organism**	No labels	Average	0.8563	0.5306	0.7919	0.7545	0.3806	0.4286	0.5052	0.7981
		STD	0.0066	0.0121	0.0138	0.0236	0.0211	0.0123	0.0143	0.0195
	Labels	Average	0.8675	0.5706	0.8503	0.6797	0.4780	0.4850	0.5609	0.8783
		STD	0.0048	0.0172	0.0073	0.0132	0.0216	0.0142	0.0142	0.0098
		P value	7.13E−04	3.03E−05	2.90E−08	8.47E−07	1.43E−08	5.21E−08	1.52E−07	4.84E−08
**Phosphorylation (S,T) Human-only**	No labels	Average	0.8782	0.7905	0.8016	0.7865	0.6886	0.5806	0.7339	0.8097
		STD	0.0038	0.0108	0.0079	0.0182	0.0197	0.0112	0.0070	0.0196
	Labels	Average	0.8986	0.8301	0.8345	0.7194	0.7870	0.6295	0.7513	0.8960
		STD	0.0027	0.0083	0.0034	0.0216	0.0156	0.0085	0.0082	0.0109
		P value	4.30E−10	1.10E−07	6.51E−08	1.40E−06	1.27E−09	9.79E−09	1.45E−04	1.44E−08
**Phosphorylation (Y) All-organism**	No labels	Average	0.8162	0.5144	0.7932	0.6620	0.4295	0.4032	0.5204	0.8034
		STD	0.0271	0.0602	0.0540	0.0837	0.0386	0.0246	0.0524	0.1144
	Labels	Average	0.8132	0.5289	0.7858	0.6728	0.4244	0.4014	0.5153	0.8030
		STD	0.0334	0.0785	0.0469	0.0628	0.0757	0.0424	0.0595	0.0711
		P value	0.8371	0.6648	0.7599	0.7613	0.8608	0.9166	0.8496	0.9945
**Phosphorylation (Y) Human-only**	No labels	Average	0.7902	0.7316	0.7164	0.7035	0.6635	0.4272	0.6815	0.7249
		STD	0.0138	0.0391	0.0186	0.0419	0.0397	0.0356	0.0248	0.0518
	Labels	Average	0.8021	0.7704	0.7310	0.6614	0.6997	0.4495	0.6780	0.7843
		STD	0.0250	0.0514	0.0220	0.0609	0.0517	0.0518	0.0445	0.0310
		P value	0.2325	0.0898	0.1459	0.1069	0.1142	0.3019	0.8437	0.0101
**N-Glycosylation (N) All-organism**	No labels	Average	0.9815	0.9527	0.9700	0.9958	0.9267	0.9331	0.9599	0.9503
		STD	0.0034	0.0153	0.0083	0.0015	0.0236	0.0056	0.0127	0.0055
	Labels	Average	0.9857	0.9666	0.9705	0.9944	0.9289	0.9338	0.9604	0.9518
		STD	0.0028	0.0121	0.0083	0.0031	0.0232	0.0061	0.0126	0.0062
		P value	0.0091	0.0472	0.9088	0.2315	0.8417	0.7905	0.9291	0.5910
**N-Glycosylation (N) Human-only**	No labels	Average	0.9712	0.9450	0.9418	0.9849	0.8962	0.8871	0.9384	0.9066
		STD	0.0034	0.0063	0.0038	0.0040	0.0084	0.0070	0.0039	0.0079

	Labels	Average	0.9774	0.9644	0.9424	0.9808	0.9001	0.8874	0.9387	0.9109
		STD	0.0026	0.0038	0.0038	0.0043	0.0079	0.0071	0.0041	0.0069
		P value	0.0005	0.0000	0.7649	0.0536	0.3222	0.9167	0.8795	0.2327
**O-Glycosylation (S,T) All-organism**	No labels	Average	0.8801	0.5794	0.8545	0.7767	0.3614	0.4489	0.4642	0.8687
		STD	0.1320	0.2525	0.0485	0.2043	0.1426	0.1334	0.1314	0.0498
	Labels	Average	0.9109	0.5468	0.8839	0.6983	0.4274	0.4848	0.5174	0.8968
		STD	0.0658	0.2853	0.0591	0.2872	0.2295	0.2581	0.2395	0.0569
		P value	0.5416	0.8007	0.2642	0.5142	0.4744	0.7165	0.5684	0.2788
**O-Glycosylation (S,T) Human-only**	No labels	Average	0.6786	0.6282	0.6005	0.8703	0.5306	0.2735	0.6495	0.3970
		STD	0.1358	0.1007	0.0908	0.1292	0.0675	0.1851	0.0437	0.2161
	Labels	Average	0.6853	0.6450	0.6037	0.8227	0.5418	0.2250	0.6388	0.4081
		STD	0.1150	0.1188	0.1361	0.1816	0.1022	0.2597	0.1005	0.3170
		P value	0.9116	0.7495	0.9542	0.5309	0.7870	0.6544	0.7738	0.9319
**Ubiquitination (K) All-organism**	No labels	Average	0.6704	0.2351	0.7751	0.4684	0.2003	0.1935	0.2736	0.8040
		STD	0.0821	0.0885	0.0587	0.1811	0.0790	0.1216	0.0998	0.0660
	Labels	Average	0.6987	0.2685	0.8239	0.3794	0.2365	0.2031	0.2861	0.8672
		STD	0.0732	0.1155	0.0525	0.0809	0.0912	0.0981	0.0866	0.0509
		P value	0.4507	0.5001	0.0799	0.2019	0.3806	0.8557	0.7791	0.0363
**Ubiquitination (K) Human-only**	No labels	Average	0.5840	0.3822	0.5790	0.5027	0.3524	0.1003	0.4038	0.6080
		STD	0.0424	0.0736	0.0438	0.1980	0.0886	0.1003	0.1343	0.1270
	Labels	Average	0.7043	0.5604	0.6652	0.5161	0.4923	0.2579	0.4811	0.7380
		STD	0.0533	0.0728	0.0697	0.1528	0.0771	0.0775	0.0673	0.1418
		P value	0.0001	0.0001	0.0066	0.8746	0.0022	0.0017	0.1462	0.0555
**SUMOylation (K) All-organism**	No labels	Average	0.9036	0.6494	0.8848	0.6987	0.5381	0.5422	0.5879	0.9101
		STD	0.0165	0.0870	0.0368	0.1344	0.1774	0.1271	0.1145	0.0403
	Labels	Average	0.9059	0.6718	0.8912	0.6902	0.5422	0.5425	0.5817	0.9189
		STD	0.0448	0.0468	0.0286	0.2003	0.0971	0.0722	0.0531	0.0410
		P value	0.8853	0.5079	0.6817	0.9165	0.9526	0.9950	0.8848	0.6518
**SUMOylation (K) Human-only**	No labels	Average	0.8603	0.8436	0.7943	0.8040	0.7522	0.5909	0.7744	0.7873
		STD	0.0572	0.0669	0.0395	0.0670	0.0639	0.0763	0.0456	0.0689

	Labels	Average	0.8796	0.8682	0.7969	0.7989	0.7584	0.5942	0.7751	0.7944
		STD	0.0621	0.0701	0.0449	0.0789	0.0684	0.0919	0.0536	0.0708
		P value	0.5014	0.4566	0.8986	0.8854	0.8459	0.9345	0.9775	0.8323
**Acetylation (K) All-organism**	No labels	Average	0.7404	0.1716	0.7974	0.4800	0.1512	0.1816	0.2292	0.8182
		STD	0.0358	0.0388	0.0237	0.0712	0.0281	0.0439	0.0385	0.0261
	Labels	Average	0.7495	0.2000	0.8274	0.4676	0.1804	0.2108	0.2574	0.8514
		STD	0.0298	0.0268	0.0342	0.0451	0.0410	0.0440	0.0451	0.0385
		P value	0.5657	0.0898	0.0461	0.6631	0.0962	0.1769	0.1720	0.0481
**Acetylation (K) Human-only**	No labels	Average	0.7445	0.6427	0.6846	0.6533	0.5970	0.3560	0.6222	0.7067
		STD	0.0221	0.0271	0.0159	0.0489	0.0288	0.0331	0.0204	0.0358
	Labels	Average	0.7970	0.7468	0.7351	0.5957	0.6974	0.4381	0.6406	0.8279
		STD	0.0160	0.0247	0.0161	0.0552	0.0275	0.0350	0.0328	0.0266
		P value	2.60E−05	1.06E−07	2.83E−06	3.10E−02	5.62E−07	7.32E−05	1.73E−01	3.31E−07
**Methylation (K) All-organism**	No labels	Average	0.7490	0.3473	0.9000	0.5250	0.1811	0.2638	0.2619	0.9119
		STD	0.2228	0.3088	0.0389	0.3417	0.1276	0.2046	0.1743	0.0369
	Labels	Average	0.8186	0.3608	0.9055	0.5250	0.2079	0.2875	0.2891	0.9177
		STD	0.1568	0.3203	0.0391	0.3417	0.1270	0.2102	0.1740	0.0360
		P value	0.4546	0.9283	0.7681	1.0000	0.6611	0.8108	0.7442	0.7419
**Methylation (K) Human-only**	No labels	Average	0.6346	0.4533	0.6899	0.3786	0.4557	0.2029	0.3909	0.8042
		STD	0.0833	0.1216	0.0804	0.1692	0.1276	0.1469	0.1251	0.1276
	Labels	Average	0.6470	0.4665	0.7023	0.3653	0.4774	0.2195	0.3964	0.8300
		STD	0.0951	0.0986	0.0741	0.1619	0.1996	0.1951	0.1480	0.0930
		P value	0.7710	0.8023	0.7380	0.8667	0.7865	0.8408	0.9334	0.6302
**Methylation (R) All-organism**	No labels	Average	0.8912	0.5147	0.8559	0.7671	0.4005	0.4826	0.5220	0.8668
		STD	0.0345	0.0658	0.0247	0.0691	0.0730	0.0680	0.0661	0.0250
	Labels	Average	0.8979	0.5413	0.8840	0.7070	0.4569	0.5066	0.5533	0.9043
		STD	0.0227	0.0690	0.0124	0.0528	0.0405	0.0343	0.0341	0.0128
		P value	0.6346	0.4129	0.0092	0.0537	0.0622	0.3624	0.2282	0.0014
**Methylation (R) Human-only**	No labels	Average	0.9106	0.8673	0.8580	0.8154	0.8059	0.6979	0.8087	0.8837
		STD	0.0108	0.0271	0.0182	0.0452	0.0515	0.0394	0.0288	0.0335

	Labels	Average	0.9227	0.8957	0.8665	0.7902	0.8435	0.7133	0.8135	0.9122
		STD	0.0122	0.0245	0.0104	0.0482	0.0491	0.0223	0.0182	0.0311
		P value	0.0385	0.0315	0.2391	0.2679	0.1306	0.3243	0.6774	0.0780
**Pyroglutamylation (Q) All-organism**	No labels	Average	0.9637	0.9476	0.9275	0.8784	0.9176	0.8420	0.8965	0.9548
		STD	0.0194	0.0234	0.0268	0.0587	0.0278	0.0557	0.0348	0.0207
	Labels	Average	0.9644	0.9501	0.9263	0.8863	0.9071	0.8395	0.8955	0.9483
		STD	0.0179	0.0207	0.0180	0.0447	0.0311	0.0376	0.0233	0.0201
		P value	0.9354	0.8149	0.9111	0.7520	0.4634	0.9125	0.9477	0.5114
**Pyroglutamylation (Q) Human-only**	No labels	Average	0.8715	0.8510	0.8039	0.7740	0.7411	0.5960	0.7364	0.8363
		STD	0.1257	0.1379	0.0741	0.1776	0.2239	0.1986	0.1644	0.1220
	Labels	Average	0.8862	0.8777	0.8190	0.7433	0.7868	0.6158	0.7496	0.8718
		STD	0.1295	0.1275	0.0763	0.1584	0.2161	0.1898	0.1557	0.1233
		P value	0.8092	0.6751	0.6744	0.7035	0.6646	0.8315	0.8631	0.5471
**Palmitoylation (C) All-organism**	No labels	Average	0.8872	0.7006	0.8877	0.7122	0.6036	0.5861	0.6418	0.9196
		STD	0.0693	0.1365	0.0304	0.1713	0.1503	0.1444	0.1347	0.0287
	Labels	Average	0.9061	0.7385	0.8980	0.7102	0.6443	0.6125	0.6644	0.9304
		STD	0.0527	0.1031	0.0257	0.1452	0.1387	0.1169	0.1087	0.0301
		P value	0.5234	0.5154	0.4491	0.9790	0.5579	0.6747	0.7004	0.4452
**Palmitoylation (C) Human-only**	No labels	Average	0.8416	0.7399	0.7891	0.6678	0.6392	0.4969	0.6499	0.8336
		STD	0.0553	0.0948	0.0505	0.0862	0.0655	0.0822	0.0645	0.0621
	Labels	Average	0.8716	0.7804	0.8087	0.6550	0.6856	0.5326	0.6646	0.8700
		STD	0.0420	0.0741	0.0543	0.1132	0.1108	0.1211	0.0976	0.0602
		P value	0.2134	0.3275	0.4376	0.7902	0.2968	0.4746	0.7118	0.2225
**Hydroxylation (P) All-organism**	No labels	Average	0.8823	0.6794	0.8519	0.6622	0.6075	0.5407	0.6288	0.8987
		STD	0.0352	0.1171	0.0468	0.1155	0.1097	0.1266	0.1010	0.0345
	Labels	Average	0.9276	0.7847	0.9078	0.7243	0.7873	0.6968	0.7451	0.9526
		STD	0.0256	0.1112	0.0318	0.1012	0.1321	0.1003	0.0846	0.0338
		P value	0.0065	0.0661	0.0093	0.2408	0.0058	0.0099	0.0166	0.0036
**Hydroxylation (P) Human-only**	No labels	Average	0.9387	0.8610	0.9585	0.7736	0.8742	0.7969	0.8136	0.9869
		STD	0.0690	0.1373	0.0317	0.1988	0.1444	0.1756	0.1626	0.0137

	Labels	Average	0.9485	0.8561	0.9601	0.7361	0.8925	0.7842	0.7956	0.9888
		STD	0.0510	0.1134	0.0237	0.1876	0.1189	0.1426	0.1387	0.0113
		P value	0.7350	0.9350	0.9016	0.6855	0.7722	0.8680	0.8036	0.7535
**Hydroxylation (K) All-organism**	No labels	Average	0.8625	0.5945	0.8205	0.6363	0.5202	0.4606	0.5613	0.8538
		STD	0.0462	0.2294	0.0543	0.2311	0.2343	0.2237	0.2193	0.0636
	Labels	Average	0.8796	0.6533	0.8037	0.6396	0.4912	0.4366	0.5403	0.8339
		STD	0.0568	0.2265	0.0697	0.2690	0.2332	0.2453	0.2344	0.0737
		P value	0.4908	0.5913	0.5769	0.9784	0.7948	0.8309	0.8463	0.5481
**Hydroxylation (K) Human-only**	No labels	Average	0.9487	0.9599	0.9718	0.9357	0.9923	0.9289	0.9592	0.9667
		STD	0.0900	0.0807	0.0346	0.1086	0.0231	0.0903	0.0626	0.1000
	Labels	Average	0.9597	0.9577	0.9580	0.9107	0.9723	0.8967	0.9338	0.9576
		STD	0.0665	0.0739	0.0344	0.1192	0.0618	0.0881	0.0669	0.1007
		P value	0.7720	0.9517	0.4070	0.6475	0.3821	0.4546	0.4164	0.8497

S11.3. Using CD-Hit to Lower Test-Set Homology in OGP Datasets

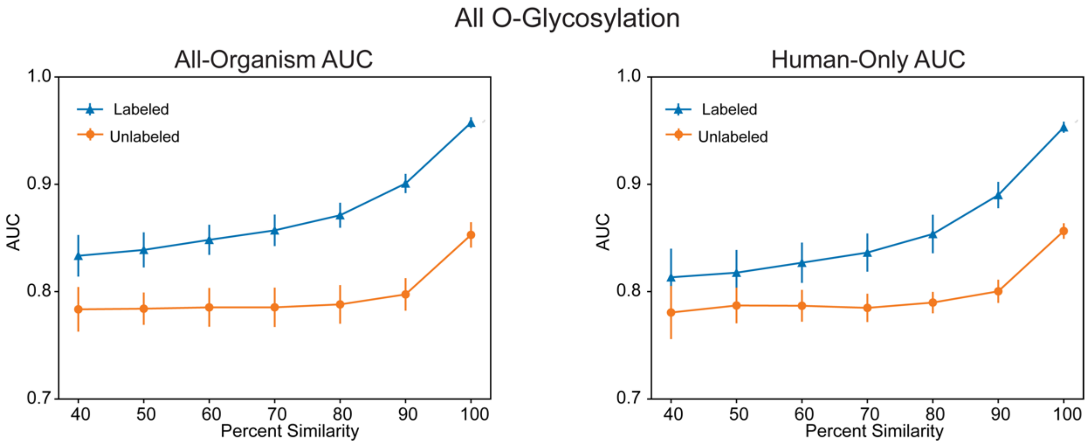

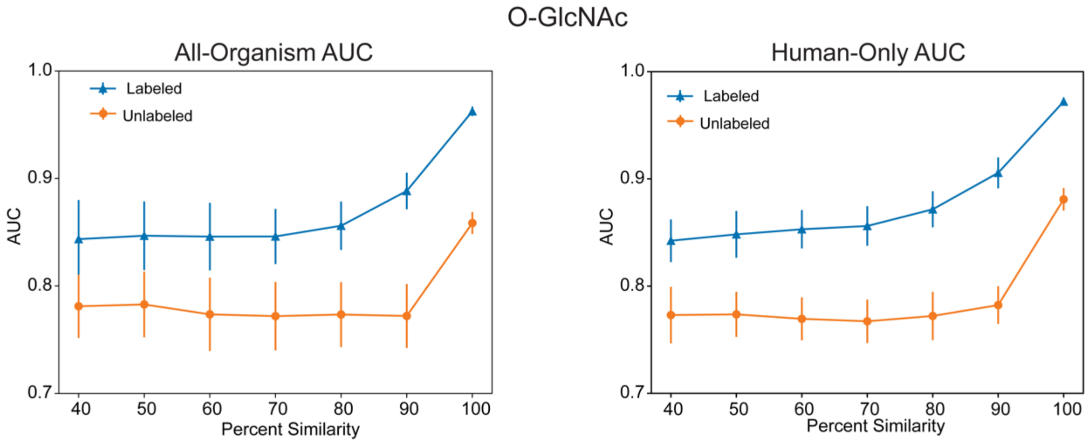

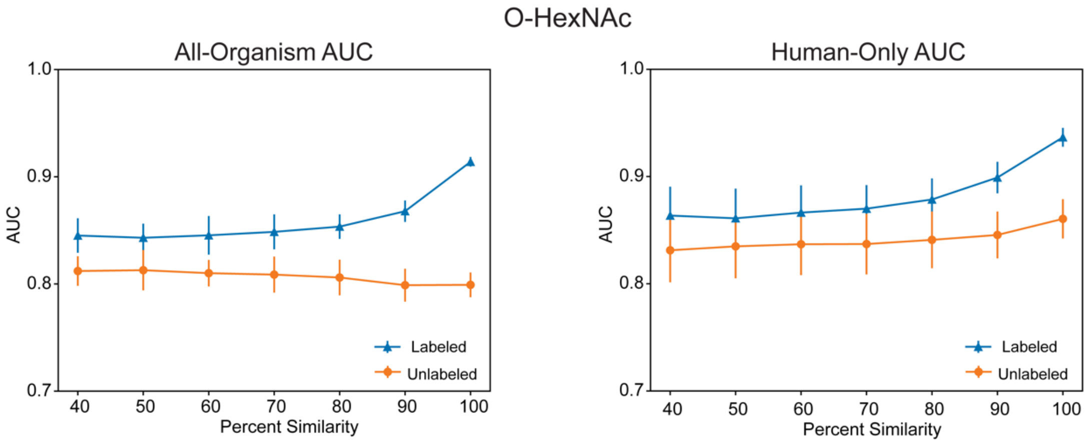

Table S11.3: Model Results at 80% Sequence Similarity Cutoff for OGP Datasets

PTM	Labels	Stat	AUC	AUPRC	Accuracy	Recall	Precision	MCC	F1	Specificity
**All O-glycosylation All-organism**	No labels	Average	0.7881	0.5248	0.7399	0.6604	0.4626	0.3815	0.5431	0.7648
		STD	0.0165	0.0547	0.0151	0.0370	0.0314	0.0329	0.0249	0.0190
	Labels	Average	0.8711	0.7403	0.8499	0.5717	0.7395	0.5586	0.6431	0.9366
		STD	0.0138	0.0282	0.0085	0.0474	0.0420	0.0325	0.0311	0.0142
		P value	1.26E−09	7.33E−08	1.85E−11	3.73E−04	1.81E−11	1.01E−09	7.76E−07	1.19E−13
**All O-glycosylation Human-only**	No labels	Average	0.7898	0.5326	0.7390	0.6620	0.4577	0.3796	0.5400	0.7621
		STD	0.0107	0.0291	0.0186	0.0378	0.0375	0.0331	0.0286	0.0294
	Labels	Average	0.8537	0.7189	0.8465	0.5279	0.7374	0.5339	0.6137	0.9432
		STD	0.0171	0.0249	0.0127	0.0442	0.0306	0.0307	0.0293	0.0092
		P value	9.27E−08	2.88E−11	1.75E−10	2.09E−06	2.25E−12	6.41E−09	3.92E−05	2.84E−09
**GalNAc All-organism**	No labels	Average	0.7734	0.4107	0.7528	0.6071	0.3982	0.3402	0.4781	0.7872
		STD	0.0270	0.0764	0.0165	0.0436	0.0460	0.0299	0.0330	0.0269
	Labels	Average	0.8560	0.6073	0.8453	0.4336	0.6198	0.4297	0.5063	0.9386
		STD	0.0202	0.0631	0.0130	0.0799	0.0644	0.0642	0.0676	0.0159
		P value	1.28E−06	1.44E−05	2.23E−10	5.44E−05	2.56E−07	2.31E−03	2.81E−01	4.31E−10
**GalNAc Human-only**	No labels	Average	0.7721	0.3951	0.7550	0.5633	0.3971	0.3212	0.4612	0.7992
		STD	0.0213	0.0447	0.0281	0.0913	0.0484	0.0571	0.0508	0.0457
	Labels	Average	0.8716	0.6822	0.8754	0.4626	0.7373	0.5177	0.5662	0.9639
		STD	0.0173	0.0340	0.0132	0.0540	0.0625	0.0514	0.0490	0.0119
		P value	3.67E−09	2.65E−11	3.65E−08	1.24E−02	3.44E−10	4.74E−07	3.01E−04	8.67E−07
**HexNAc All-organism**	No labels	Average	0.8060	0.6686	0.7267	0.7479	0.5607	0.4394	0.6400	0.7166
		STD	0.0176	0.0351	0.0179	0.0295	0.0336	0.0294	0.0229	0.0314
	Labels	Average	0.8535	0.7476	0.7932	0.6398	0.6946	0.5175	0.6620	0.8655
		STD	0.0106	0.0377	0.0146	0.0636	0.0457	0.0256	0.0309	0.0326
		P value	5.17E−06	2.24E−04	1.09E−07	5.00E−04	2.13E−06	1.21E−05	1.05E−01	1.12E−08
**HexNAc Human-only**	No labels	Average	0.8409	0.7216	0.7577	0.7603	0.6191	0.4975	0.6806	0.7573
		STD	0.0241	0.0383	0.0204	0.0649	0.0251	0.0454	0.0246	0.0328

	Labels	Average	0.8786	0.7954	0.8192	0.6920	0.7401	0.5834	0.7146	0.8806
		STD	0.0176	0.0219	0.0165	0.0218	0.0440	0.0361	0.0264	0.0243
		P value	1.55E−03	1.75E−04	1.84E−06	1.23E−02	4.30E−06	3.51E−04	1.12E−02	7.77E−08

S11.4. Using CD-Hit to Lower Test-Set Homology in N-Glycosylation Sequon-Specific Datasets

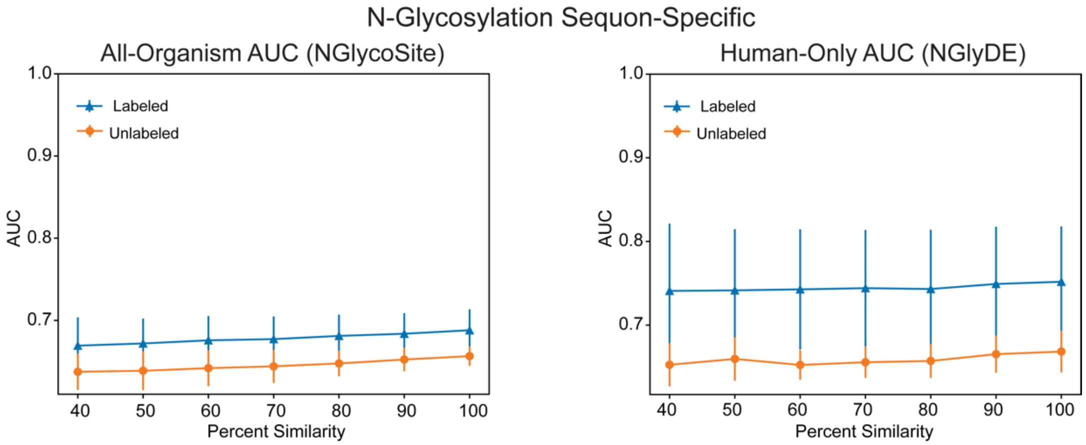

Table S11.4: Model Results at 80% Sequence Similarity for N-Glycosylation Sequon-Specific Datasets

PTM	Labels	Stat	AUC	AUPRC	Accuracy	Recall	Precision	MCC	F1	Specificity
**NGlycoSite All-organism**	No labels	Average	0.6476	0.5283	0.6223	0.1722	0.5777	0.1353	0.2597	0.9184
		STD	0.0142	0.0138	0.0172	0.0598	0.0211	0.0361	0.0730	0.0280
	Labels	Average	0.6811	0.5722	0.6407	0.2712	0.6152	0.2027	0.3750	0.8863
		STD	0.0246	0.0302	0.0155	0.0358	0.0352	0.0337	0.0362	0.0236
		P value	0.0031	0.0017	0.0290	0.0007	0.0153	0.0007	0.0009	0.0172
**NGlyDE Human-only**	No labels	Average	0.6571	0.7737	0.6894	0.9433	0.7017	0.1772	0.8031	0.1678
		STD	0.0182	0.0301	0.0251	0.0490	0.0341	0.0750	0.0176	0.1237
	Labels	Average	0.7431	0.8343	0.7285	0.9346	0.7310	0.3306	0.8197	0.3130
		STD	0.0633	0.0501	0.0277	0.0367	0.0291	0.0778	0.0235	0.0987
		P value	0.0026	0.0073	0.0058	0.6766	0.0662	0.0005	0.1076	0.0136

S11.5. Using CD-Hit to Lower Test-Set Homology in O-GlcNAc Atlas Datasets

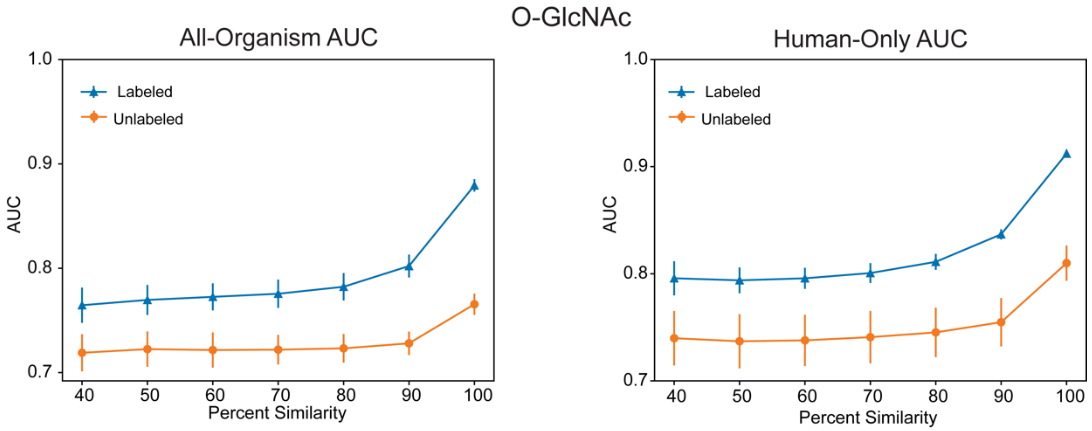

Table S11.5: Model Results at 80% Sequence Similarity Cutoff for O-GlcNAc Atlas Datasets

PTM	Labels	Stat	AUC	AUPRC	Accuracy	Recall	Precision	MCC	F1	Specificity
**O-GlcNAc Human-only**	No labels	Average	0.7453	0.5589	0.7014	0.5516	0.5116	0.3133	0.5288	0.7671
		STD	0.0212	0.0311	0.0199	0.0475	0.0353	0.0353	0.0265	0.0395
	Labels	Average	0.8111	0.6636	0.7688	0.4576	0.6645	0.4063	0.5394	0.9010
		STD	0.0068	0.0228	0.0082	0.0451	0.0412	0.0262	0.0284	0.0222
		P value	2.63E−06	3.55E−07	7.25E−07	4.24E−04	1.33E−07	8.11E−06	4.23E−01	3.66E−07
**O-GlcNAc All-organism**	No labels	Average	0.7232	0.5547	0.6841	0.5510	0.5039	0.2917	0.5237	0.7463
		STD	0.0130	0.0194	0.0164	0.0508	0.0306	0.0169	0.0149	0.0450
	Labels	Average	0.7823	0.6384	0.7458	0.4906	0.6216	0.3799	0.5472	0.8627
		STD	0.0118	0.0227	0.0078	0.0438	0.0209	0.0260	0.0288	0.0177
		P value	8.42E−09	1.42E−07	1.53E−07	1.49E−02	5.56E−08	3.04E−07	4.73E−02	1.20E−05

S12. Method Application to Models

S12.1. TYOM: Train your own model instructions

To allow for models to be trained using this method of encoding known PTM locations we have developed Jupyter Notebooks to train models for different contexts. To illustrate this, we have detailed this method using a dataset containing phosphorylation before and after sprinting by Parker and coworkers (Blazev *et al.* 2022). Using this functionality has the following three requirements:

1. A list of positive sites for the dataset in an Excel spreadsheet with one column corresponding to the “UniProt ID” and another to the “Position” in the protein.
2. A text file in FASTA format containing the reference proteins and their sequences, with the name formatted as such: >tr|UniProtID|…
3. A python environment set up with the necessary packages including Jupyter Notebook (see https://github.com/clair-gutierrez/sitetack/tree/main/train for more details)

Example: Looking at Pre- and Post-Sprinting Phosphorylation Datasets (Blazev *et al.* 2022)

The phosphorylation dataset from Parker and coworkers contains phosphoproteomic analysis of human skeletal muscle before and after different exercise modalities. We chose to use only the data for “sprint” as it showed the largest change in phosphorylation sites, which was detected with a mass error of less than or equal to 5 ppm. We partitioned this dataset into 3 portions:

- Phosphosites that were present in both conditions (PostSprint versus PreSprint q-value >0.05)
- Phosphosites enriched pre-sprint (PostSprint versus PreSprint q-value ≤0.05 and log(PostSprint/PreSprint) > 0)
- Phosphosites enriched post-sprint (PostSprint versus PreSprint q-value ≤0.05 and log(PostSprint/PreSprint) < 0)

We specified that the PreSprint dataset would have all phosphosites present in both conditions and enriched in pre-sprint and that the PostSprint dataset would have all phosphosites present in both conditions and enriched in post-sprint. We also obtained a reference proteome from UniProt that contained only the proteins that had a phosphosite in this study (to avoid labeling a protein site as negative if it just was not detected given that these data are from a single report).

Using these datasets we trained four models (with and without phosphorylation events labeled, pre- and post-sprint) and evaluated the prediction accuracy of these models with and without phosphorylation locations in the pre-and post-sprint conditions. We chose an example protein, troponin C, which is reported to be phosphorylated at Ser92 (Ochoa *et al.* 2020) and was not present in our training set. We found that, at Ser92, there was a large difference in prediction in the models with nearby phosphorylation locations between the pre- and post-sprint conditions, which was not captured by the models without phosphorylation locations. This indicates that these models were better able to capture changes in different situations (*e.g.*, pre- and post-exercise).

S12.2. Example: Training models to predict phosphorylation in different contexts

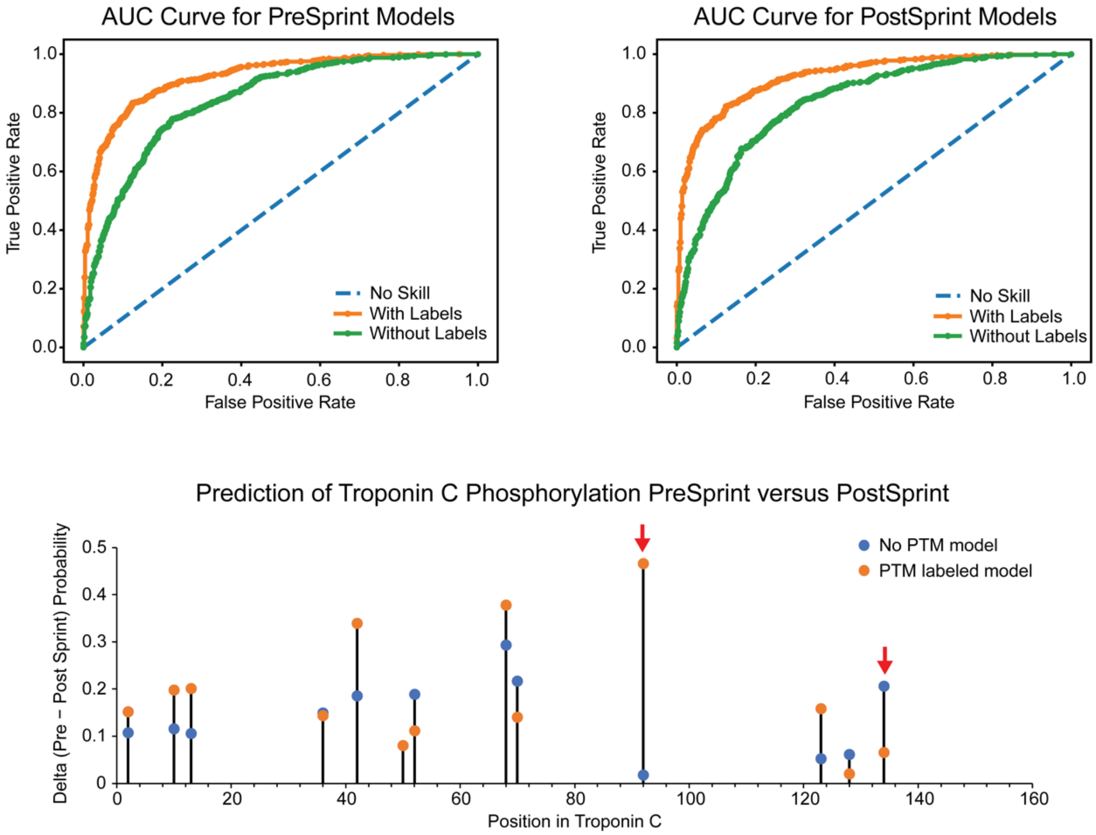

Figure S12.2: Example TYOM on Blazez dataset. Top: ROC curves for models trained on exercise datasets before (PreSprint) and after (PostSprint) sprinting, where having phosphorylation sites labeled improves prediction. Bottom: Troponin-C (TNNC2), which is an important protein in skeletal muscle function that was not present in the training set, was chosen as a case study (van de Locht *et al.* 2021). Phosphorylation is reported at Ser92 and Ser134 and highlighted with red arrows (Ochoa *et al.* 2020). When the difference between the pre- and post-sprint conditions are plotted for each model at Ser92, it is evident that there is a large difference in predictions only in the models that include phosphorylation information.

S13. References

Blazev, R, Carl, CS, Ng, YK *et al.* Phosphoproteomics of three exercise modalities identifies canonical signaling and C18ORF25 as an AMPK substrate regulating skeletal muscle function. *Cell Metab* 2022;**34**:1561–77.e9.

Fu, L, Niu, B, Zhu, Z *et al.* CD-HIT: accelerated for clustering the next-generation sequencing data. *Bioinformatics* 2012;**28**:3150–2.

Huang, J, Wu, M, Zhang, Y *et al.* OGP: a repository of experimentally characterized O-glycoproteins to facilitate studies on O-glycosylation. *Genom Proteom Bioinform* 2021;**19**:611–8.

Ma, J, Li, Y, Hou, C *et al.* O-GlcNAcAtlas: a database of experimentally identified O-GlcNAc sites and proteins. *Glycobiology* 2021;**31**:719.

Ochoa, D, Jarnuczak, AF, Viéitez, C *et al.* The functional landscape of the human phosphoproteome. *Nat Biotechnol* 2020;**38**:365–73.

Pakhrin, SC, Aoki-Kinoshita, KF, Caragea, D *et al.* DeepNGlyPred: a deep neural network-based approach for human N-linked glycosylation site prediction. *Molecules* 2021;**26**:7314.

Pakhrin, SC, Pokharel, S, Aoki-Kinoshita, KF *et al.* LMNglyPred: prediction of human N-linked glycosylation sites using embeddings from a pre-trained protein language model. *Glycobiology* 2023;**33**:411–22.

Sugiyama, N, Imamura, H and Ishihama, Y. Large-scale discovery of substrates of the human kinome. *Sci Rep* 2019;**9**:1–12.

Sun, S, Hu, Y, Ao, M *et al.* N-GlycositeAtlas: a database resource for mass spectrometry-based human N-linked glycoprotein and glycosylation site mapping. *Clin Proteomics* 2019;**16**:1–11.

van de Locht, M, Donkervoort, S, de Winter, JM *et al.* Pathogenic variants in TNNC2 cause congenital myopathy due to an impaired force response to calcium. *J Clin Invest* 2021;**131**:e145700.

Wang, D, Liu, D, Yuchi, J *et al.* MusiteDeep: a deep-learning based webserver for protein post-translational modification site prediction and visualization. *Nucleic Acids Res* 2020;**48**:W140–6.
